# Metagenomic and PCR-Based Diversity Surveys of [FeFe]-Hydrogenases Combined with Isolation of Alkaliphilic Hydrogen-Producing Bacteria from the Serpentinite-Hosted Prony Hydrothermal Field, New Caledonia

**DOI:** 10.3389/fmicb.2016.01301

**Published:** 2016-08-30

**Authors:** Nan Mei, Anne Postec, Christophe Monnin, Bernard Pelletier, Claude E. Payri, Bénédicte Ménez, Eléonore Frouin, Bernard Ollivier, Gaël Erauso, Marianne Quéméneur

**Affiliations:** ^1^Aix Marseille Univ, Université de Toulon, CNRS, IRD, MIOMarseille, France; ^2^GET UMR5563 (Centre National de la Recherche Scientifique/UPS/IRD/CNES), Géosciences Environnement ToulouseToulouse, France; ^3^Institut pour la Recherche et le Développement (IRD) Centre de Nouméa, MIO UM 110Nouméa, Nouvelle-Calédonie; ^4^Institut de Physique du Globe de Paris, Sorbonne Paris Cité, Univ Paris Diderot, Centre National de la Recherche ScientifiqueParis, France

**Keywords:** hydrogen, microbial diversity, hydrogen producers, serpentinization, *hydA* genes, [FeFe]-hydrogenase, metagenomics

## Abstract

High amounts of hydrogen are emitted in the serpentinite-hosted hydrothermal field of the Prony Bay (PHF, New Caledonia), where high-pH (~11), low-temperature (< 40°C), and low-salinity fluids are discharged in both intertidal and shallow submarine environments. In this study, we investigated the diversity and distribution of potentially hydrogen-producing bacteria in Prony hyperalkaline springs by using metagenomic analyses and different PCR-amplified DNA sequencing methods. The retrieved sequences of *hydA* genes, encoding the catalytic subunit of [FeFe]-hydrogenases and, used as a molecular marker of hydrogen-producing bacteria, were mainly related to those of *Firmicutes* and clustered into two distinct groups depending on sampling locations. Intertidal samples were dominated by new *hydA* sequences related to uncultured *Firmicutes* retrieved from paddy soils, while submarine samples were dominated by diverse *hydA* sequences affiliated with anaerobic and/or thermophilic submarine *Firmicutes* pertaining to the orders *Thermoanaerobacterales* or *Clostridiales*. The novelty and diversity of these [FeFe]-hydrogenases may reflect the unique environmental conditions prevailing in the PHF (i.e., high-pH, low-salt, mesothermic fluids). In addition, novel alkaliphilic hydrogen-producing *Firmicutes* (*Clostridiales* and *Bacillales*) were successfully isolated from both intertidal and submarine PHF chimney samples. Both molecular and cultivation-based data demonstrated the ability of *Firmicutes* originating from serpentinite-hosted environments to produce hydrogen by fermentation, potentially contributing to the molecular hydrogen balance *in situ*.

## Introduction

Hydrogen (H_2_) can be naturally produced by both geochemical and biological processes in various environments. Geochemically, H_2_ can be generated during the serpentinization of ultramafic rocks by the reduction of water coupled to the oxidation of ferrous Fe contained in olivines and pyroxenes (Schrenk et al., 2013). This reaction is accompanied by the production of exceedingly alkaline waters (pH up to 12). Produced H_2_ constitutes a large reservoir of energy with the capacity to sustain the development of a wide range of chemolithoautotrophic microorganisms. As an illustration, in serpentinite-hosted ecosystems, H_2_ has been shown to be consumed by microorganisms such as anaerobic hydrogenotrophs (e.g., methanogens) and aerobic H_2_-oxidizing *Betaproteobacteria* (i.e., “*Serpentinomonas*” spp.; Brazelton et al., 2006; Tiago and Veríssimo, 2013; Quéméneur et al., 2014, 2015; Suzuki et al., 2014). In addition to H_2_ production resulting from abiotic reactions, H_2_ could be produced biologically by various types of microorganisms in these anoxic serpentinite-hosted environments. Among them, the *Firmicutes* phylum and especially the *Clostridiales* spp. are recognized as potential fermentative H_2_-producing bacteria (Xing et al., 2008; Quéméneur et al., 2011). However, the distribution and role of these microorganisms in the H_2_ budget of serpentinization-related systems have been scarcely addressed; so far, only two studies have investigated the potential of anaerobic microorganisms to biologically produce H_2_ in these hyperalkaline environments (Brazelton et al., 2012; Mei et al., 2014).

Biological H_2_ production is performed by phylogenetically and physiologically diverse groups of *Bacteria* and *Archaea* (i.e., anaerobes, facultative anaerobes, and photosynthetic bacteria). This process is carried out by hydrogenases that catalyze the reversible oxidation of H_2_ and are divided into two major phylogenetically distinct classes: [NiFe]-hydrogenases and [FeFe]-hydrogenases (Vignais and Colbeau, 2004; Lubitz et al., 2014; Peters et al., 2015). [NiFe]-hydrogenases are widely distributed among the *Bacteria* and *Archaea* domains, whereas [FeFe]-hydrogenases are primarily found in anaerobic bacteria of the orders *Clostridiales, Thermotogales*, and the family *Desulfovibrionaceae* (Vignais et al., 2001). While [NiFe]-hydrogenases are generally involved in H_2_ consumption, [FeFe]-hydrogenases are usually involved in H_2_ production *in vivo* (Vignais and Colbeau, 2004), with nonetheless some exceptions including electron-bifurcating [FeFe]-hydrogenases (Schut and Adams, 2009; Wang et al., 2013; Poudel et al., 2016). The *hydA* genes encoding the catalytic subunit of [FeFe]-hydrogenases have been used as a pertinent molecular marker to monitor compositional changes of bacterial H_2_-producers in fermentation bioreactors (Xing et al., 2008; Quéméneur et al., 2010, 2011). The genetic diversity of *hydA* genes and associated H_2_ production potential have also been studied by culture-independent approaches in various ecosystems including extreme environments, such as saline microbial mats of Guerrero Negro (Mexico) or geothermal springs of Yellowstone National Park (USA) discharging acidic, neutral or alkaline fluids (Boyd et al., 2009, 2010) to provide a more exhaustive picture of H_2_-producing community.

The hydrothermal field of the Prony Bay (PHF, New Caledonia, South Pacific) comprises several intertidal and shallow submarine hyperalkaline springs located at less than 50 m below sea level (mbsl). Similarly to the deep-sea Lost City hydrothermal field (LCHF) located at ~800 mbsl, off the Mid-Atlantic Ridge (30°N), PHF relies on an serpentinizing basement and discharges into the seawater high pH (~11) fluids enriched in hydrogen (H_2_: 19–24% vol in free gas) and methane (CH_4_: 6–13% vol in free gas; Kelley et al., 2005; Monnin et al., 2014). When alkaline fluids mix with seawater, precipitation in the form of calcium carbonates (CaCO_3_), and brucite [Mg(OH)_2_] occurs (Launay and Fontes, 1985), forming chimneys reaching up to tens of meters in height. Although PHF and LCHF display similar geochemical and mineralogical features, PHF is unique in that its hydrothermal features release low-temperature (< 40°C) and low-salinity fluids in a shallow submarine environment. Recently, molecular microbial surveys based on 16S rRNA genes analysis provided evidence of an abundant and highly diverse bacterial community inhabiting these hydrothermal chimneys with a peculiar emphasis for members of the *Firmicutes* (Quéméneur et al., 2014; Postec et al., 2015), as also evidenced in other serpentinization-related environments (Brazelton et al., 2013; Miller et al., 2016). Several anaerobic bacterial strains belonging to this phylum, and especially to the *Clostridiales* order, have been successfully isolated from the PHF submarine chimneys and described (Ben Aissa et al., 2014, 2015; Mei et al., 2014; Bes et al., 2015). To our knowledge, these bacteria are to date the unique anaerobic isolates reported from serpentinite-hosted environments. Those may impact the H_2_ budget.

The main goal of this study was to determine the diversity and distribution of the [FeFe]-hydrogenase encoding genes, considered as a molecular marker of H_2_-producing bacteria that may contribute to hydrogen production in different sites of the PHF located in intertidal or shallow submarine zones. [FeFe]-hydrogenases encoding genes related to those belonging to the order *Clostridiales*, phylum *Firmicutes*, were largely detected using both metagenomic analyses and PCR amplification and subsequent sequencing of *hydA* genes. Attempts to cultivate and isolate new alkaliphilic hydrogen-producing *Clostridiales* strains from these alkaline serpentinite-hosted environments were also successful.

## Materials and methods

### Site description

**Figure 1:**
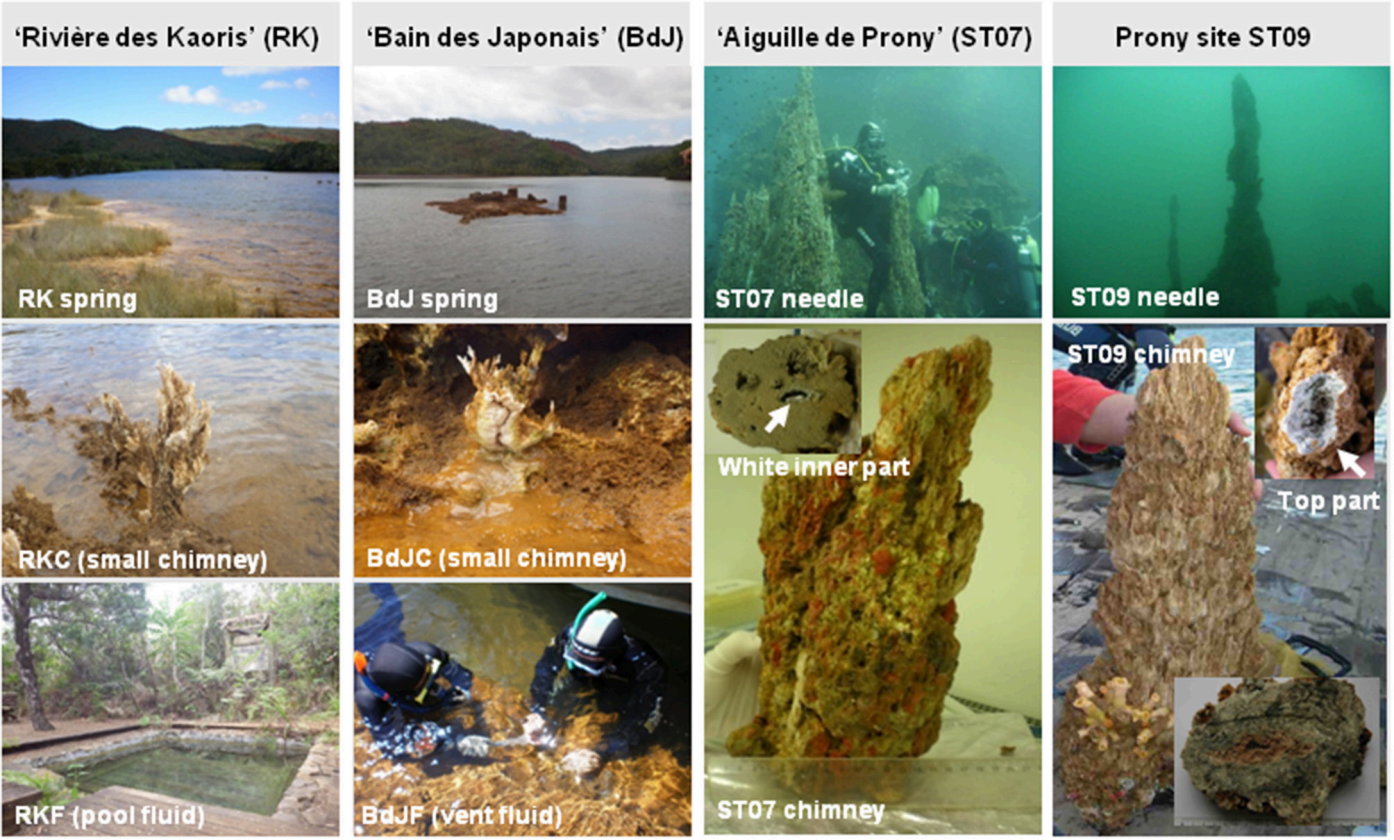
**Intertidal and submarine hydrothermal sources of Prony Hydrothermal Field (PHF) in New Caledonia**. Photographs show the sampling sites: “Rivière des Kaoris” (RK), “Bain des Japonais” (BdJ), “Aiguille de Prony” (ST07), and the Prony ST09 site. Suffixes “C” and “F” in site names stand, respectively, for chimney and fluid.

The samples used for PCR-based molecular analyses as well as microbial cultures in this study were collected in October 2012 at three different sites in the Prony Bay: (i) “Rivière des Kaoris” (RK), located in the Eastern end of the Carenage Bay (22°17.969′S, 166°51.709′E), (ii) “Bain des Japonais” (BdJ), located in the Western branch of the Carenage Bay (2°17.970′S, 166°51.708′E), and (iii) “Aiguille de Prony” (also referred to as ST07), located in the North of the Bay (22°19.796′S, 166°50.058′E; Figure 1; Quéméneur et al., 2014). The samples used for metagenomic analyses were collected in November 2005 at the deepest submarine PHF site ST09 (22°21.653S, 166°52.777E; Figure 1; Postec et al., 2015). Site locations are indicated on the map displayed in Monnin et al. (2014).

The RK site is composed of large travertines (covered by seawater in high tide), close to an artificial pool (~3 m long, ~2 m wide, and ~1.5 m deep) fed by hyperalkaline fluids (pH 10.9) and it is located on the coast above sea level. The BdJ site is a carbonated platform located on the foreshore and thus uncovered at low tide. The tidal range in the Prony bay is about 1 m. In contrast, the ST07 site is a submarine edifice, 35 m high, composed of carbonated chimneys sampled by scuba divers at 16 mbsl (Figure 1). At the ST09 site, an active and partially coral encrusted submarine chimney with white top was collected at 43 mbsl (Postec et al., 2015).

In PHF sites, H_2_ was the second most dominant gas (followed by CH_4_) after N_2_ (Monnin et al., 2014; Deville and Prinzhofer, 2016). The range of the H_2_, CH_4_, and N_2_ gas contents collected at the intertidal PHF sites (RK and BdJ) were 19–24%V, 6–13%V, and 67–69%V, respectively (Monnin et al., 2014). The fluids discharged by PHF sites had high pH-values ranging from 10.1 (ST07) to 11.1 (BdJ). The temperature of fluids measured at sampling sites ranged from 30.8°C (RK) to 37°C (BdJ). The geological context and detailed composition of waters and gases of the PHF are given by Monnin et al. (2014).

### Sample collection

Both ST07 and ST09 submarine chimneys were transversally cut from the top part generating sections of ~10–30 cm in diameter (Figure 1). The most central parts of sections of the ST07 and ST09 carbonated chimneys and the small BdJ (BdJC) and RK (RKC) chimneys (~10 cm in height and ~3 cm in diameter) were crushed and (i) stored in sterile Falcon^TM^ tubes at −80°C prior to DNA extraction or (ii) placed overnight at 4°C in a hermetically-sealed serum bottle with a nitrogen gas headspace prior to H_2_-producing enrichments. Fluid end-member samples (checked by pH-values >10.5 and salinity < 2 g/L) were collected from BdJ vents (BdJF) and RK pool (RKF) using 60 mL sterile syringes, pooled in sterile plastic bottles, and stored in a portable icebox until filtration, a few hours after sampling. The fluids (2 L) were filtered through 0.2 μm pore-size Isopore^TM^ polycarbonate membrane filters (Millipore). The filters were then kept overnight at 4°C before cultivation or at −80°C prior to DNA extraction.

### Hydrogen-producing enrichments

H_2_-producing enrichments were performed in duplicate using Hungate tubes containing the following basal medium components (per liter of distilled water): 0.1 g KH_2_PO_4_, 0.1 g K_2_HPO_4_, 0.1 g NH_4_Cl, 2 g NaCl, 0.1 g KCl, 0.1 g CaCl_2_.2H_2_O, 0.1 g yeast extract (Difco), 0.1 g cysteine hydrochloride and 10 mL trace mineral element solution (Balch et al., 1979). The initial pH was adjusted to 9.5 with NaOH. This pH above 9 can select alkalophilic microorganisms and enhances their diversity that is expected to be lower if pH is adjusted to 11. The basal medium was boiled and cooled down to room temperature under a continuous O_2_-free N_2_-flush. Five milliliter of this medium was then dispensed into Hungate tubes under anaerobic conditions and autoclaved (45 min, 120°C). Prior to inoculation, the following sterile solutions were injected in each tube: 0.1 mL of 2% Na_2_S.9H_2_O (reducing agent) and 0.1 mL of 8% Na_2_CO_3_ (to adjust and buffer the pH); biotrypcase (2 g/L), yeast extract (2 g/L) and glucose (2 g/L) were used as substrates. This final medium used for H_2_-producing enrichments was referred to as BYG medium.

The tubes were inoculated with 0.5 g of crushed chimney rock (named BdJC, RKC, or ST07 for the “Bain des Japonais,” “Rivière des Kaoris,” and “Aiguille de Prony” sample, respectively) or polycarbonate filters from 0.2 μm fluids filtration of 2 L of fluid (named respectively BdJF and RKF for the “Bain des Japonais” and “Rivière des Kaoris” samples). The suspensions were serially diluted in decimal steps using the same media (up to 10^−6^) and then incubated for 1 month at 37 and 55°C. Microbial growth was determined by measuring the increase in turbidity at 600 nm after insertion of Hungate tubes into the cuvette holder of a UV-visible spectrophotometer (Cary 50, Varian). One hundred microliters of the headspace was periodically collected in order to determine the H_2_ content in the gas phase. Gas composition (H_2_, O_2_, CH_4_, and CO_2_) was determined using a Shimadzu GC 8A gas chromatograph (GC) equipped with a thermal conductivity detector (GC/TCD; Alltech, USA). The H_2_ production was expressed in mM (mmol per L of culture). One to ten milliliters of the cultures were collected at the end of the experiments and then centrifuged (10,000 g, 10 min). The supernatants were stored at −20°C for further chemical analysis. The concentration of carbohydrates and soluble end-products of metabolism was determined by high-pressure liquid chromatography (HPLC) analysis and refractometric detection (Thermo Separation Products). Details of analytical operating conditions were previously described by Mei et al. (2014). All analyses were conducted in duplicate.

Positive H_2_-producing cultures were subcultured into the same BYG liquid medium, and then purified by repeated use of the Hungate roll-tube method (Hungate, 1969) with medium solidified with 1.6% (w/v) agar (Difco). Several colonies that had developed were picked and cultured in BYG liquid medium. The pure cultures were identified after DNA extraction followed by 16S rRNA gene amplification, cloning, and sequencing (see below).

### DNA extraction

DNA was extracted following the protocol previously described by Quéméneur et al. (2014). The matrices used were: 0.5 g of crushed carbonate chimneys or, one quarter of a 47 mm diameter polycarbonate filter or the bacterial cell pellet from 10 to 15 mL of positive H_2_-producing cultures and reference strains (used as control in PCR tests, see below). The concentration of DNA extracts was measured using Qubit® fluorometer (Invitrogen).

### Metagenomic analyses

Two DNA samples from the deepest submarine PHF chimney (ST09) were used to obtain PHF metagenomes. Metagenomic libraries were prepared using 25 ng of DNA per sample. The construction kit was the Ultralow Ovation system (NuGen). The multiplex ligation adaptor mixes were L2DR_BC9 and L2DR_BC7. Fifteen PCR cycles were applied to generate sufficient materials for sequencing. Paired-end sequencing (2 × 100 nt) was performed on an Illumina HiSeq 1000 at Marine Biological Laboratory, Woods Hole, MA.

All merged paired-end reads for ST09 samples are available on the MG-RAST server (Meyer et al., 2008) under ID 4550491.3 (P27) and ID 4550492.3 (P28). There were 5,392,044 and 7,933,927 sequence reads from P27 and P28 metagenomes, respectively.

The taxonomic annotation of these merged paired-end reads was conducted in MG-RAST server. Briefly, a phylogenomic reconstruction of the ST09 samples was computed by using both the phylogenetic information contained in the SEED nr database and the similarities to the ribosomal RNA database (Meyer et al., 2008).

The PHF metagenomes (P27 and P28) were screened for *hydA* genes encoding the large subunit of the [FeFe]-hydrogenase. The amino acid sequences of HydA proteins (pfam02906) were retrieved from the NCBI protein database (in May 2014) and used locally as a specific database for similarity searches using PHF metagenomes as query with BLASTX tool (version 2.2.25+) with default algorithm parameters and an *E*-value cutoff of 10^−5^. The merged paired-end reads related to *hydA* genes with significant hits were then extracted from MG-RAST, aligned and then clustered into OTUs using an 80% identity threshold with UCLUST algorithm. The representative of each OTU was then searched with BLASTX against the NCBI nr database. The metagenomes sequences are available in NCBI public database (SRA) under accession numbers: SRX748869 (P27) and SRX748870 (P28).

### PCR amplification, cloning, sequencing, and phylogenetic analyses

Three degenerate primer sets were tested from environmental samples and reference strains to target *hydA* genes encoding [FeFe]-hydrogenases: (i) hydF1/hydR1 (Xing et al., 2008), (ii) FeFe-272F/ FeFe-427R (Boyd et al., 2009), and (iii) HydH1f/ HydH3r (Schmidt et al., 2010). The bacterial 16S rRNA genes from H_2_-producing cultures were amplified using the primer set 27F/907R (Lane, 1991). Each PCR mixture (20 μL) contained 1X GoTaq® Hot Start Green Master Mix (Promega), 200 nM of each primer, and 1–10 ng of genomic DNA. Reactions were conducted in a T100 thermal cycler (Bio-rad). The 16S rRNA genes were amplified as follows: 94°C for 2 min, followed by 30 cycles performed at 94°C for 30 s, 50°C for 30 s, and 72°C for 30 s, with a final extension at 72°C for 10 min. The *hydA* genes were amplified as described above, except that 40 cycles were performed and that hybridization were carried out from 50 to 62°C using a temperature gradient in order to determine the optimal annealing temperature of each primer pair. The PCR products were then checked by electrophoresis on a 1% agarose gel containing sight DNA stain. Positive and blank controls were carried out for all amplifications.

Triplicate PCR products were pooled and purified with NucleoSpin Gel and PCR Clean-up kit (Macherey-Nagel), according to the manufacturer's instructions. The purified PCR products were ligated into a pGEM®-T *Easy* vector and cloned into JM109 *Escherichia coli* competent cells (Promega), according to the manufacturer's instructions. Sequencing of the inserts was performed by Beckman Genomics (Takeley, Essex, UK) on plasmid minipreps using primers 27F and T7 for the 16S rRNA gene and the *hydA* genes, respectively.

Nonchimeric 16S rRNA gene sequences (checked with the online Bellerophon program) and translated *hydA* sequences were aligned using Muscle implemented in the MEGA6 software (Tamura et al., 2013). The program mothur was used to group sequences into operational taxonomic units (OTUs) based on 97% identity for 16S rRNA genes and 80% identity for HydA sequences (Baba et al., 2014). Mothur was also used to estimate richness (Chao, 1984) and to compute diversity indices (Shannon and Weaver, 1949; Simpson, 1949) for each *hydA* clone library. The Good's coverage C of each clone library was calculated according to the equation: C = 1–(n/N) where n is the number of OTU and N is the total number of clones in the library (Good, 1953). At least one HydA sequence deriving from translated *hydA* sequence and representing each OTU, designed as phylotype was further aligned using Muscle with related sequences retrieved from NCBI databases using BLAST tools. The MEGA6 software was also used for phylogenetic tree construction by the Maximum Likelihood method using bootstrap analysis on 1000 replicates (Felsenstein, 1985). The *hydA* gene and HydA sequences from environmental samples were deposited in the Genbank database under the accession numbers KT357617-KT357637. The 16S rRNA genes from bacterial cultures were deposited in the Genbank database under the accession numbers KR349722 (3b), KJ626326 (PROH2), and KR349723 (BJ2).

### Pyrosequencing analyses of bacterial 16S rRNA gene fragments

The mixtures of 16S rRNA gene amplicons were generated from a 341F/815R bacterial primer set, as previously described by Dowd et al. (2008), and were sequenced on a 454 GS-FLX Titanium sequencer (Roche Life Sciences, USA) by the Molecular Research Laboratory (Texas, USA). Raw sequence data generated by pyrosequencing were uploaded into QIIME 1.8.0 and processed as described by Caporaso et al. (2010). Briefly, sequences were qualitatively trimmed, aligned using Pynast and clustered into operational taxonomic units (OTUs) using a 97% identity threshold with UCLUST (Edgar, 2010). Taxonomic assignment was carried out with the RDP Classifier (Wang et al., 2007) with a minimum bootstrap confidence of 80%. BLAST searches against a non-redundant nucleotide database were performed for a representative sequence of each OTU. The OTU richness was assessed using non-parametric richness estimators. The Shannon and Simpson's diversity indices were also calculated. The 16S rRNA genes from environmental samples were deposited in the Genbank database under the accession numbers KT344933-KT344984.

## Results

### [FeFe]-hydrogenase gene diversity in submarine samples using metagenomic analysis

The two PHF metagenomes obtained from the submarine ST09 site contained numerous sequences related to [FeFe]-hydrogenases (Tables S1, S2). A total of 292 and 1386 merged paired-end reads were assigned to [FeFe]-hydrogenases in samples P27 and P28, respectively. These 1678 whole putative *hydA* reads represented 0.011% of the total reads in average (0.005 and 0.017% for P27 and P28, respectively). The number of OTUs observed in P27 (*n* = 113; Table S1) was lower than that found in P28 (*n* = 395; Table S2) in agreement with the respective size of the two metagenomes.

**Figure 2:**
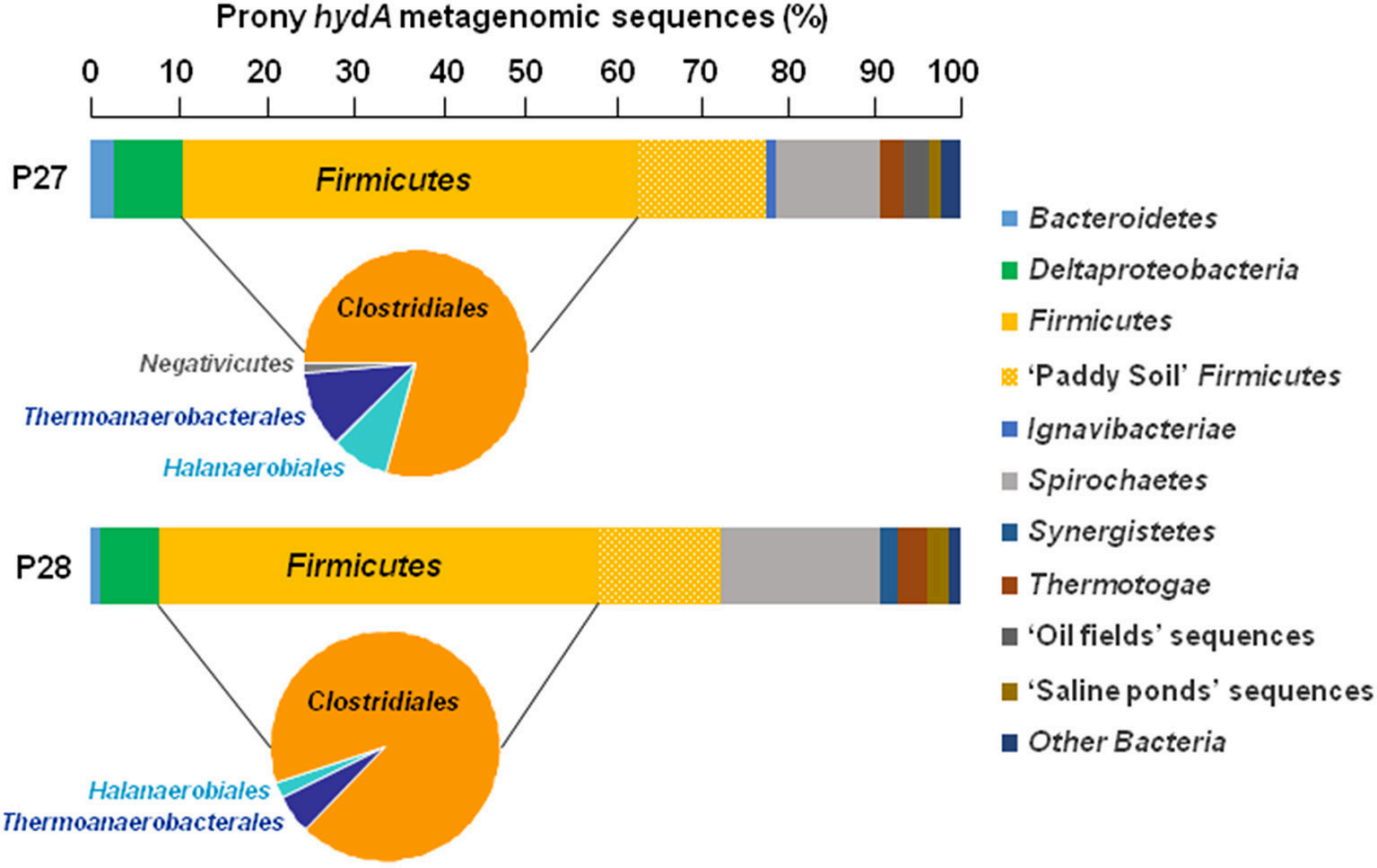
**Distribution and taxonomic assignments of putative *hydA* gene sequences encoding the catalytic subunit of [Fe-Fe] hydrogenases in Prony Hydrothermal Field metagenomes**. The histogram shows the relative abundance of major *hydA* phyla in the total *hydA* metagenomic sequences (*n* = 1678) and pie charts show distributions of “*Firmicutes* HydA” classes in the metagenomes P27 and P28 obtained from ST09 site.

**Figure 3:**
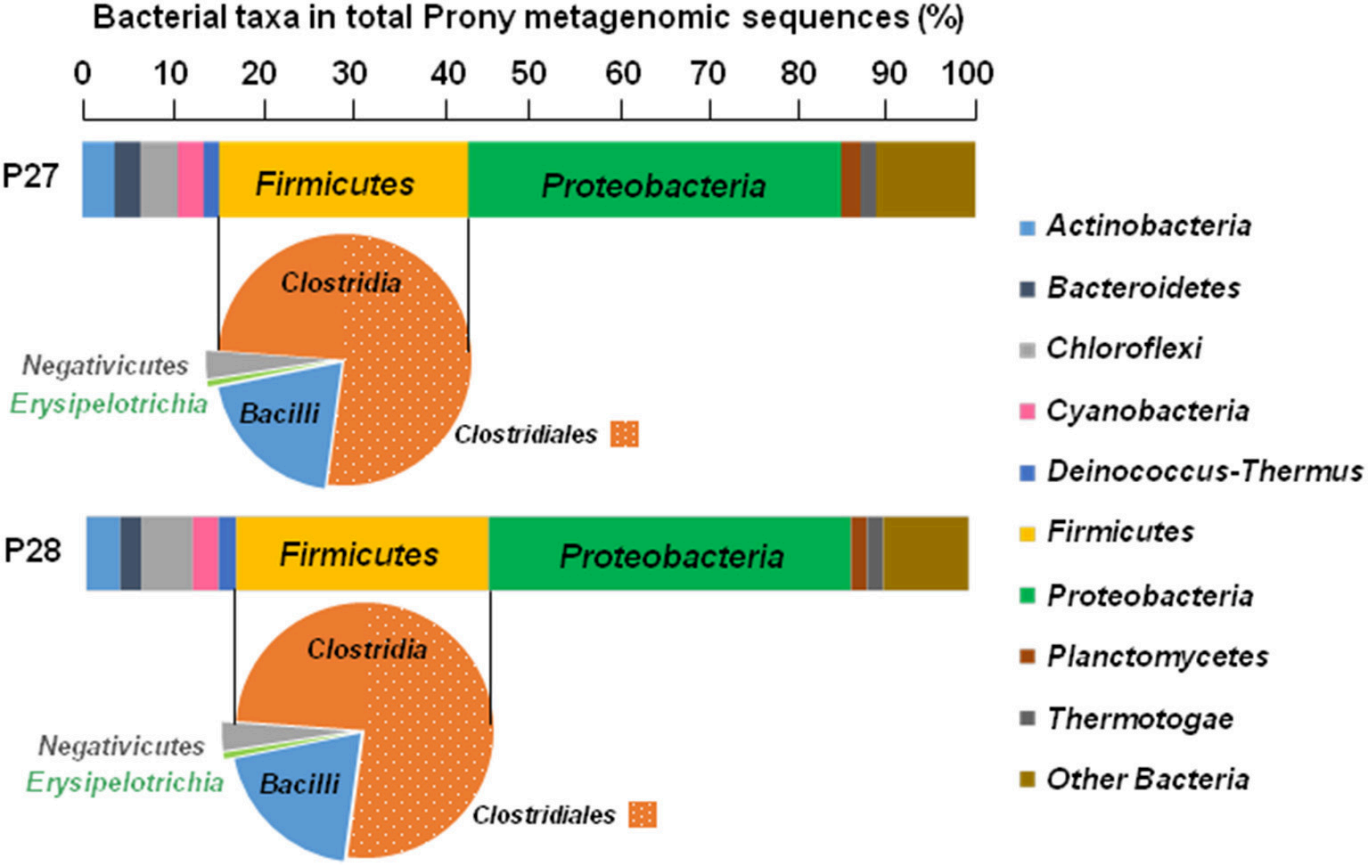
**Distribution and taxonomic assignments of total bacterial sequences in Prony Hydrothermal Field metagenomes P27 and P28 obtained from ST09 site**. The histogram shows the relative abundance of major bacterial phyla in the total bacterial metagenomic sequences and pie charts show distributions of major *Firmicutes* classes.

Whichever the PHF metagenome considered, the HydA OTUs were mainly related to *Firmicutes* (64.6 and 66.4% of the total HydA reads in P27 and P28, respectively), in which *Clostridiales*-related sequences were dominant (Figure 2), in agreement with BLAST assignment of total bacterial merged paired-end reads which are widely associated with those of *Firmicutes* (28.3 and 28.8% of the total bacterial reads in P27 and P28, respectively; Figure 3). Among total merged paired-end reads related to *Firmicutes* sequences, *Clostridia*, and *Bacilli* accounted for the two major classes (95 and 96% of the *Firmicutes* reads in P27 and P28, respectively) and *Clostridiales*-related sequences were dominant (52% of the *Firmicutes* reads; Figure 3).

The majority of HydA OTUs had close phylogenetic relationships with *Clostridium*- and *Desulfotomaculum*-related sequences (Tables S1, S2). A relatively large proportion of HydA sequences (14.4–14.5%) from ST09 metagenomes shared high sequence similarity with those of paddy soil *Firmicutes*. Other HydA sequences within the *Firmicutes* (i.e., related to the orders *Halanaerobiales, Thermoanaerobacterales*, and *Selenomonadales*) were also observed but in less amounts (Figure 2). In addition, HydA sequences retrieved with high frequency were associated with those of *Spirochaetales* (11.9–18.3%), as well as *Deltaproteobacteria* (e.g., *Desulfobulbus* genus), *Bacteroidetes*, and *Thermotogales*, which are also known to be rich in fermentative H_2_-producers. The rest of metagenomic HydA sequences of PHF were associated with those of microbial mat inhabiting saline environments or oil fields (Boyd et al., 2009; Liu et al., 2015).

### [FeFe]-hydrogenase gene diversity in intertidal and submarine samples using PCR-amplified DNA sequencing analysis

Five samples of both intertidal and submarine Prony sites (“Bain des Japonais” fluids, BdJF; “Bain des Japonais” chimney, BdJC; “Rivière des Kaoris” fluids, RKF; “Rivière des Kaoris” chimney, RKC, and ST07 chimney) were tested for amplification of the large subunit of the [FeFe]-hydrogenase encoding gene (*hydA*) using three degenerate primer sets (FeFe-272F/FeFe-427R, HydH1f /HydH3r, hydF1/hydR1). PCR products of ~680 bp were obtained with the primer set hydF1/hydR1 from both the PHF samples and the control genomic DNA (i.e., *Desulfovibrio vulgaris* and *Clostridium saccharolyticum*). In contrast, no *hydA* gene amplification occurred for the PHF samples by using primer sets FeFe-272F/FeFe-427R or HydH1f/HydH3r, despite modifications of the PCR conditions (e.g., annealing temperature) with validation on controls (i.e., PCR products with the expected band size were observed at 50°C for control). Therefore, the primer sets hydF1/hydR1 was selected for further analysis (i.e., cloning and sequencing of *hydA* genes of PHF DNAs).

Five *hydA* gene libraries were analyzed to study the HydA diversity in the five different PHF samples and to gain insight into the HydA diversity of uncultivable H_2_-producers. A total of 181 *hydA* sequences were obtained from the five environmental samples BdJC (52), BdJF (61), RKC (28), RKF (26), and ST07 (14). Both the highest richness and the highest diversity were observed in BdJ samples (Table S3).

**Figure 4:**
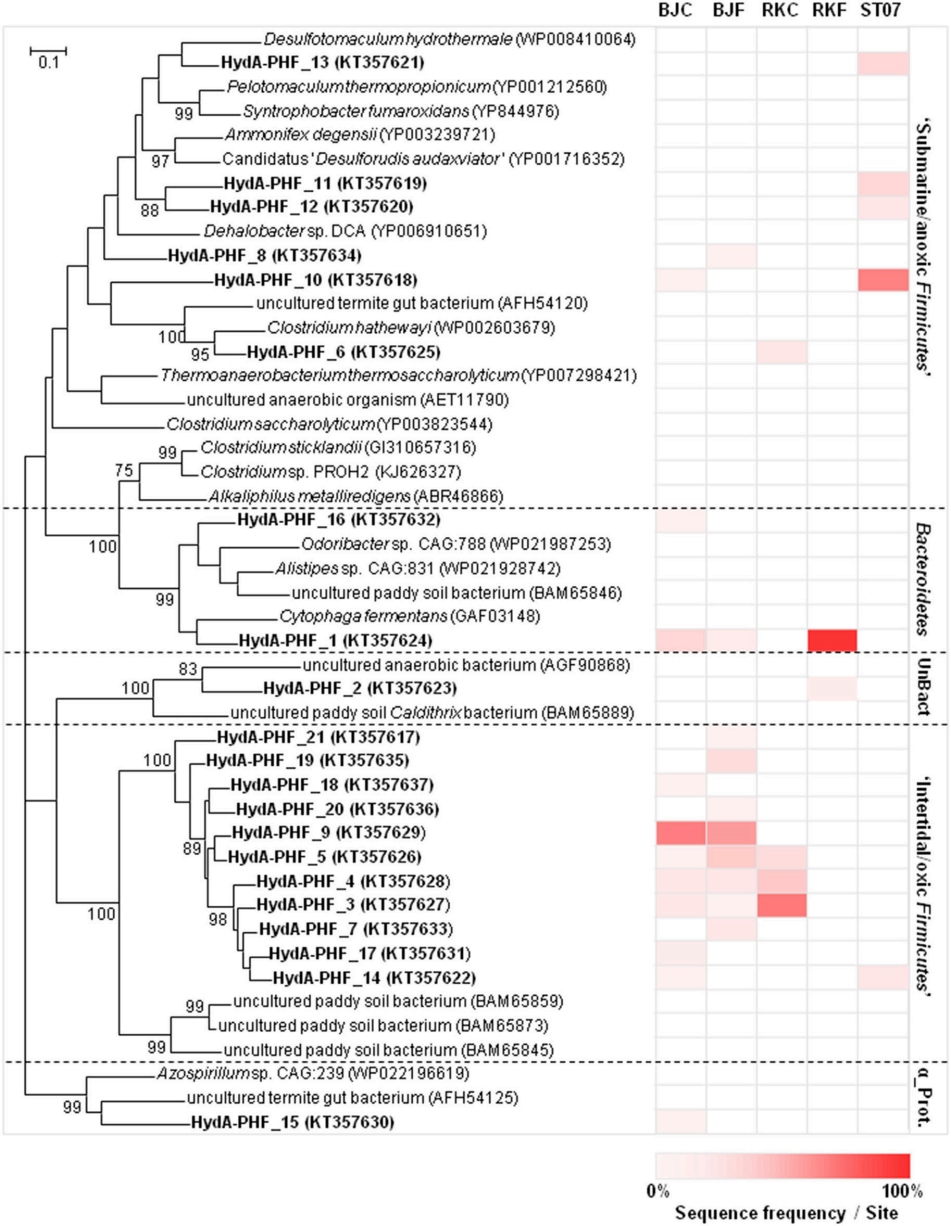
**Maximum-likelihood phylogenetic tree of [Fe-Fe]-hydrogenase sequences obtained from intertidal and submarine PHF sites**. Protein HydA sequences (188 amino acids) were obtained after translation of *hydA* gene sequences (obtained by cloning and Sanger sequencing) for 3 PHF hydrothermal sites: “Bain des Japonais” (BdJ), “Rivière des Kaoris” (RK), and “Aiguille de Prony” (ST07). The suffix “C” in sample names corresponds to “chimney” and the suffix “F” corresponds to “fluid”. The [FeFe]-hydrogenase sequences from PHF samples are marked in bold. Each OTU is represented by one representative sequence (at ≥80% similarity level). Percentage of OTU with respect to the total number of retrieved sequences in each PHF site is indicated by different levels of red from 0% (white) to 100% (red). Genbank accession numbers (in brackets) were obtained from the protein sequence database. Bootstrap values < 70% are not shown. The scale bar indicates 0.1% sequence divergence. UnBact and α_Prot stand for unclassified Bacteria and *Alphaproteobacteria*, respectively.

Figure 4 shows the phylogenetic relationship of the 21 HydA OTUs corresponding to the translated sequences of the *hydA* gene detected in the PHF samples. They were related to the *Firmicutes* (81.0% of the total sequences), followed by *Bacteroidetes* (9.5%), and *Alphaproteobacteria* phyla (4.8%). The bacterial community of the submarine chimney ST07 was mainly represented (92.9% of the total sequences) by mesophilic to thermophilic fermenters or sulfate-reducers related to *Firmicutes* (i.e., *Clostridiales* or *Thermoanaerobacterales*) isolated from terrestrial hot spring or subterrestrial environments, such as *Desulfotomaculum* spp. These putative “submarine/anoxic *Firmicutes”* HydA sequences were also mainly detected in the metagenomes of the submarine chimney ST09. In contrast, both BdJC and RKC samples were dominated by HydA sequences associated with those of uncultured *Firmicutes* retrieved from a paddy field soil (78.8 and 92.9% of the total sequences, respectively), which were less represented in the metagenomes of the submarine chimney ST09. BdJF sample revealed a similar proportion of HydA sequences (73.5% of the total sequences) associated with this cluster hence referred to here as “intertidal/oxic *Firmicutes* HydA group.” The HydA sequences retrieved from RKF were mainly associated to the *Bacteroidetes/Chlorobi* group (96.2%) and also affiliated with uncultured *Caldithrix*–like bacteria retrieved from paddy field soil or anaerobic sludge (3.9%).

### 16S rRNA gene diversity in intertidal and submarine PHF samples

In order to obtain accurate and consistent estimates of the overall bacterial diversity in the studied sites, 16S rRNA pyrosequencing were conducted on the same five PHF subsamples (BdJF and RKF fluids; BdJC, RKC, and ST07 chimneys) previously used for *hydA* PCR amplification and subsequent sequencing. After quality/size trimming and removal of chimeric sequences, pyrosequencing of bacterial 16S rRNA PCR products yielded a total of 20,495 sequences. These sequences were assigned to 969 OTUs (RKC: 161; RKF: 143; BDJC: 323; BDJF: 125; ST07: 217; Table S3). The PHF samples were dominated by 52 bacterial OTUs (Table 1). BdJC had the highest bacterial richness while ST07 sample displayed the highest bacterial diversity (Table S3).

**Table 1:** **Phylogenetic affiliation of the dominant bacterial OTUs obtained from 16S rRNA pyrosequencing analyses of Prony Hydrothermal Field (PHF) sites: “Bain des Japonais” (BdJ), “Rivière des Kaoris” (RK), and “Aiguille de Prony” (ST07)**.

**OTU IDs**	**Accession number**	Sequences per samples^*^ (%)					**Taxonomy (Phylum; Order)**	**Closest relatives retrieved from NCBI nucleotide database**			
		**BdJC**	**BdJF**	**RKC**	**RKF**	**ST07**		**Bacterial strain (Genbank accession number)**	**% Identity**	**Clone (Genbank accession number)**	**% Identity**
254218	KT344973	-	0.16	-	-	1.60	*Actinobacteria*; OPB41	*Olsenella profusa* (NR_116938)	88	clone dr84 (AY540822)	98
OTU332	KT344941	-	20.63	-	0.30	-	*Bacteroidetes*; *Bacteroidales*	*Natranoflexus pectinivorans* AP1 (NR_108635)	84	clone B257829_L43 (KP097103)^a^	98
OTU161	KT344980	-	0.16	-	-	2.38	*Chloroflexi*; *Dehalococcoidetes*	*Chloroflexi* SCGC AAA240-B13 (HQ675545)	85	clone PHF_13-B5_J02 (KJ149246)^b^	96
4347492	KT344946	-	-	18.05	-	-	*Cyanobacteria*; *Gloeobacterales*	*Synechococcus* sp. AECC1343 (EU729046)	97	clone PMB-63 (AB757744)	97
214987	KT344943	-	-	1.64	-	-	*Cyanobacteria*; *Pseudanabaenales*	*Pseudanabaena* sp. 1a-03 1a-03 (FR798944)	88	clone Flu2_7 (JF413310)	97
243177	KT344971	-	0.38	-	-	3.44	*Deinococcus*-*Thermus*; *Deinococcales*	*Truepera radiovictrix* RQ-24 (NR_074381)	89	clone St09-1-17 (KR911715)^b^	98
130884	KT344969	-	0.18	0.14	-	8.12	*Deinococcus*-*Thermus*; *Thermales*	*Meiothermus hypogaeus* AZM34c11 (NR_113226)	95	clone PHFST07_B5 (KF886174)^b^	97
OTU55	KT344982	0.53	1.21	-	-	1.25	*Firmicutes*; *Clostridiales*	*Caloranaerobacter azorensis* MV1087(NR_028919)	87	clone PHFST07_B9 (KF886154)^b^	96
545286	KT344939	-	9.15	-	-	6.83	*Firmicutes*. *Clostridiales*	*Clostridium septicum* H4 (KM975632)	89	clone PHFST07_B12 (KF886167)^b^	96
OTU1056	KT344978	-	0.21	-	-	2.61	*Firmicutes*; *Clostridiales*	*Desulfotomaculum* sp. ECP-C5 (AF529223)	87	clone PHF_2C-bac-D08 (KJ159198)^b^	92
244602	KT344972	-	0.01	-	-	2.31	*Firmicutes*; *Clostridiales*	*Dethiobacter alkaliphilus* AHT1 (NR_044205)	90	clone HPst091-1-1 (KM207235)^b^	98
OTU10	KT344977	-	-	-	-	1.48	*Firmicutes*; *Natranaerobiales*	*Natranaerobius trueperi* (NR_116280)	88	clone HPst091-1-1 (KM207235)^b^	97
237589	KT344970	-	0.12	-	-	1.27	*Firmicutes*; *Thermoanaerobacterales*	*Thermosediminibacter oceani* DSM 16646 (NR_074461)	86	clone PHF_2HY7-Ba-G08 (KJ159206)^b^	92
OTU1120	KT344940	-	1.94	-	-	4.36	Candidate division NPL-UPA2	*Pelotomaculum isophthalicicum* JI (NR_041320)	84	clone PHFST08_B2 (KF886073)^b^	92
815112	KT344976	0.55	0.10	-	-	3.34	*Alphaproteobacteria*; *Rhizobiales*	*Methyloceanibacter caenitepidi* Gela4 (AP014648)	98	clone 1FSeds_H08 (GQ412793)	98
OTU879	KT344984	0.07	-	-	-	1.01	*Alphaproteobacteria*; *Rhizobiales*	*Methyloceanibacter caenitepidi* Gela4 (AP014648)	96	clone PHF_13-B3_F02 (KJ149247)^b^	97
550276	KT344975	-	-	-	-	1.06	*Alphaproteobacteria*; *Rhizobiales*	*Hyphomicrobium* sp. Ellin112 (AF408954)	95	clone GM-BSS-cloneDB12 (AB453748)	97
745987	KT344935	59.83	0.19	-	-	0.28	*Alphaproteobacteria*; *Rhodobacterales*	*Rhodobaca bogoriensis* SLB (EU908048)	98	clone HL7711_P4E7 (KJ004401)	98
OTU431	KT344936	1.60	0.01	-	-	-	*Alphaproteobacteria*; *Rhodobacterales*	*Rhodobaca bogoriensis* SLB (EU908048)	97	clone HL7711_P4E7 (KJ004401)	97
OTU890	KT344952	-	-	8.56	-	-	*Alphaproteobacteria*; *Rhodospirillales*	*Paracraurococcus* sp. 1PNM-27 (JQ608332)	95	clone B1203_GOR34 (KP097454)^a^	96
1082059	KT344933	1.79	-	0.39	-	-	*Alphaproteobacteria*; *Sphingomonadales*	*Erythrobacter* sp. A5(1) (KP265725)	98	clone MAY3C10 (KF179645)	98
[0,-197]xpink206pt10pt838837	KT344950	-	-	5.10	0.06	-	*Betaproteobacteria*; *Burkholderiales*	*Hydrogenophaga* sp. Chr-40 (JQ863382)	98	clone B93726_L43 (KP097382)^a^	99
796555	KT344949	0.48	0.01	5.64	-	-	*Betaproteobacteria*; *Burkholderiales*	*Hydrogenophaga* sp. TR7-01(AB166886)	98	clone B3025389_L43 (KP097124)^a^	99
3025389	KT344945	0.34	0.01	3.75	-	0.02	*Betaproteobacteria*; *Burkholderiales*	*Hydrogenophaga* sp. TR7-01(AB166886)	98	clone B3025389_L43 (KP097124)^a^	99
647775	KT344934	1.79	0.18	32.73	15.53	0.87	*Betaproteobacteria*; *Burkholderiales*	*Hydrogenophaga* sp. TR7-01(AB166886)	98	clone B3025389_L43 (KP097124)^a^	99
261198	KT344944	0.02	-	2.40	0.89	-	*Betaproteobacteria*; *Burkholderiales*	*Hydrogenophaga* sp. Chr-40 (JQ863382)	98	clone B93726_L43 (KP097382)^a^	99
4430221	KT344947	0.02	-	1.13	0.08	-	*Betaproteobacteria*; *Burkholderiales*	*Hydrogenophaga* sp. TR7-01(AB166886)	98	clone B3025389_L43 (KP097124)^a^	98
572939	KT344957	-	-	0.02	4.99	0.42	*Betaproteobacteria*; *Burkholderiales*	*Hydrogenophaga* sp. Chr-40 (JQ863382)	93	clone B93726_L43 (KP097382)^a^	99
546165	KT344974	0.05	0.01	0.92	0.47	1.11	*Betaproteobacteria*; *Burkholderiales*	*Hydrogenophaga* sp. TR7-01(AB166886)	98	clone B3025389_L43 (KP097124)^a^	99
OTU300	KT344981	-	-	0.31	0.55	2.94	*Betaproteobacteria*; *Burkholderiales*	“*Serpentinomonas”* sp. B1 (AP014569)	98	clone B572939_L43 (KP097286)^a^	99
OTU1176	KT344951	0.02	-	1.15	-	-	*Betaproteobacteria*; *Burkholderiales*	*Hydrogenophaga* sp. TR7-01 (AB166886)	91	clone B3025389_L43 (KP097124)^a^	98
OTU760	KT344983	-	-	-	-	16.65	*Deltaproteobacteria*; *Desulfobacterales*	*Desulfurivibrio alkaliphilus* AHT2 (NR_074971)	93	clone PHFST07_B11 (KF886157)^b^	92
1126915	KT344937	-	7.44	-	-	3.01	*Deltaproteobacteria*; *Desulfovibrionales*	*Desulfonatronum cooperativum* Z-7999 (NR_043143)	98	clone PHFBdJ_B8 (KF886124)^b^	92
129416	KT344938	-	12.81	-	-	1.18	*Deltaproteobacteria*; *Desulfovibrionales*	*Desulfonatronum cooperativum* Z-7999 (NR_043143)	97	clone PHFST07_B3 (KF886171)^b^	92
OTU370	KT344942	0.02	36.55	-	-	2.24	*Deltaproteobacteria*; *Desulfovibrionales*	*Desulfonatronum cooperativum* Z-7999 (NR_043143)	98	clone PHFBdJ_B8 (KF886124)^b^	92
823476	KT344962	-	-	-	3.63	-	*Gammaproteobacteria*; *Alteromonadales*	*Alteromonas* sp. DSSK2-12 (KR094792)	92	clone 12S_128 (KP183024)	92
899488	KT344964	-	-	-	2.97	-	*Gammaproteobacteria*; Alteromonadales	*Alteromonas* sp. DSSK2-12 (KR094792)	92	clone 12S_128 (KP183024)	92
OTU159	KT344979	-	-	-	-	1.46	*Gammaproteobacteria*; *Methylococcales*	*Methylomonas* sp. R-49799 (HG970730)	92	clone B94840_L43 (KP097385)^a^	96
939892	KT344965	-	-	-	1.14	-	*Gammaproteobacteria*; *Oceanospirillales*	*Halomonas boliviensis* TB-129 (KF817741)	91	clone K_87 (KF783323)	95
439982	KT344955	-	-	-	1.39	-	*Gammaproteobacteria*; *Pseudomonadales*	*Acinetobacter* sp. CIP 102637 (JQ638581)	92	clone K323G02 (GU256408)	97
710275	KT344959	-	-	-	1.16	-	*Gammaproteobacteria*; *Pseudomonadales*	*Acinetobacter ursingii* NBRC 110605 (LC014147)	92	clone K323G02 (GU256408)	97
OTU476	KT344968	-	-	-	1.66	-	*Gammaproteobacteria*; *Pseudomonadales*	*Acinetobacter baumannii* GRI-SD-LC1 (KR132555)	93	clone K323G02 (GU256408)	98
405425	KT344954	-	-	-	-	-	*Gammaproteobacteria*; *Pseudomonadales*	*Acinetobacter* sp. 140D (KM021154)	92	clone K323G02 (GU256408)	96
543942	KT344956	-	-	-	4.96	-	*Gammaproteobacteria*; *Pseudomonadales*	*Acinetobacter* sp. 140D (KM021154)	92	clone K323G02 (GU256408)	96
573124	KT344958	-	-	-	4.08	-	*Gammaproteobacteria*; *Pseudomonadales*	*Acinetobacter* sp. 140D (KM021154)	92	clone K323G02 (GU256408)	96
OTU106	KT344967	-	-	-	6.04	-	*Gammaproteobacteria*; *Pseudomonadales*	*Acinetobacter johnsonii* 2P2D5 (HF937031)	92	clone K323G02 (GU256408)	96
1107335	KT344953	-	-	-	6.18	-	*Gammaproteobacteria*; *Pseudomonadales*	*Acinetobacter calcoaceticus* SYJ1-3 (KR262850)	92	clone K323G02 (GU256408)	97
780555	KT344960	0.07	0.09	-	1.91	0.26	*Gammaproteobacteria*; *Pseudomonadales*	*Pseudomonas* sp. SRP1497 (KP452755)	91	clone B4316720_L43 (KP097170)^a^	99
829851	KT344963	0.69	0.07	0.02	13.28	0.05	*Gammaproteobacteria*; *Pseudomonadales*	*Pseudomonas* sp. SRP1497 (KP452755)	91	clone B4316720_L43 (KP097170)^a^	99
818602	KT344961	0.32	0.04	-	3.74	0.05	*Gammaproteobacteria*; *Pseudomonadales*	*Pseudomonas* sp. SRP1497 (KP452755)	91	clone B4316720_L43 (KP097170)^a^	99
578490	KT344948	0.30	-	4.21	1.14	-	*Gammaproteobacteria*; *Xanthomonadales*	*Silanimonas* sp. JK13 (KF206369)	92	clone B578490_L43 (KP097289)^a^	99
967275	KT344966	-	-	-	2.00	-	*Gammaproteobacteria*; *Xanthomonadales*	Strain SCGC AAA044-J23 (HQ663492)	92	clone Mineral.top.6.4_426600 (LN540678)	92

* *Suffixes “C” and “F” in sample names stand respectively for chimney and fluid*.

a *Environmental sequences obtained in a previous study from serpentinite-hosted sources of Voltri Massif (Italy; Quéméneur et al., 2015)*.

b *Environmental sequences obtained in previous studies of the Prony Hydrothermal field (New Caledonia; Quéméneur et al., 2014; Postec et al., 2015)*.

*Different colors indicate different bacterial taxa (classes or phyla)*.

**Figure 5:**
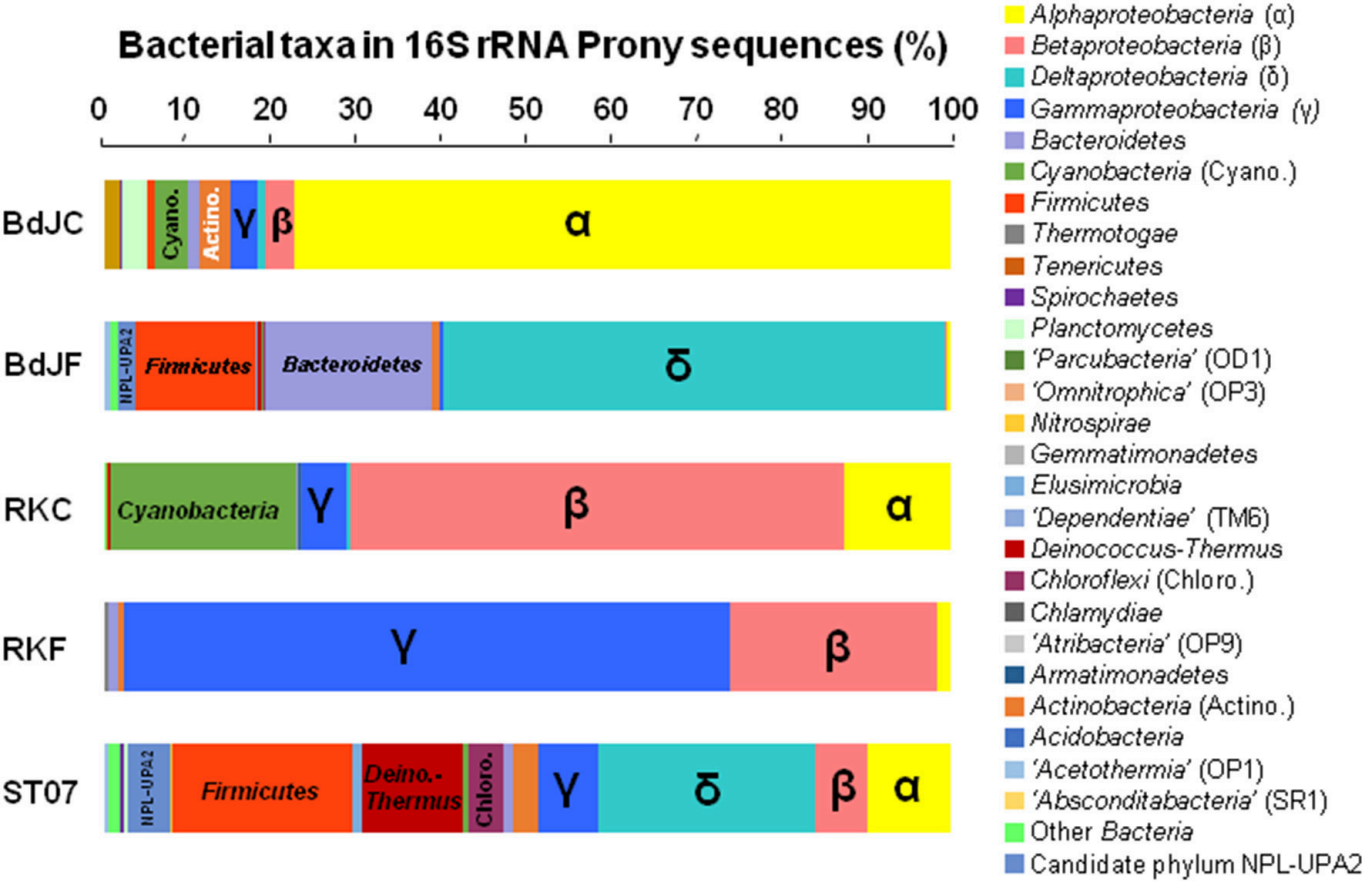
**Distribution and taxonomic assignments of 16S rRNA bacterial sequences in chimneys and fluids of interdidal and submarine sites of PHF**. Relative phylogenetic abundance was based on frequencies of the 16S rRNA gene sequences (with respect to the total number of sequences obtained by pyrosequencing) affiliated to major phylogenetic phyla or class in the bacterial communities of the “Bain des Japonais” (BdJ), “Rivière des Kaoris” (RK), and the “Aiguille de Prony” (ST07). The suffix “C” in sample names corresponds to “chimney” and the suffix “F” corresponds to “fluid.” The abbreviations of phyla and the former names of candidate phyla are indicated in parentheses.

The global structure of the bacterial communities obtained for each sample is shown in Figure 5. The total bacterial OTUs were assigned to 24 phyla and candidate phyla (including candidate phylum NPL-UPA2), and other unclassified groups (Figure 5). *Bacteroidetes, Cyanobacteria, Firmicutes, Proteobacteria*, and *Thermus-Deinococcus* accounted for the five main phyla (>10% of the total sequences each in average), representing 93.5% of the total bacterial communities.

BdJF and ST07 displayed the highest occurrence of *Firmicutes* and *Deltaproteobacteria* (Figure 5). *Firmicutes* were mainly represented by fermentative heterotrophs belonging to *Clostridiales, Natranaerobiales* (only detected in ST07), and *Thermoanaerobacterales* (Table 1). BdJF displayed the highest occurrence of *Deltaproteobacteria* sequences, which were exclusively affiliated to the alkaliphilic *Desulfonatronum* genus, while ST07 chimney sample comprised two alkaliphilic deltaproteobacterial groups: the first and most abundant related to the genus *Desulfurivibrio*, and the second related to the genus *Desulfonatronum*. BdJF bacterial community contained a large part (14.3%) of anaerobic, haloalkaliphilic, hydrolytic, and fermenting members of the *Natronoflexus* genus (*Bacteroidetes* phylum).

Remarkably, on the contrary to BdJF and ST07, the BdJC sample was dominated by *Alphaproteobacteria*, mainly represented by the anoxygenic phototrophic *Rhodobaca* genus. Moreover, photosynthetic *Cyanobacteria* were abundantly detected in RKC sample (21.9% of the bacterial community), while RKF bacteria were largely dominated by *Gammaproteobacteria*. Finally, H_2_-consuming *Betaproteobacteria* (i.e., *Hydrogenophaga*/“*Serpentinomonas*” members) were detected in all samples, especially in RKC and RKF, where they represented 58.4 and 24.4% of the total bacterial communities, respectively.

### Cultures and isolation of alkaliphilic hydrogen-producing *Firmicutes* from PHF

**Figure 6:**
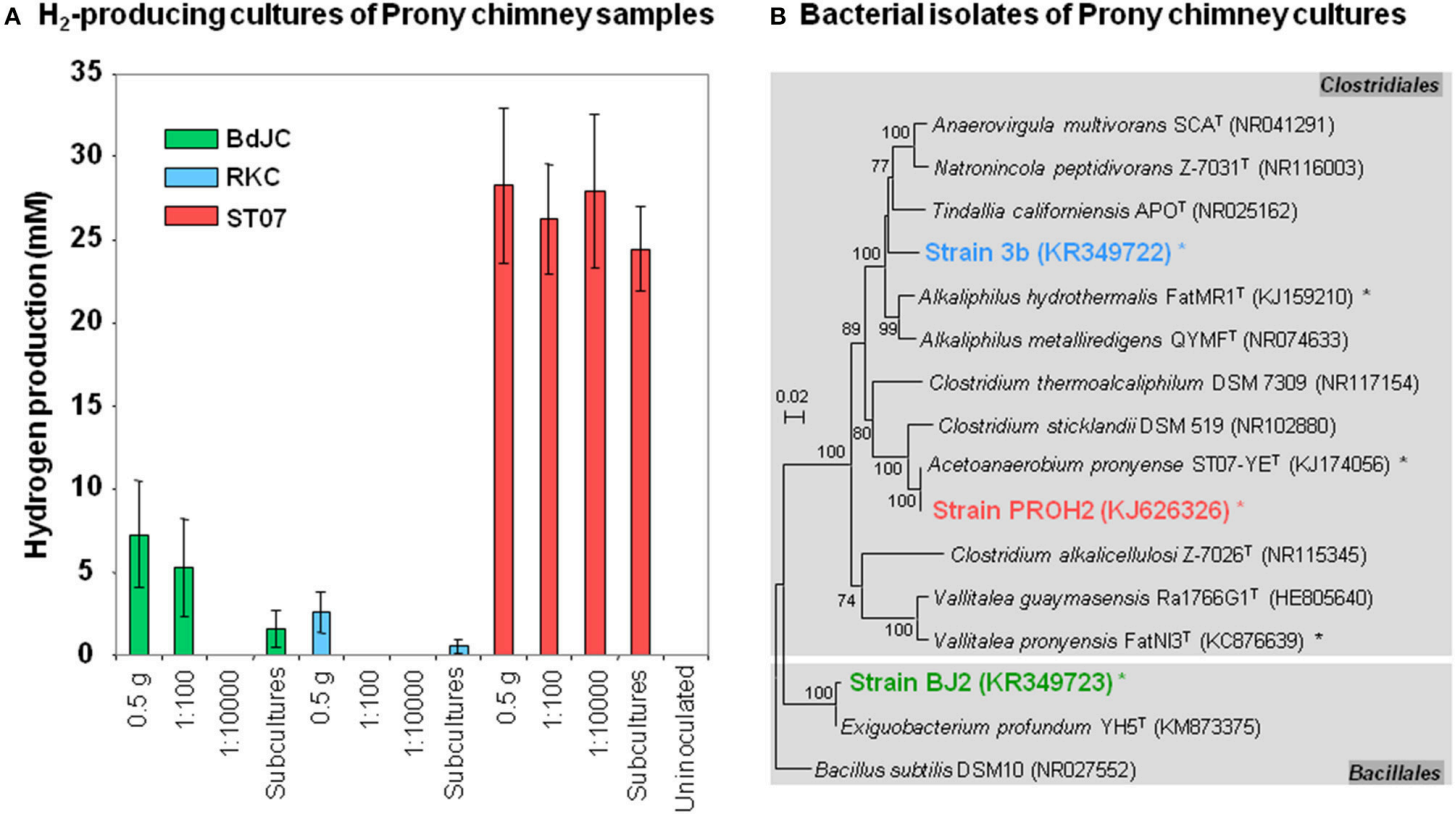
**Hydrogen-producing cultures obtained from hydrothermal chimneys of the Prony bay: performances and identification of bacterial isolates**. **(A)** Hydrogen-producing cultures were attempted using several dilutions of chimney subsamples from three Prony sites: “Bain des Japonais” (BdJ), “Rivière des Kaoris” (RK), and the “Aiguille de Prony” (ST07). The suffix “C” in sample names stands for “chimney.” Subcultures were also performed from the first round of positive enrichment cultures. Values are mean of biological replicates ± standard deviation (errors bars). **(B)** Maximum-likelihood tree based on 16S rRNA gene sequences showing the phylogenetic position of *Firmicutes* strains 3b, PROH2, and BJ2, isolated from hydrogen-producing cultures of Prony hydrothermal Field (PHF). Representative sequences in the tree were obtained from GenBank (accession number in the brackets). Other bacterial species isolated from PHF are indicated by asterisks. Bootstrap values >70% are indicated at nodes.

Two subsamples of the five PHF samples (BdJC, BdJF, RKC, RKF, and ST07) were cultivated into BYG medium at an initial pH of 9.5 in order to enrich for fermentative alkaliphilic H_2_-producing bacteria. In total, 70 tubes were inoculated (5 samples × 7 dilutions × 2 replicates). After 1 week of incubation at 37°C, H_2_ production was detected for all tested chimney samples (i.e., RKC, BdJC, and ST07; Figure 6A). The H_2_ production was accompanied by a decrease in both glucose concentration and pH (final pH 8.5 ± 0.5), as a result of acidic metabolite production (i.e., acetate and butyrate). No H_2_ production was observed in controls and enrichment cultures from fluids (BdJF and RKF) even after 1 month of incubation under the same culture conditions (37°C, pH 9.5). Moreover, no H_2_ production was observed after 1 month of incubation at 55°C with similar pH and substrate conditions from any tested samples (chimney samples and fluids). This result suggests that no thermo-alkaliphilic fermentative microorganisms were involved in production of H_2_ in PHF with these substrates.

The RKC culture and the lowest dilution of BdJC (10^−2^) and ST07 (10^−2^) cultures yielding a significant H_2_ production were transferred into a fresh culture medium after 2 weeks of incubation. The highest H_2_ production (24.5 ± 2.5 mM) was reached in ST07 subcultures (Figure 6A), while extremely low H_2_ production (< 1.5 mM) was observed in both BdJC and RKC subcultures (after 7 days of incubation). All H_2_-producing subcultures (BdJC, RKC, and ST07) displayed before the isolation procedure, a weak bacterial richness with the detection of only one dominant OTU belonging to *Firmicutes* after 16S rRNA-based cloning/sequencing analyses (data not shown). These dominant OTUs corresponded to the bacterial strains finally isolated by the roll-tube method from each H_2_-producing subcultures. As shown in Figure 6B, the alkaliphilic, highly efficient H_2_-producing ST07 culture was dominated by the strain PROH2 (sharing 99.9 and 96.8% 16S rRNA identity with *Acetoanaerobium pronyense* and *Clostridium sticklandii*, respectively; Mei et al., 2014; Bes et al., 2015). The RKC cultures were dominated by the alkaliphilic strain 3b having *Alkaliphilus hydrothermalis* as its closest phylogenetic relative (92.3% 16S rRNA identity), hence representing a novel species of a new genus in the order *Clostridiales*, for which the name “*Serpentinicella alkaliphila*” has been recently proposed (Mei et al., 2016). The BdJC cultures were dominated by strain BJ2 closely related to *Exiguobacterium profundum* (99.5% 16S rRNA identity) a facultative anaerobe originating from a deep-sea hydrothermal vent and belonging to the order *Bacillales*.

## Discussion

### Changes in [FeFe]-hydrogenase diversity between intertidal and submarine prony sites

Bacterial populations harboring *hydA* genes in both intertidal and submarine PHF samples were clearly dominated by *Firmicutes*, as previously observed in two other serpentinite-hosted ecosystems [i.e., Lost City chimneys, discharging hot fluid (90°C) with high pH (10.8), and H_2_ (13 mmol/kg) and anoxic Tablelands-WHC2b fluid, pH 12.1, Eh −733 mV, 0.24 mM-H_2_; Brazelton et al., 2012; Figure 4]. However, different *hydA* patterns were observed depending on the nature and physico-chemical characteristics of the PHF samples (e.g., fluid vs. carbonate concretions/chimneys, or water depth), in agreement with previous 16S rRNA gene-based molecular studies on this hydrothermal field (Quéméneur et al., 2014; Postec et al., 2015; this study). PHF *hydA* populations were mainly divided in two groups. One is the “submarine/anoxic HydA group,” related to strictly anaerobic *Firmicutes* (e.g., *Clostridium* and *Desulfotomaculum* genera) recovered from deep environments (Haouari et al., 2008; Aüllo et al., 2013). This is the case of submarine ST07 and ST09 chimneys, as well as fluid end-members of BdJ site [BdJF, pH 10.8, Eh −352 mV, 6.4 mM-H_2(*g*)_] and RK site [RKF, pH 10.9, Eh −195, 13.4 mM-H_2(*g*)_] (data from Monnin et al., 2014) characterized by pH close to 11 and low redox potentiel. In contrast, an “intertidal/oxic HydA group,” affiliated to *Firmicutes* HydA sequences from paddy field soil (Japan; Baba et al., 2014), was predominant in the intertidal BdJ and RK chimneys. Irrigated paddy field soils are characterized by alternating aerobic and anaerobic conditions, when they are drained and flooded during rice cultivation periods (Lüdemann et al., 2000). Similar fluctuating exposure/concentration of oxygen may exist in both intertidal BdJ and RK chimneys, which are alternatively uncovered or covered by seawater at low or high tide, respectively (Monnin et al., 2014; Quéméneur et al., 2014). However, *hydA* genes are commonly detected abundantly in anoxic zones, but not in other intertidal/oxic locations (e.g., Tablelands-TLE and Great Salt Lake; Brazelton et al., 2012; Boyd et al., 2014). Therefore, this new “intertidal/oxic HydA group” distantly related to HydA sequences from cultivated microorganisms related to uncultured *Firmicutes* from paddy soils and may hence represent a new class of unknown [Fe-Fe]-hydrogenases of new aero-tolerant or microaerophilic microorganisms to be discovered (Figure 4).

### Abundant [FeFe]-hydrogenases of *Desulfotomaculum* spp. in PHF metagenomes

Numerous HydA OTUs obtained from submarine PHF sites by using both metagenomic and PCR-amplified DNA sequencing analyses were closely related to sulfate-reducing *Firmicutes* of the *Desulfotomaculum* genus. This finding is consistent with a previous metagenomic investigation of [Fe-Fe]-hydrogenases in the serpentinization-driven LCHF (Brazelton et al., 2012). *Desulfotomaculum* spp. are well-adapted to colonize deep submarine environments where they are nonetheless better known to consume H_2_ for growth through sulfate reduction (Aüllo et al., 2013) rather than to produce H_2_ by fermentation. However, some *Desulfotomaculum* species possess the ability to grow in syntrophy with hydrogenotrophic methanogens (to which they transfer H_2_ they produce) and have even lost their ability to reduce sulfate in anoxic systems (Imachi et al., 2006), when the concentration of sulfate is quite low, as it was measured in PHF end member fluids (Monnin et al., 2014; Quéméneur et al., 2014). Some [FeFe]-hydrogenases of other sulfate-reducers (e.g., *Desulfovibrio* spp.) may also be bifunctional, and depending on the environmental conditions, they may produce H_2_ in synthrophic conditions, instead of catalyzing H_2_ oxidation (Meyer et al., 2013). In PHF chimney samples, no *Desulfotomaculum* species was previously detected by sequence analyses of *dsrB* genes, used as molecular marker of sulfate-reducing bacteria (Quéméneur et al., 2014; Postec et al., 2015) in contrast to the LCHF where they were found to be abundant (Brazelton et al., 2006; Gerasimchuk et al., 2010). Several attempts to cultivate sulfate-reducing bacteria, as well as anaerobic hydrogenotrophs, from these submarine serpentinite-hosted environments were unsuccessful for both locations (Postec et al., 2015), despite their abundance in both 16S rRNA and *dsrB* gene databases (e.g., *Desulfotomaculum* spp. for LCHF and *Desulfonatronum* spp. for PHF). However, it is well-known that the use of H_2_ as electron donor is generally considered as more thermodynamically favorable than use of organic acids or sugars as electron donor (Thauer et al., 1977; Amend et al., 2011). This probably means that such sulfate-reducing bacteria have a particular metabolism (and/or a synthrophic life style), and consequently may possess a novel type of hydrogenases. Altogether, these results indicate that *Desulfotomaculum*–related [Fe-Fe]-hydrogenases certainly play a crucial role in the biological H_2_ cycle in serpentinite-hosted environments but at this stage of knowledge it is difficult to stand if these [Fe-Fe]-hydrogenases were involved mostly in H_2_ production or consumption or both depending on environmental conditions.

### High [FeFe]-hydrogenase diversity in a hyperalkaline and mesothermic environment

An unexpectedly large HydA diversity, related to phyla that include *Alphaproteobacteria, Bacteroidetes*, and *Firmicutes*, was observed in the alkaline serpentinite-hosted PHF, where high amounts of H_2_, likely of mixed origin (geological and biological), is produced. This finding strongly contrasts with that reported by Boyd et al. (2010) who also used HydA sequences to study the diversity of H_2_-producing bacteria in basalt-hosted hydrothermal springs of the Yellowstone National Park (YNP). Indeed, they demonstrated that the HydA diversity in their samples was strongly constrained by pH (with the lowest diversity being found in springs with high pH of 9–10) and was mainly represented by uncultivated members of the *Elusimicrobia* phylum (known as “Termite Group 1”) at high pH. Although the geological, mineralogical and chemical setting differs in PHF, we show that neither pH solely nor in combination with *in situ* H_2_ concentration can explain the HydA diversity in the PHF system, and that alkaline habitats can also harbor a wide range of potential H_2_-producers, comparable to previous observations in habitats with acidic or neutral pH conditions (Xing et al., 2008; Schmidt et al., 2010). This sharp difference with what was observed in YNP hot springs may be explained by the combination of two strong environmental stresses (i.e., high pH coupled with high temperature) that seems to dramatically decrease the potential of biological H_2_ production. In our study, no H_2_ production was detected in enrichments carried out at pH 9.5 and temperature exceeding 55°C and only low proportions of the *hydA* sequences retrieved from PHF metagenomes could be affiliated to thermophilic H_2_ producers (such as *Thermotogales*), in agreement with results obtained from YNP springs, where *hydA* genes were undetected at alkaline pH and temperature above 65°C (Boyd et al., 2010).

### Alkaliphilic and fermentative hydrogen-producing *Firmicutes*

Whatever the approach used, it appears that the order *Clostridiales*, phylum *Firmicutes*, contained the highest number of *hydA* genes and thus potential H_2_-producing candidates in PHF samples. Our molecular data are in agreement with recent extensive genomic studies, showing a predominance of *hydA* genes in *Firmicutes* genomes (Peters et al., 2015; Poudel et al., 2016). Among them, the majority of the *hydA Firmicutes* are related to the “G1 Hyd group,” which mainly contains representatives of H_2_-producing [FeFe]-hydrogenases (Poudel et al., 2016). *Clostridiales* were also the most frequently cultivated bacteria from PHF chimneys, allowing us to isolate numerous strains, some of which being already described as new species (e.g., *Alkaliphilus hydrothermalis, Acetoanaerobium pronyense, Vallitalea pronyensis*; Ben Aissa et al., 2014, 2015; Bes et al., 2015). These *Clostridiales* can be involved in H_2_ production by fermenting a wide range of organic compounds as substrates (e.g., sugars, proteins, individual amino acids, carboxylic acids), which could originate from the decay of primary microbial colonizers of such alkaline environments. Indeed, a high biomass has been previously detected in PHF chimneys with population ranging from 1 to 6 × 10^7^ bacterial cells per gram of chimneys (Quéméneur et al., 2014). Additionally, the hydrothermal degradation at depth of serpentinite hosted ecosystems has been shown to lead to the production of organic acids circulating throughout the hydrothermal system (Pasini et al., 2013). However, further studies on fermentative H_2_ producers occupying these alkaline habitats are needed to ascertain their geomicrobiological role to be played in serpentinite-hosted ecosystems and to assess to what extent they contribute to the H_2_ budget. Most of the studies on fermentative H_2_ production were conducted under acidic or neutral pH conditions (optimal pH ranging from 5 to 6; Xing et al., 2008; Wang and Wan, 2009; Quéméneur et al., 2011), and only two alkaliphilic H_2_-producers have been isolated from alkaline environments so far (Begemann et al., 2012; Mei et al., 2014). Nonetheless, fermentative H_2_ production have been not only described as thermodynamically more attractive under alkaline conditions (at ambient temperature), but also reported to be enhanced and stabilized at high initial pH (Cai et al., 2004; Xiao and Liu, 2006). Besides, the alkaliphilic anaerobe, *Clostridium* sp. PROH2, isolated from the “Aiguille de Prony” (ST07 chimney), demonstrated efficient H_2_ production with H_2_ yields similar to that of other neutrophilic and mesothermic clostridial species studied so far. This clostridial strain was able to produce 2.71 moles of H_2_ per mole of glucose at high pH (9.5), low salinity and moderate temperature (37°C; Mei et al., 2014). Such pure cultures of extremophilic microorganisms constitute interesting biotechnological alternatives for producing H_2_ with high efficiency from vegetal biomass and organic wastes in non-sterile systems since the high-pH conditions efficiently prevent growth of most contaminants that prevail under neutrophilic conditions.

## Conclusion

This study revealed an unexpected high diversity of [FeFe]-hydrogenase genes mostly related to *Firmicutes* in the hyperalkaline and serpentinite-hosted PHF. Such a high diversity may reflect either a high metabolic capability at the community level, with various fermenting bacteria occupying distinct micro-habitats in the porous structures of the carbonate chimneys and in the fluids, or individual metabolic flexibility of these indigenous microorganisms adapted to the various stresses they have to face due to harsh and fluctuating environmental conditions (e.g., Eh, pH, O_2_, nutrient deprivation) as recently evidenced by Pisapia et al. (in press). Their novelty can be explained by the unique feature of the serpentinite-hosted PHF, which discharges low-temperature (< 40°C) and low-salinity fluids in a shallow submarine environment. Clearly, further investigations are mandatory to assess the *in situ* functioning and directionality (i.e., reverse or forward) of these new and diverse [FeFe]-hydrogenases associated to members of the order *Clostridiales*. They also need to be complemented by isotopic investigations aiming at determining the ratio of biotic to abiotic H_2_ produced at the PHF, and field measurements to *in situ* assess the microbiological H_2_ production. As elevated concentrations of N_2_ were also reported in the fluids discharged at the PHF (Monnin et al., 2014; Deville and Prinzhofer, 2016), the involvement of serpentinite-hosted microbial communities in the deep nitrogen cycle and in the overall production of N_2_ will be another pending question to tackle within the next future.

## Author contributions

All authors listed, have made substantial, direct and intellectual contribution to the work, and approved it for publication.

### Conflict of interest statement

The authors declare that the research was conducted in the absence of any commercial or financial relationships that could be construed as a potential conflict of interest.

## References

[B1] Amend J. P. McCollom T. M. Hentscher M. Bach W. (2011). Catabolic and anabolic energy for chemolithoautotrophs in deep-sea hydrothermal systems hosted in different rock types. Geochim. Cosmochim. Acta 75, 5736–5748. 10.1016/j.gca.2011.07.041

[B2] Aüllo T. Ranchou-Peyruse A. Ollivier B. Magot M. (2013). *Desulfotomaculum* spp. and related gram-positive sulfate-reducing bacteria in deep subsurface environments. Front. Microbiol. 4:362 10.3389/fmicb.2013.00362 PMC3844878 24348471

[B3] Baba R. Kimura M. Asakawa S. Watanabe T. (2014). Analysis of [FeFe]-hydrogenase genes for the elucidation of a hydrogen-producing bacterial community in paddy field soil. FEMS Microbiol. Lett. 350, 249–256. 10.1111/1574-6968.12335 24261851

[B4] Balch W. E. Fox G. E. Magrum L. J. Woese C. R. Wolfe R. S. (1979). Methanogens: reevaluation of a unique biological group. Microbiol. Rev. 43, 260–296. 390357 10.1128/mr.43.2.260-296.1979 PMC281474

[B5] Begemann M. B. Mormile M. R. Sitton O. C. Wall J. D. Elias D. A. (2012). A streamlined strategy for biohydrogen production with *Halanaerobium hydrogeniformans*, an alkaliphilic bacterium. Front. Microbiol. 3:93. 10.3389/fmicb.2012.00093 22509174 PMC3325762

[B6] Ben Aissa F. Postec A. Erauso G. Payri C. Pelletier B. Hamdi M.. (2014). *Vallitalea pronyensis* sp. nov., isolated from a marine alkaline hydrothermal chimney. Int. J. Syst. Evol. Microbiol. 64, 1160–1165. 10.1099/ijs.0.055756-0 24408522

[B7] Ben Aissa F. Postec A. Erauso G. Payri C. Pelletier B. Hamdi M.. (2015). Characterization of *Alkaliphilus hydrothermalis* strain FatMR1 sp. nov., a novel alkaliphilic anaerobic bacterium, isolated from a carbonaceous chimney of the Prony Hydrothermal Field, New Caledonia. Extremophiles 19, 183–188. 10.1007/s00792-014-0697-y 25319677

[B8] Bes M. Merrouch M. Joseph M. Quéméneur M. Payri C. Pelletier B.. (2015). Acetoanaerobium pronyense sp. nov., an anaerobic mesophilic bacterium isolated from the Prony alkaline Hydrothermal Field, New Caledonia. Int. J. Syst. Evol. Microbiol. 65, 2574–2580. 10.1099/ijs.0.000307 25948619

[B9] Boyd E. S. Hamilton T. L. Spear J. R. Lavin M. Peters J. W. (2010). [FeFe]-hydrogenase in Yellowstone National Park: evidence for dispersal limitation and phylogenetic niche conservatism. ISME J. 4, 1485–1495. 10.1038/ismej.2010.76 20535223

[B10] Boyd E. S. Hamilton T. L. Swanson K. D. Howells A. E. Baxter B. K. Meuser J. E.. (2014). [FeFe]-hydrogenase abundance and diversity along a vertical redox gradient in Great Salt Lake, USA. Int. J. Mol. Sci. 15, 21947–21966. 10.3390/ijms151221947 25464382 PMC4284687

[B11] Boyd E. S. Spear J. R. Peters J. W. (2009). [FeFe]-hydrogenase genetic diversity provides insight into molecular adaptation in a saline microbial mat community. Appl. Environ. Microbiol. 75, 4620–4623. 10.1128/AEM.00582-09 19429563 PMC2704818

[B12] Brazelton W. J. Morrill P. L. Szponar N. Schrenk M. O. (2013). Bacterial communities associated with subsurface geochemical processes in continental serpentinite springs. Appl. Environ. Microbiol. 79, 3906–3916. 10.1128/AEM.00330-13 23584766 PMC3697581

[B13] Brazelton W. J. Nelson B. Schrenk M. O. (2012). Metagenomic evidence for H_2_ oxidation and H_2_ production by serpentinite-hosted subsurface microbial communities. Front. Microbiol. 2:268 10.3389/fmicb.2011.00268 PMC3252642 22232619

[B14] Brazelton W. J. Schrenk M. O. Kelley D. S. Baross J. A. (2006). Methane-and sulfur-metabolizing microbial communities dominate the Lost City hydrothermal field ecosystem. Appl. Environ. Microbiol. 72, 6257–6270. 10.1128/AEM.00574-06 16957253 PMC1563643

[B15] Cai M. Liu J. Wei Y. (2004). Enhanced biohydrogen production from sewage sludge with alkaline pretreatment. Environ. Sci. Technol. 38, 3195–3202. 10.1021/es0349204 15224755

[B16] Caporaso J. G. Kuczynski J. Stombaugh J. Bittinger K. Bushman F. D. Costello E. K.. (2010). QIIME allows analysis of high-throughput community sequencing data. Nat. Methods 7, 335–336. 10.1038/nmeth.f.303 20383131 PMC3156573

[B17] Chao A. (1984). Nonparametric estimation of the number of classes in a population. Scand. J. Stat. 11, 265–270.

[B18] Deville E. Prinzhofer A. (2016). The origin of N_2_-H_2_-CH_4_-rich natural gas seepages in ophiolitic context: a major and noble gases study of fluid seepages in New Caledonia. Chem. Geol. 440, 139–147. 10.1016/j.chemgeo.2016.06.011

[B19] Dowd S. E. Callaway T. R. Wolcott R. D. Sun Y. McKeehan T. Hagevoort R. G.. (2008). Evaluation of the bacterial diversity in the feces of cattle using 16S rDNA bacterial tag-encoded FLX amplicon pyrosequencing (bTEFAP). BMC Microbiol. 8:125. 10.1186/1471-2180-8-125 18652685 PMC2515157

[B20] Edgar R. C. (2010). Search and clustering orders of magnitude faster than BLAST. Bioinformatics 26, 2460–2461. 10.1093/bioinformatics/btq461 20709691

[B21] Felsenstein J. (1985). Confidence-limits on phylogenies - an approach using the bootstrap. Evolution 39, 783–791. 10.2307/2408678 28561359

[B22] Gerasimchuk A. L. Shatalov A. A. Novikov A. L. Butorova O. P. Pimenov N. V. Lein A. Y. (2010). The search for sulfate-reducing bacteria in mat samples from the lost city hydrothermal field by molecular cloning. Microbiology 79, 96–105. 10.1134/S0026261710010133 20411667

[B23] Good I. J. (1953). The population frequencies of species and the estimation of population parameters. Biometrika 40, 237–264. 10.1093/biomet/40.3-4.237

[B24] Haouari O. Fardeau M.-L. Cayol J. L. Casiot C. Elbaz-Poulichet F. Hamdi M.. (2008). *Desulfotomaculum hydrothermale* sp. nov., a thermophilic sulfate-reducing bacterium isolated from a terrestrial Tunisian hot spring. Int. J. Syst. Evol. Microbiol. 58, 2529–2535. 10.1099/ijs.0.65339-0 18984688

[B25] Hungate R. E. (1969). A roll tube method for cultivation of strict anaerobes. Method. Microbiol. 3B, 117–132. 10.1016/S0580-9517(08)70503-8

[B26] Imachi H. Sekiguchi Y. Kamagata Y. Loy A. Qiu Y. L. Hugenholtz P.. (2006). Non-sulfate-reducing, syntrophic bacteria affiliated with *Desulfotomaculum* cluster I are widely distributed in methanogenic environments. Appl. Environ. Microbiol. 72, 2080–2091. 10.1128/AEM.72.3.2080-2091.2006 16517657 PMC1393244

[B27] Kelley D. S. Karson J. A. Früh-Green G. L. Yoerger D. R. Shank T. M. Butterfield D. A.. (2005). A serpentinite-hosted ecosystem: the Lost City hydrothermal field. Science 307, 1428–1434. 10.1126/science.1102556 15746419

[B28] Lane D. J. (1991). Nucleic acid techniques in bacterial systematics, in Nucleic Acid Techniques in Bacterial Systematics, eds Stackebrandt E. Goodfellow M. (Hoboken, NJ: Wiley), 115–175.

[B29] Launay J. Fontes J. C. (1985). Les sources thermales de Prony (Nouvelle–Caledonie) et leurs precipites chimiques. Exemple de formation de brucite primaire. Geologie de la France 1, 83–100.

[B30] Liu J. F. Sun X. B. Yang G. C. Mbadinga S. M. Gu J. D. Mu B. Z. (2015). Analysis of microbial communities in the oil reservoir subjected to CO_2_-flooding by using functional genes as molecular biomarkers for microbial CO_2_ sequestration. Front. Microbiol. 6:236. 10.3389/fmicb.2015.00236 25873911 PMC4379918

[B31] Lubitz W. Ogata H. Rüdiger O. Reijerse E. (2014). Hydrogenases. Chem. Rev. 114, 4081–4148. 10.1021/cr4005814 24655035

[B32] Lüdemann H. Arth I. Liesack W. (2000). Spatial changes in the bacterial community structure along a vertical oxygen gradient in flooded paddy soil cores. Appl. Environ. Microbiol. 66, 754–762. 10.1128/AEM.66.2.754-762.2000 10653747 PMC91892

[B33] Mei N. Postec A. Erauso G. Joseph M. Pelletier B. Payri C.. (2016). *Serpentinicella alkaliphila gen*. nov., sp. nov., a novel alkaliphilic anaerobic bacterium isolated from the serpentinite-hosted Prony hydrothermal field, New Caledonia. Int. J. Syst. Evol. Microbiol. 10.1099/ijsem.0.001375. [Epub ahead of print]. 27499124

[B34] Mei N. Zergane N. Postec A. Erauso G. Ollier A. Payri C. (2014). Fermentative hydrogen production by a new alkaliphilic *Clostridium* sp. (strain PROH2) isolated from a shallow submarine hydrothermal chimney in Prony Bay, New Caledonia. Int. J. Hydrogen Energy 39, 19465–19473. 10.1016/j.ijhydene.2014.09.111

[B35] Meyer B. Kuehl J. Deutschbauer A. M. Price M. N. Arkin A. P. Stahl D. A. (2013). Variation among *Desulfovibrio* species in electron transfer systems used for syntrophic growth. J. Bacteriol. 195, 990–1004. 10.1128/JB.01959-12 23264581 PMC3571329

[B36] Meyer F. Paarmann D. D'Souza M. Olson R. Glass E. M. Kubal M.. (2008). The metagenomics RAST server–a public resource for the automatic phylogenetic and functional analysis of metagenomes. BMC Bioinformatics 9:386. 10.1186/1471-2105-9-386 18803844 PMC2563014

[B37] Miller H. M. Matter J. M. Kelemen P. Ellison E. T. Conrad M. E. Fierer N. (2016). Modern water/rock reactions in Oman hyperalkaline peridotite aquifers and implications for microbial habitability. Geochim. Cosmochim. Acta 179, 217–241. 10.1016/j.gca.2016.01.033

[B38] Monnin C. Chavagnac V. Boulart C. Ménez B. Gérard M. Gérard E. (2014). Fluid chemistry of the low temperature hyperalkaline hydrothermal system of the Prony Bay (New Caledonia). Biogeosciences 11, 5687–5706. 10.5194/bg-11-5687-2014

[B39] Pasini V. Brunelli D. Dumas P. Sandt C. Frederick J. Benzerara K. (2013). Low temperature hydrothermal oil and associated biological precursors in serpentinites from Mid-Ocean Ridge. Lithos 178, 84–95. 10.1016/j.lithos.2013.06.014

[B40] Peters J. W. Schut G. J. Boyd E. S. Mulder D. W. Shepard E. M. Broderick J. B. (2015). [FeFe]-and [NiFe]-hydrogenase diversity, mechanism, and maturation. Biochim. Biophys. Acta Mol. Cell Res. 1853, 1350–1369. 10.1016/j.bbamcr.2014.11.021 25461840

[B41] Pisapia C. Gérard E. Gérard M. Lecourt L. Pelletier B. Payri C. (in press). Mineralizing filamentous bacteria from the Prony bay Hydrothermal Field open new perspectives for serpentinization-based deep ecosystems. Front. Microbiol.. 10.3389/fmicb.2017.00057 PMC5281578 28197130

[B42] Postec A. Quéméneur M. Bes M. Mei N. BenaÏssa F. Payri C.. (2015). Microbial diversity in a submarine carbonate edifice from the serpentinizing hydrothermal system of the Prony Bay (New Caledonia) over a 6-year period. Front. Microbiol. 6:857. 10.3389/fmicb.2015.00857 26379636 PMC4551099

[B43] Poudel S. Tokmina-Lukaszewska M. Colman D. R. Refai M. Schut G. J. King P. W.. (2016). Unification of [FeFe]-hydrogenases into three structural and functional groups. Biochim. Biophys. Acta 1860, 1910–1921. 10.1016/j.bbagen.2016.05.034 27241847

[B44] Quéméneur M. Bes M. Postec A. Mei N. Hamelin J. Monnin C.. (2014). Spatial distribution of microbial communities in the shallow submarine alkaline hydrothermal field of the Prony Bay, New Caledonia. Environ. Microbiol. Rep. 6, 665–674. 10.1111/1758-2229.12184 25756120

[B45] Quéméneur M. Hamelin J. Latrille E. Steyer J.-P. Trably E. (2010). Development and application of a functional CE-SSCP fingerprinting method based on [Fe-Fe]-hydrogenase genes for monitoring hydrogen producing *Clostridium* in mixed cultures. Int. J. Hydrogen Energy 35, 13158–13167. 10.1016/j.ijhydene.2010.07.076

[B46] Quéméneur M. Hamelin J. Latrille E. Steyer J.-P. Trably E. (2011). Functional versus phylogenetic fingerprint analyses for monitoring hydrogen-producing bacterial populations in dark fermentation cultures. Int. J. Hydrogen Energy 36, 3870–3879. 10.1016/j.ijhydene.2010.12.100

[B47] Quéméneur M. Palvadeau A. Postec A. Monnin C. Chavagnac V. Ollivier B.. (2015). Endolithic microbial communities in carbonate precipitates from serpentinite-hosted hyperalkaline springs of the Voltri massif (Ligurian Alps, Northern Italy). Environ. Sci. Pollut. R. 22, 13613–13624. 10.1007/s11356-015-4113-7 25874424

[B48] Schmidt O. Drake H. L. Horn M. A. (2010). Hitherto unknown [Fe-Fe]-hydrogenase gene diversity in anaerobes and anoxic enrichments from a moderately acidic fen. Appl. Environ. Microbiol. 76, 2027–2031. 10.1128/AEM.02895-09 20118375 PMC2838027

[B49] Schrenk M. O. Brazelton W. J. Lang S. Q. (2013). Serpentinization, carbon, and deep life. Rev. Mineral. Geochem. 75, 575–606. 10.2138/rmg.2013.75.18

[B50] Schut G. J. Adams M. W. (2009). The iron-hydrogenase of *Thermotoga maritime* utilizes ferredoxin and NADH synergistically: a new perspective on anaerobic hydrogen production. J. Bacteriol. 191, 4451–4457. 10.1128/JB.01582-08 19411328 PMC2698477

[B51] Shannon C. E. Weaver W. (1949). The Mathematical Theory of Communication. Urbana, IL: University of Illinois Press.

[B52] Simpson E. H. (1949). Measurement of diversity. Nature 163, 688–688.

[B53] Suzuki S. Kuenen J. G. Schipper K. van der Velde S. Ishii S. I. Wu A.. (2014). Physiological and genomic features of highly alkaliphilic hydrogen-utilizing *Betaproteobacteria* from a continental serpentinizing site. Nat. Commun. 5:3900. 10.1038/ncomms4900 24845058 PMC4050266

[B54] Tamura K. Stecher G. Peterson D. Filipski A. Kumar S. (2013). MEGA6: molecular evolutionary genetics analysis version 6.0. Mol. Biol. Evol. 30, 2725–2729. 10.1093/molbev/mst197 24132122 PMC3840312

[B55] Thauer R. K. Jungermann K. Decker K. (1977). Energy conservation in chemotrophic anaerobic bacteria. Bacteriol. Rev. 41, 100. 860983 10.1128/br.41.1.100-180.1977 PMC413997

[B56] Tiago I. Veríssimo A. (2013). Microbial and functional diversity of a subterrestrial high pH groundwater associated to serpentinization. Environ. Microbiol. 15, 1687–1706. 10.1111/1462-2920.12034 23731249

[B57] Vignais P. M. Billoud B. Meyer J. (2001). Classification and phylogeny of hydrogenases. FEMS Microbiol. Rev. 25, 455–501. 10.1111/j.1574-6976.2001.tb00587.x 11524134

[B58] Vignais P. M. Colbeau A. (2004). Molecular biology of microbial hydrogenases. Curr. Issues Mol. Biol. 6, 159–188. 15119826

[B59] Wang J. Wan W. (2009). Factors influencing fermentative hydrogen production: a review. Int. J. Hydrogen Energy 34, 799–811. 10.1016/j.ijhydene.2008.11.015

[B60] Wang Q. Garrity G. M. Tiedje J. M. Cole J. R. (2007). Naive Bayesian classifier for rapid assignment of rRNA sequences into the new bacterial taxonomy. Appl. Environ. Microbiol. 73, 5261–5267. 10.1128/AEM.00062-07 17586664 PMC1950982

[B61] Wang S. Huang H. Kahnt J. Thauer R. K. (2013). A reversible electron-bifurcating ferredoxin-and NAD-dependent [FeFe]-hydrogenase (HydABC) in *Moorella thermoacetica*. J. Bacteriol. 195, 1267–1275. 10.1128/JB.02158-12 23316038 PMC3591994

[B62] Xiao B. Liu J. (2006). pH dependency of hydrogen fermentation from alkali-pretreated sludge. Chin. Sci. Bull. 51, 399–404. 10.1007/s11434-006-0399-7

[B63] Xing D. F. Ren N. Q. Rittmann B. E. (2008). Genetic diversity of hydrogen-producing bacteria in an acidophilic ethanol-H_2_-coproducing system, analyzed using the Fe-hydrogenase gene. Appl. Environ. Microbiol. 74, 1232–1239. 10.1128/AEM.01946-07 18156331 PMC2258583

