# Supplementary material for: Metagenomic and PCR-Based Diversity Surveys of [FeFe]-Hydrogenases Combined with Isolation of Alkaliphilic Hydrogen-Producing Bacteria from the Serpentinite-Hosted Prony Hydrothermal Field, New Caledonia
Source: Front Microbiol. 2016 Aug 30;7:1301. doi: 10.3389/fmicb.2016.01301 (PMC5003875; doi:10.3389/fmicb.2016.01301)
Supplement: Supplementary file 1 [file Table1.DOC]

**Table S1. Putative taxonomic affiliation of [FeFe]-hydrogenase sequences obtained from Prony metagenome P27.**

| OTU | MG-RAST ID | Taxonomy (Phylum ; Class ; Order) | Highly similar sequence retrieved from Genbank | | |
| --- | --- | --- | --- | --- | --- |
|  |  |  | % similarity | | (Genbank accession number) Species - clone |
| P27_hydA14 | D4ZHLFP1:60:D2HDNACXX:3:2107:8059:82633:ACACGA | *Firmicutes*; *Clostridia*; *Clostridiales* | 75.5 | (YP_008699055) hydrogenase, Fe-only [*Clostridium* *autoethanogenum* DSM 10061] | |
| P27_hydA114 | D4ZHLFP1:60:D2HDNACXX:3:1109:13172:94760:ACACGA | *Firmicutes*; *Clostridia*; *Clostridiales* | 68.2 | (WP_023062885) hydrogenase, Fe-only [*Clostridium* *thermocellum*] | |
| P27_hydA1 | D4ZHLFP1:60:D2HDNACXX:3:1311:20647:55845:ACACGA | *Firmicutes*; *Clostridia*; *Clostridiales* | 48.1 | (WP_023062885) hydrogenase, Fe-only [*Clostridium* *thermocellum*] | |
| P27_hydA7 | D4ZHLFP1:60:D2HDNACXX:3:2315:12483:17223:ACACGA | *Firmicutes*; *Clostridia*; *Clostridiales* | 77.4 | (WP_023062885) hydrogenase, Fe-only [*Clostridium* *thermocellum*] | |
| P27_hydA136 | D4ZHLFP1:60:D2HDNACXX:3:2306:7045:37162:ACACGA | *Firmicutes*; *Clostridia*; *Clostridiales* | 49.7 | (GAE89903) hydrogenase [*Clostridium* *straminisolvens* JCM 21531] | |
| P27_hydA45 | D4ZHLFP1:60:D2HDNACXX:3:2308:14654:24288:ACACGA | *Firmicutes*; *Clostridia*; *Clostridiales* | 65.9 | (GAE89903) hydrogenase [*Clostridium* *straminisolvens* JCM 21531] | |
| P27_hydA129 | D4ZHLFP1:60:D2HDNACXX:3:2306:11382:44214:ACACGA | *Firmicutes*; *Clostridia*; *Clostridiales* | 72.4 | (YP_005047780) hydrogenase, Fe-only [*Clostridium* *clariflavum* DSM 19732] | |
| P27_hydA13 | D4ZHLFP1:60:D2HDNACXX:3:2102:8481:71964:ACACGA | *Firmicutes*; *Clostridia*; *Clostridiales* | 75.1 | (YP_008699055) hydrogenase, Fe-only [*Clostridium* *autoethanogenum* DSM 10061] | |
| P27_hydA84 | D4ZHLFP1:60:D2HDNACXX:3:1106:2370:35020:ACACGA | *Firmicutes*; *Clostridia*; *Clostridiales* | 76.3 | (WP_022116126) hydrogenase Fe-only [*Clostridium* sp. CAG:169] | |
| P27_hydA85 | D4ZHLFP1:60:D2HDNACXX:3:2305:8704:48944:ACACGA | *Firmicutes*; *Clostridia*; *Clostridiales* | 85.1 | (YP_001036861) hydrogenase, Fe-only [*Clostridium* *thermocellum* ATCC 27405] | |
| P27_hydA48 | D4ZHLFP1:60:D2HDNACXX:3:1108:1705:91727:ACACGA | *Firmicutes*; *Clostridia*; *Clostridiales* | 78.6 | (YP_004517644) hydrogenase, Fe-only [*Desulfotomaculum* *kuznetsovii* DSM 6115] | |
| P27_hydA5 | D4ZHLFP1:60:D2HDNACXX:3:1314:8272:100789:ACACGA | *Firmicutes*; *Clostridia*; *Clostridiales* | 80.9 | (YP_004518295) hydrogenase, Fe-only [*Desulfotomaculum* *kuznetsovii* DSM 6115] | |
| P27_hydA106 | D4ZHLFP1:60:D2HDNACXX:3:2306:18633:67353:ACACGA | *Firmicutes*; *Clostridia*; *Clostridiales* | 71.6 | (YP_004518295) hydrogenase, Fe-only [*Desulfotomaculum* *kuznetsovii* DSM 6115] | |
| P27_hydA101 | D4ZHLFP1:60:D2HDNACXX:3:2314:5473:6798:ACACGA | *Firmicutes*; *Clostridia*; *Clostridiales* | 48.5 | (YP_001113004) hydrogenase [*Desulfotomaculum* *reducens* MI-1] | |
| P27_hydA24 | D4ZHLFP1:60:D2HDNACXX:3:2314:9285:21359:ACACGA | *Firmicutes*; *Clostridia*; *Clostridiales* | 90.0 | (YP_004545921) hydrogenase Fe-only [*Desulfotomaculum* *ruminis* DSM 2154] | |
| P27_hydA27 | D4ZHLFP1:60:D2HDNACXX:3:1305:16629:82167:ACACGA | *Firmicutes*; *Clostridia*; *Clostridiales* | 77.8 | (YP_004518295) hydrogenase, Fe-only [*Desulfotomaculum* *kuznetsovii* DSM 6115] | |
| P27_hydA107 | D4ZHLFP1:60:D2HDNACXX:3:1114:19745:58907:ACACGA | *Firmicutes*; *Clostridia*; *Clostridiales* | 77.4 | (YP_001113007) hydrogenase [*Desulfotomaculum* *reducens* MI-1] | |
| P27_hydA11 | D4ZHLFP1:60:D2HDNACXX:3:2116:12176:59324:ACACGA | *Firmicutes*; *Clostridia*; *Clostridiales* | 77.8 | (YP_004518295) hydrogenase, Fe-only [*Desulfotomaculum* *kuznetsovii* DSM 6115] | |
| P27_hydA111 | D4ZHLFP1:60:D2HDNACXX:3:2106:3631:63778:ACACGA | *Firmicutes*; *Clostridia*; *Clostridiales* | 79.0 | (WP_008410064) Iron hydrogenase 1 [*Desulfotomaculum* *hydrothermale*] | |
| P27_hydA134 | D4ZHLFP1:60:D2HDNACXX:3:1303:11680:47793:ACACGA | *Firmicutes*; *Clostridia*; *Clostridiales* | 97.1 | (YP_004518295) hydrogenase, Fe-only [*Desulfotomaculum* *kuznetsovii* DSM 6115] | |
| P27_hydA135 | D4ZHLFP1:60:D2HDNACXX:3:2316:14697:15311:ACACGA | *Firmicutes*; *Clostridia*; *Clostridiales* | 94.0 | (YP_004517644) hydrogenase, Fe-only [*Desulfotomaculum* *kuznetsovii* DSM 6115] | |
| P27_hydA123 | D4ZHLFP1:60:D2HDNACXX:3:2302:3021:14439:ACACGA | *Firmicutes*; *Clostridia*; *Clostridiales* | 81.6 | (YP_004517644) hydrogenase, Fe-only [*Desulfotomaculum* *kuznetsovii* DSM 6115] | |
| P27_hydA19 | D4ZHLFP1:60:D2HDNACXX:3:1309:20698:19893:ACACGA | *Firmicutes*; *Clostridia*; *Clostridiales* | 91.0 | (YP_004517644) hydrogenase, Fe-only [*Desulfotomaculum* *kuznetsovii* DSM 6115] | |
| P27_hydA51 | D4ZHLFP1:60:D2HDNACXX:3:2110:4908:93477:ACACGA | *Firmicutes*; *Clostridia*; *Clostridiales* | 95.0 | (YP_003189750) hydrogenase, Fe-only [*Desulfotomaculum* *acetoxidans* DSM 771] | |
| P27_hydA90 | D4ZHLFP1:60:D2HDNACXX:3:2308:4104:27466:ACACGA | *Firmicutes*; *Clostridia*; *Clostridiales* | 90.5 | (YP_001113004) hydrogenase [*Desulfotomaculum* *reducens* MI-1] | |
| P27_hydA91 | D4ZHLFP1:60:D2HDNACXX:3:2301:6794:9875:ACACGA | *Firmicutes*; *Clostridia*; *Clostridiales* | 95.0 | (YP_003189750) hydrogenase, Fe-only [*Desulfotomaculum* *acetoxidans* DSM 771] | |
| P27_hydA92 | D4ZHLFP1:60:D2HDNACXX:3:2312:14796:69310:ACACGA | *Firmicutes*; *Clostridia*; *Clostridiales* | 95.9 | (YP_004545921) hydrogenase Fe-only [*Desulfotomaculum* *ruminis* DSM 2154] | |
| P27_hydA68 | D4ZHLFP1:60:D2HDNACXX:3:1115:15760:55593:ACACGA | *Firmicutes*; *Clostridia*; *Clostridiales* | 66.6 | (YP_004969732) hydrogenase, Fe-only [*Desulfosporosinus orientis* DSM 765] | |
| P27_hydA93 | D4ZHLFP1:60:D2HDNACXX:3:2316:7439:59410:ACACGA | *Firmicutes*; *Clostridia*; *Clostridiales* | 84.3 | (YP_004518295) hydrogenase, Fe-only [*Desulfotomaculum* *kuznetsovii* DSM 6115] | |
| P27_hydA96 | D4ZHLFP1:60:D2HDNACXX:3:2302:9663:61279:ACACGA | *Firmicutes*; *Clostridia*; *Clostridiales* | 68.2 | (WP_008410064) Iron hydrogenase 1 [*Desulfotomaculum* *hydrothermale*] | |
| P27_hydA59 | D4ZHLFP1:60:D2HDNACXX:3:1307:7139:36512:ACACGA | *Firmicutes*; *Clostridia*; *Clostridiales* | 90.5 | (YP_007946226) hydrogenase, Fe-only [*Desulfotomaculum* *gibsoniae* DSM 7213] | |
| P27_hydA37 | D4ZHLFP1:60:D2HDNACXX:3:1311:12003:89454:ACACGA | *Firmicutes*; *Clostridia*; *Clostridiales* | 90.9 | (YP_003189750) hydrogenase, Fe-only [*Desulfotomaculum* *acetoxidans* DSM 771] | |
| P27_hydA38 | D4ZHLFP1:60:D2HDNACXX:3:2313:17826:33583:ACACGA | *Firmicutes*; *Clostridia*; *Clostridiales* | 90.9 | (YP_004517644) hydrogenase, Fe-only [*Desulfotomaculum* *kuznetsovii* DSM 6115] | |
| P27_hydA128 | D4ZHLFP1:60:D2HDNACXX:3:1303:17006:29935:ACACGA | *Firmicutes*; *Clostridia*; *Clostridiales* | 93.0 | (WP_008410059) Iron hydrogenase 1 [*Desulfotomaculum* *hydrothermale*] | |
| P27_hydA130 | D4ZHLFP1:60:D2HDNACXX:3:2115:12121:70737:ACACGA | *Firmicutes*; *Clostridia*; *Clostridiales* | 84.0 | (YP_004517644) hydrogenase, Fe-only [*Desulfotomaculum* *kuznetsovii* DSM 6115] | |
| P27_hydA131 | D4ZHLFP1:60:D2HDNACXX:3:2110:11779:25039:ACACGA | *Firmicutes*; *Clostridia*; *Clostridiales* | 90.9 | (WP_008410059) Iron hydrogenase 1 [*Desulfotomaculum* *hydrothermale*] | |
| P27_hydA79 | D4ZHLFP1:60:D2HDNACXX:3:1315:7322:7202:ACACGA | *Firmicutes*; *Clostridia*; *Clostridiales* | 63.2 | (YP_004518295) hydrogenase, Fe-only [*Desulfotomaculum* *kuznetsovii* DSM 6115] | |
| P27_hydA8 | D4ZHLFP1:60:D2HDNACXX:3:2109:10707:54209:ACACGA | *Firmicutes*; *Clostridia*; *Clostridiales* | 93.6 | (YP_004518238) hydrogenase, Fe-only [*Desulfotomaculum* *kuznetsovii* DSM 6115] | |
| P27_hydA97 | D4ZHLFP1:60:D2HDNACXX:3:1309:12380:17569:ACACGA | *Firmicutes*; *Clostridia*; *Clostridiales* | 72.0 | (YP_004496996) hydrogenase, Fe-only [*Desulfotomaculum* *carboxydivorans* CO-1-SRB] | |
| P27_hydA61 | D4ZHLFP1:60:D2HDNACXX:3:2304:9197:41505:ACACGA | *Firmicutes*; *Clostridia*; *Clostridiales* | 85.9 | (YP_004497002) hydrogenase, Fe-only [*Desulfotomaculum* *carboxydivorans* CO-1-SRB] | |
| P27_hydA63 | D4ZHLFP1:60:D2HDNACXX:3:1106:12199:34111:ACACGA | *Firmicutes*; *Clostridia*; *Clostridiales* | 91.0 | (YP_003189750) hydrogenase, Fe-only [*Desulfotomaculum* *acetoxidans* DSM 771] | |
| P27_hydA64 | D4ZHLFP1:60:D2HDNACXX:3:2303:2598:25384:ACACGA | *Firmicutes*; *Clostridia*; *Clostridiales* | 92.0 | (YP_004545921) hydrogenase Fe-only [*Desulfotomaculum* *ruminis* DSM 2154] | |
| P27_hydA72 | D4ZHLFP1:60:D2HDNACXX:3:1302:19141:50190:ACACGA | *Firmicutes*; *Clostridia*; *Clostridiales* | 65.1 | (YP_004518295) hydrogenase, Fe-only [*Desulfotomaculum* *kuznetsovii* DSM 6115] | |
| P27_hydA43 | D4ZHLFP1:60:D2HDNACXX:3:2314:1930:100125:ACACGA | *Firmicutes*; *Clostridia*; *Clostridiales* | 73.6 | (WP_022271995) hydrogenase Fe-only [*Eubacterium siraeum* CAG:80] | |
| P27_hydA125 | D4ZHLFP1:60:D2HDNACXX:3:2314:16699:37745:ACACGA | *Firmicutes*; *Clostridia*; *Clostridiales* | 79.3 | (YP_077035) Fe hydrogenase [*Symbiobacterium thermophilum* IAM 14863] | |
| P27_hydA126 | D4ZHLFP1:60:D2HDNACXX:3:2310:17631:29511:ACACGA | *Firmicutes*; *Clostridia*; *Clostridiales* | 49.7 | (YP_003640841) hydrogenase, Fe-only [*Thermincola potens* JR] | |
| P27_hydA47 | D4ZHLFP1:60:D2HDNACXX:3:1313:3186:85130:ACACGA | *Firmicutes*; *Clostridia*; *Clostridiales* | 71.2 | (YP_003640841) hydrogenase, Fe-only [*Thermincola potens* JR] | |
| P27_hydA75 | D4ZHLFP1:60:D2HDNACXX:3:2314:7403:99385:ACACGA | *Firmicutes*; *Clostridia*; *Clostridiales* | 67.0 | (WP_021908252) hydrogenase Fe-only [*Eubacterium* sp. CAG:146] | |
| P27_hydA53 | D4ZHLFP1:60:D2HDNACXX:3:2304:9596:32957:ACACGA | *Firmicutes*; *Clostridia*; *Clostridiales* | 98.1 | (YP_006910651) [Fe] hydrogenase, large subunit HymC [*Dehalobacter* sp. DCA] | |
| P27_hydA56 | D4ZHLFP1:60:D2HDNACXX:3:2307:5017:82140:ACACGA | *Firmicutes*; *Clostridia*; *Clostridiales* | 75.1 | (YP_003640841) hydrogenase, Fe-only [*Thermincola potens* JR] | |
| P27_hydA78 | D4ZHLFP1:60:D2HDNACXX:3:2308:19195:51114:ACACGA | *Firmicutes*; *Clostridia*; *Halanaerobiales* | 59.3 | (YP_003826883) NAD(P)-dependent iron-only hydrogenase iron-iron [*Acetohalobium arabaticum* DSM 5501] | |
| P27_hydA52 | D4ZHLFP1:60:D2HDNACXX:3:1313:15591:68584:ACACGA | *Firmicutes*; *Clostridia*; *Thermoanaerobacterales* | 76.6 | (YP_003239720) hydrogenase, Fe-only [*Ammonifex degensii* KC4] | |
| P27_hydA31 | D4ZHLFP1:60:D2HDNACXX:3:1101:20130:2900:ACACGA | *Firmicutes*; *Clostridia*; *Thermoanaerobacterales* | 78.6 | ( ACA51661) HydA [*Thermoanaerobacterium saccharolyticum* JW/SL-YS485] | |
| P27_hydA117 | D4ZHLFP1:60:D2HDNACXX:3:2316:6701:34903:ACACGA | *Firmicutes*; *Clostridia*; *Thermoanaerobacterales* | 98.6 | (YP_007298421) hydrogenase, Fe-only [*Thermoanaerobacterium thermosaccharolyticum* M0795] | |
| P27_hydA121 | D4ZHLFP1:60:D2HDNACXX:3:2315:6287:77241:ACACGA | *Firmicutes*; *Clostridia*; *Thermoanaerobacterales* | 97.8 | (YP_007298421) hydrogenase, Fe-only [*Thermoanaerobacterium thermosaccharolyticum* M0795] | |
| P27_hydA4 | D4ZHLFP1:60:D2HDNACXX:3:2315:14097:33168:ACACGA | *Firmicutes*; *Clostridia*; *Thermoanaerobacterales* | 87.0 | (GAF24846) iron only hydrogenase large subunit, C-terminal [*Moorella thermoacetica* Y72] | |
| P27_hydA35 | D4ZHLFP1:60:D2HDNACXX:3:2308:15765:97985:ACACGA | *Thermotogae*; *Thermotogae*; *Thermotogales* | 96.3 | (YP_005096723) hydrogenase, Fe-only [*Marinitoga piezophila* KA3] | |
| P27_hydA18 | D4ZHLFP1:60:D2HDNACXX:3:2313:17713:85556:ACACGA | *Thermotogae*; *Thermotogae*; *Thermotogales* | 83.2 | (YP_005470551) hydrogenase, Fe-only [*Fervidobacterium pennivorans* DSM 9078] | |
| P27_hydA23 | D4ZHLFP1:60:D2HDNACXX:3:1114:2287:87019:ACACGA | *Thermotogae*; *Thermotogae*; *Thermotogales* | 82.8 | (YP_004659299) hydrogenase large subunit domain-containing protein [*Thermotoga thermarum* DSM 5069] | |
| P27_hydA26 | D4ZHLFP1:60:D2HDNACXX:3:1107:12202:54331:ACACGA | *Thermotogae*; *Thermotogae*; *Thermotogales* | 54.3 | (YP_002534027) NADP-reducing hydrogenase, subunit D [*Thermotoga neapolitana* DSM 4359] | |
| P27_hydA133 | D4ZHLFP1:60:D2HDNACXX:3:1311:8497:2283:ACACGA | *Thermotogae*; *Thermotogae*; *Thermotogales* | 89.0 | (YP_005096723) hydrogenase, Fe-only [*Marinitoga piezophila* KA3] | |
| P27_hydA108 | D4ZHLFP1:60:D2HDNACXX:3:2311:19981:34912:ACACGA | *Deltaproteobacteria*; *Syntrophobacterales* | 90.1 | (YP_844976) hydrogenase, Fe-only [*Syntrophobacter fumaroxidans* MPOB] | |
| P27_hydA70 | D4ZHLFP1:60:D2HDNACXX:3:2312:3330:10832:ACACGA | *Deltaproteobacteria*; *Syntrophobacterales* | 97.1 | (YP_844976) hydrogenase, Fe-only [*Syntrophobacter fumaroxidans* MPOB] | |
| P27_hydA103 | D4ZHLFP1:60:D2HDNACXX:3:2309:13748:77439:ACACGA | *Deltaproteobacteria*; *Desulfovibrionales* | 90.1 | (YP_002952160) Fe hydrogenase [*Desulfovibrio magneticus* RS-1] | |
| P27_hydA15 | D4ZHLFP1:60:D2HDNACXX:3:2312:18659:40581:ACACGA | *Deltaproteobacteria*; *Desulfovibrionales* | 84.3 | (WP_006921111) hydrogenase, Fe-only, partial [*Desulfovibrio magneticus*] | |
| P27_hydA118 | D4ZHLFP1:60:D2HDNACXX:3:2101:19990:24288:ACACGA | *Deltaproteobacteria*; *Desulfuromonadales* | 82.0 | (YP_004198598) hydrogenase, Fe-only [*Geobacter* sp. M18] | |
| P27_hydA16 | D4ZHLFP1:60:D2HDNACXX:3:2305:7417:37924:ACACGA | *Deltaproteobacteria*; *Desulfobacterales* | 87.8 | (YP_004195044) NAD(P)-dependent iron-only hydrogenase catalytic subunit [*Desulfobulbus propionicus* DSM 2032] | |
| P27_hydA21 | D4ZHLFP1:60:D2HDNACXX:3:2315:11400:82351:ACACGA | *Deltaproteobacteria*; *Desulfobacterales* | 77.8 | (YP_004195044) NAD(P)-dependent iron-only hydrogenase catalytic subunit [*Desulfobulbus propionicus* DSM 2032] | |
| P27_hydA74 | D4ZHLFP1:60:D2HDNACXX:3:1310:5971:76924:ACACGA | *Deltaproteobacteria*; *Desulfobacterales* | 71.2 | (YP_004195044) NAD(P)-dependent iron-only hydrogenase catalytic subunit [*Desulfobulbus propionicus* DSM 2032] | |
| P27_hydA119 | D4ZHLFP1:60:D2HDNACXX:3:2116:8151:3767:ACACGA | *Synergistetes*; *Synergistia*; *Synergistales* | 81.3 | (YP_003316672) hydrogenase [*Thermanaerovibrio acidaminovorans* DSM 6589] | |
| P27_hydA104 | D4ZHLFP1:60:D2HDNACXX:3:2301:7976:40736:ACACGA | *Synergistetes*; *Synergistia*; *Synergistales* | 78.6 | (YP_003316672) hydrogenase [*Thermanaerovibrio acidaminovorans* DSM 6589] | |
| P27_hydA110 | D4ZHLFP1:60:D2HDNACXX:3:1313:7546:14195:ACACGA | *Spirochaetes*; *Spirochaetia*; *Spirochaetales* | 95.0 | (YP_005475514) hydrogenase, Fe-only [*Spirochaeta africana* DSM 8902] | |
| P27_hydA124 | D4ZHLFP1:60:D2HDNACXX:3:2304:6980:81620:ACACGA | *Spirochaetes*; *Spirochaetia*; *Spirochaetales* | 77.8 | (YP_005475514) hydrogenase, Fe-only [*Spirochaeta africana* DSM 8902] | |
| P27_hydA3 | D4ZHLFP1:60:D2HDNACXX:3:2311:2271:81749:ACACGA | *Spirochaetes*; *Spirochaetia*; *Spirochaetales* | 87.4 | (YP_003802215) NAD(P)-dependent iron-only hydrogenase catalytic subunit [*Spirochaeta smaragdinae* DSM 11293] | |
| P27_hydA32 | D4ZHLFP1:60:D2HDNACXX:3:2313:10656:87661:ACACGA | *Spirochaetes*; *Spirochaetia*; *Spirochaetales* | 96.0 | (YP_003802215) NAD(P)-dependent iron-only hydrogenase catalytic subunit [*Spirochaeta smaragdinae* DSM 11293] | |
| P27_hydA25 | D4ZHLFP1:60:D2HDNACXX:3:2314:19579:3636:ACACGA | *Spirochaetes*; *Spirochaetia*; *Spirochaetales* | 90.5 | (YP_003802215) NAD(P)-dependent iron-only hydrogenase catalytic subunit [*Spirochaeta smaragdinae* DSM 11293] | |
| P27_hydA49 | D4ZHLFP1:60:D2HDNACXX:3:2116:7098:31541:ACACGA | *Spirochaetes*; *Spirochaetia*; *Spirochaetales* | 71.6 | (WP_018525843) hydrogenase Fe-only [*Spirochaeta alkalica*] | |
| P27_hydA46 | D4ZHLFP1:60:D2HDNACXX:3:2305:18503:27092:ACACGA | *Spirochaetes*; *Spirochaetia*; *Spirochaetales* | 75.1 | (YP_005475514) hydrogenase, Fe-only [*Spirochaeta africana* DSM 8902] | |
| P27_hydA54 | D4ZHLFP1:60:D2HDNACXX:3:2306:9291:27493:ACACGA | *Spirochaetes*; *Spirochaetia*; *Spirochaetales* | 75.1 | (YP_005475514) hydrogenase, Fe-only [*Spirochaeta africana* DSM 8902] | |
| P27_hydA55 | D4ZHLFP1:60:D2HDNACXX:3:2308:18720:99679:ACACGA | *Spirochaetes*; *Spirochaetia*; *Spirochaetales* | 90.9 | (YP_003802215) NAD(P)-dependent iron-only hydrogenase catalytic subunit [*Spirochaeta smaragdinae* DSM 11293] | |
| P27_hydA71 | D4ZHLFP1:60:D2HDNACXX:3:2308:7319:15774:ACACGA | *Spirochaetes*; *Spirochaetia*; *Spirochaetales* | 85.9 | (YP_003802215) NAD(P)-dependent iron-only hydrogenase catalytic subunit [*Spirochaeta smaragdinae* DSM 11293] | |
| P27_hydA73 | D4ZHLFP1:60:D2HDNACXX:3:1107:7828:16538:ACACGA | *Spirochaetes*; *Spirochaetia*; *Spirochaetales* | 95.9 | (YP_003802215) NAD(P)-dependent iron-only hydrogenase catalytic subunit [*Spirochaeta smaragdinae* DSM 11293] | |
| P27_hydA86 | D4ZHLFP1:60:D2HDNACXX:3:1312:2867:61608:ACACGA | *Spirochaetes*; *Spirochaetia*; *Spirochaetales* | 70.1 | (YP_003802215) NAD(P)-dependent iron-only hydrogenase catalytic subunit [*Spirochaeta smaragdinae* DSM 11293] | |
| P27_hydA87 | D4ZHLFP1:60:D2HDNACXX:3:2308:16490:49170:ACACGA | *Spirochaetes*; *Spirochaetia*; *Spirochaetales* | 78.2 | (YP_003802215) NAD(P)-dependent iron-only hydrogenase catalytic subunit [*Spirochaeta smaragdinae* DSM 11293] | |
| P27_hydA88 | D4ZHLFP1:60:D2HDNACXX:3:1116:7681:64020:ACACGA | *Spirochaetes*; *Spirochaetia*; *Spirochaetales* | 88.6 | (YP_003802215) NAD(P)-dependent iron-only hydrogenase catalytic subunit [*Spirochaeta smaragdinae* DSM 11293] | |
| P27_hydA89 | D4ZHLFP1:60:D2HDNACXX:3:2312:19934:93270:ACACGA | *Spirochaetes*; *Spirochaetia*; *Spirochaetales* | 87.4 | (YP_005061073) hydrogenase, Fe-only [*Sphaerochaeta pleomorpha* str. Grapes] | |
| P27_hydA9 | D4ZHLFP1:60:D2HDNACXX:3:2113:4052:37186:ACACGA | *Spirochaetes*; *Spirochaetia*; *Spirochaetales* | 82.0 | (YP_003802215) NAD(P)-dependent iron-only hydrogenase catalytic subunit [*Spirochaeta smaragdinae* DSM 11293] | |
| P27_hydA94 | D4ZHLFP1:60:D2HDNACXX:3:1313:19524:59750:ACACGA | *Spirochaetes*; *Spirochaetia*; *Spirochaetales* | 88.6 | (YP_003802215) NAD(P)-dependent iron-only hydrogenase catalytic subunit [*Spirochaeta smaragdinae* DSM 11293] | |
| P27_hydA95 | D4ZHLFP1:60:D2HDNACXX:3:2315:16166:74536:ACACGA | *Spirochaetes*; *Spirochaetia*; *Spirochaetales* | 81.3 | (YP_005475514) hydrogenase, Fe-only [*Spirochaeta africana* DSM 8902] | |
| P27_hydA12 | D4ZHLFP1:60:D2HDNACXX:3:2306:15468:39944:ACACGA | *Bacteroidetes*; *Bacteroidia*; *Bacteroidales* | 81.3 | (AGY54346) Iron hydrogenase 1 [*Bacteroidales* bacterium CF] | |
| P27_hydA57 | D4ZHLFP1:60:D2HDNACXX:3:1307:6493:50547:ACACGA | *Bacteroidetes*; *Bacteroidia*; *Bacteroidales* | 72.4 | (WP_010803103) [FeFe] hydrogenase, group A [*Parabacteroides*] | |
| P27_hydA120 | D4ZHLFP1:60:D2HDNACXX:3:2312:6660:84593:ACACGA | Paddy field soil, Japan | 79.0 | (BAM65990) [FeFe]-hydrogenase, partial [uncultured bacterium] | |
| P27_hydA10 | D4ZHLFP1:60:D2HDNACXX:3:1306:1891:68256:ACACGA | Paddy field soil, Japan | 99.4 | (BAM66117) [FeFe]-hydrogenase, partial [uncultured bacterium] | |
| P27_hydA100 | D4ZHLFP1:60:D2HDNACXX:3:1313:6957:74344:ACACGA | Paddy field soil, Japan | 94.7 | (BAM65873) [FeFe]-hydrogenase, partial [uncultured bacterium] | |
| P27_hydA102 | D4ZHLFP1:60:D2HDNACXX:3:2308:10616:100738:ACACGA | Paddy field soil, Japan | 77.4 | (BAM65979) [FeFe]-hydrogenase, partial [uncultured bacterium] | |
| P27_hydA82 | D4ZHLFP1:60:D2HDNACXX:3:2309:13108:22639:ACACGA | Paddy field soil, Japan | 82.8 | (BAM65975) [FeFe]-hydrogenase, partial [uncultured bacterium] | |
| P27_hydA83 | D4ZHLFP1:60:D2HDNACXX:3:1113:15517:30091:ACACGA | Paddy field soil, Japan | 82.8 | (BAM66175) [FeFe]-hydrogenase, partial [uncultured bacterium] | |
| P27_hydA80 | D4ZHLFP1:60:D2HDNACXX:3:2115:2278:49486:ACACGA | Paddy field soil, Japan | 79.0 | (BAM66042) [FeFe]-hydrogenase, partial [uncultured bacterium] | |
| P27_hydA76 | D4ZHLFP1:60:D2HDNACXX:3:2309:7462:55567:ACACGA | Paddy field soil, Japan | 94.0 | (BAM66088) [FeFe]-hydrogenase, partial [uncultured bacterium] | |
| P27_hydA58 | D4ZHLFP1:60:D2HDNACXX:3:2302:12090:96382:ACACGA | Paddy field soil, Japan | 82.4 | (BAM65896) [FeFe]-hydrogenase, partial [uncultured bacterium] | |
| P27_hydA62 | D4ZHLFP1:60:D2HDNACXX:3:2313:1840:13283:ACACGA | Paddy field soil, Japan | 90.1 | (BAM65896) [FeFe]-hydrogenase, partial [uncultured bacterium] | |
| P27_hydA40 | D4ZHLFP1:60:D2HDNACXX:3:2305:19590:12472:ACACGA | Paddy field soil, Japan | 82.0 | (BAM65896) [FeFe]-hydrogenase, partial [uncultured bacterium] | |
| P27_hydA41 | D4ZHLFP1:60:D2HDNACXX:3:2311:18282:13431:ACACGA | Paddy field soil, Japan | 80.1 | (BAM65896) [FeFe]-hydrogenase, partial [uncultured bacterium] | |
| P27_hydA29 | D4ZHLFP1:60:D2HDNACXX:3:2108:7635:75958:ACACGA | Paddy field soil, Japan | 99.4 | (BAM66039) [FeFe]-hydrogenase, partial [uncultured bacterium] | |
| P27_hydA22 | D4ZHLFP1:60:D2HDNACXX:3:1116:13594:62969:ACACGA | Paddy field soil, Japan | 81.3 | (BAM66042) [FeFe]-hydrogenase, partial [uncultured bacterium] | |
| P27_hydA20 | D4ZHLFP1:60:D2HDNACXX:3:2316:13431:6449:ACACGA | Paddy field soil, Japan | 70.9 | (BAM65871) [FeFe]-hydrogenase, partial [uncultured bacterium] | |
| P27_hydA127 | D4ZHLFP1:60:D2HDNACXX:3:1111:13539:41334:ACACGA | Paddy field soil, Japan | 62.0 | (BAM66154) [FeFe]-hydrogenase, partial [uncultured bacterium] | |
| P27_hydA81 | D4ZHLFP1:60:D2HDNACXX:3:2314:16120:39436:ACACGA | Saline microbial mat community, Mexico | 98.2 | (ACM67558) iron-dependent hydrogenase [uncultured organism] clone="hydA_2 | |
| P27_hydA81 | D4ZHLFP1:60:D2HDNACXX:3:2314:16120:39436:ACACGA | Yellowstone Geothermal Ecosystem, USA | 96.3 | (ADC53604) [FeFe]-hydrogenase, [uncultured bacterium] clone E4_24 | |
| P27_hydA116 | D4ZHLFP1:60:D2HDNACXX:3:2304:16632:31364:ACACGA | Oil fields suBdJected to CO_2_ and water-flooding | 77.4 | (AGU38562) [FeFe]-hydrogenase, partial [uncultured prokaryote] clone="FeFe-Hyd_W4-27 | |
| P27_hydA65 | D4ZHLFP1:60:D2HDNACXX:3:2113:3477:30709:ACACGA | Saline microbial mat community, Mexico | 97.1 | (ACM67583) iron-dependent hydrogenase [uncultured organism] clone="hydA_60 | |
| P27_hydA17 | D4ZHLFP1:60:D2HDNACXX:3:2302:13048:20570:ACACGA | Water of high temperature petroleum reservoir | 85.5 | (AGU38562) [FeFe]-hydrogenase, production water of high temperature petroleum reservoir | |
| P27_hydA33 | D4ZHLFP1:60:D2HDNACXX:3:1114:20033:69531:ACACGA | Yellowstone Geothermal Ecosystem, USA | 93.2 | (ADC53598) [FeFe]-hydrogenase, [uncultured bacterium] | |
