# Supplementary material for: Metagenomic and PCR-Based Diversity Surveys of [FeFe]-Hydrogenases Combined with Isolation of Alkaliphilic Hydrogen-Producing Bacteria from the Serpentinite-Hosted Prony Hydrothermal Field, New Caledonia
Source: Front Microbiol. 2016 Aug 30;7:1301. doi: 10.3389/fmicb.2016.01301 (PMC5003875; doi:10.3389/fmicb.2016.01301)
Supplement: Supplementary file 2 [file Table2.DOC]

**Table S2. Putative taxonomic affiliation of [FeFe]-hydrogenase sequences obtained from Prony metagenome P28.**

| OTU | MG-RAST ID | Taxonomy (Phylum ; Class ; Order) | Highly similar sequence retrieved from Genbank | |  |
| --- | --- | --- | --- | --- | --- |
|  |  |  | % similarity | (Genbank accession number) Species - clone |  |
| P28_hydA157 | D4ZHLFP1:60:D2HDNACXX:2:2305:8886:3411:GGATGT | *Bacteroidetes*; Ignavibacteriae; Ignavibacteria | 85.9 | (YP_006526890) Fe-only hydrogenase, catalytic subunit alpha [*Melioribacter* *roseus* P3M-2] |  |
| P28_hydA192 | D4ZHLFP1:60:D2HDNACXX:2:1111:17909:53680:GGATGT | *Bacteroidetes*; *Bacteroidia*; *Bacteroidales* | 84.3 | (WP_010803103) [FeFe] hydrogenase, group A [*Parabacteroides*] |  |
| P28_hydA236 | D4ZHLFP1:60:D2HDNACXX:2:2302:10344:19825:GGATGT | *Bacteroidetes*; *Bacteroidia*; *Bacteroidales* | 75.9 | (WP_010803103) [FeFe] hydrogenase, group A [*Parabacteroides*] |  |
| P28_hydA276 | D4ZHLFP1:60:D2HDNACXX:2:1113:8675:13552:GGATGT | *Bacteroidetes*; *Bacteroidia*; *Bacteroidales* | 89.0 | (WP_021928742) hydrogenase Fe-only [*Alistipes* sp. CAG:831] |  |
| P28_hydA342 | D4ZHLFP1:60:D2HDNACXX:2:2107:20273:48824:GGATGT | *Bacteroidetes*; *Bacteroidia*; *Bacteroidales* | 81.6 | (WP_021928742) hydrogenase Fe-only [*Alistipes* sp. CAG:831] |  |
| P28_hydA35 | D4ZHLFP1:60:D2HDNACXX:2:2114:10078:90816:GGATGT | *Bacteroidetes*; *Bacteroidia*; *Bacteroidales* | 73.6 | (WP_021928742) hydrogenase Fe-only [*Alistipes* sp. CAG:831] |  |
| P28_hydA357 | D4ZHLFP1:60:D2HDNACXX:2:1313:16042:29537:GGATGT | *Bacteroidetes*; *Bacteroidia*; *Bacteroidales* | 63.9 | (WP_021928742) hydrogenase Fe-only [*Alistipes* sp. CAG:831] |  |
| P28_hydA445 | D4ZHLFP1:60:D2HDNACXX:2:2114:14460:89265:GGATGT | *Bacteroidetes*; *Bacteroidia*; *Bacteroidales* | 86.7 | (WP_021928742) hydrogenase Fe-only [*Alistipes* sp. CAG:831] |  |
| P28_hydA96 | D4ZHLFP1:60:D2HDNACXX:2:1302:11799:41654:GGATGT | *Bacteroidetes*; *Bacteroidia*; *Bacteroidales* | 70.9 | (WP_021928742) hydrogenase Fe-only [*Alistipes* sp. CAG:831] |  |
| P28_hydA282 | D4ZHLFP1:60:D2HDNACXX:2:1306:12764:45449:GGATGT | *Dictyoglomi*; *Dictyoglomales* | 79.3 | (YP_002352464) hydrogenase [*Dictyoglomus* *turgidum* DSM 6724] |  |
| P28_hydA20 | D4ZHLFP1:60:D2HDNACXX:2:2309:18606:31980:GGATGT | *Eukaryota*; *Viridiplantae*; *Chlorophyta* | 75.1 | (XP_001693376) Fe hydrogenase [*Chlamydomonas* *reinhardtii*] |  |
| P28_hydA344 | D4ZHLFP1:60:D2HDNACXX:2:2116:2593:55563:GGATGT | *Firmicutes*; *Bacilli*; *Lactobacillales* | 66.6 | (WP_010746116) [FeFe] hydrogenase, group A [*Enterococcus* *raffinosus*] |  |
| P28_hydA261 | D4ZHLFP1:60:D2HDNACXX:2:1308:14293:75233:GGATGT | *Firmicutes*; *Clostridia*; *Clostridiales* | 62.4 | (WP_004629896) hydrogenase, Fe-only [*RuminiClostridium*] |  |
| P28_hydA268 | D4ZHLFP1:60:D2HDNACXX:2:1113:11668:83184:GGATGT | *Firmicutes*; *Clostridia*; *Clostridiales* | 91.0 | (YP_001113004) hydrogenase [*Desulfotomaculum* *reducens* MI-1] |  |
| P28_hydA171 | D4ZHLFP1:60:D2HDNACXX:2:1109:1871:50152:GGATGT | *Firmicutes* | 63.5 | (WP_021856760) hydrogenase Fe only [*Firmicutes* bacterium CAG:555] |  |
| P28_hydA27 | D4ZHLFP1:60:D2HDNACXX:2:1114:17042:69653:GGATGT | *Firmicutes*; *Clostridia*; *Clostridiales* | 91.3 | (YP_001111834) hydrogenase [*Desulfotomaculum* *reducens* MI-1] |  |
| P28_hydA269 | D4ZHLFP1:60:D2HDNACXX:2:2106:11983:14451:GGATGT | *Firmicutes*; *Clostridia*; *Clostridiales* | 70.9 | (WP_018660435) Periplasmic [Fe] hydrogenase large subunit [Thermobrachium celere] |  |
| P28_hydA271 | D4ZHLFP1:60:D2HDNACXX:2:1105:3061:43800:GGATGT | *Firmicutes*; *Clostridia*; *Clostridiales* | 53.5 | (YP_003640841) hydrogenase, Fe-only [*Thermincola potens* JR] |  |
| P28_hydA272 | D4ZHLFP1:60:D2HDNACXX:2:2314:4574:35683:GGATGT | *Firmicutes*; *Clostridia*; *Clostridiales* | 70.5 | (WP_022278894) hydrogenase Fe-only [*Dorea* sp. AGR2135] |  |
| P28_hydA273 | D4ZHLFP1:60:D2HDNACXX:2:2310:14510:53518:GGATGT | *Firmicutes*; *Clostridia*; *Clostridiales* | 83.2 | (YP_004517644) hydrogenase, Fe-only [*Desulfotomaculum* *kuznetsovii* DSM 6115] |  |
| P28_hydA277 | D4ZHLFP1:60:D2HDNACXX:2:2115:13480:35918:GGATGT | *Firmicutes*; *Clostridia*; *Clostridiales* | 67.4 | (YP_004518295) hydrogenase, Fe-only [*Desulfotomaculum* *kuznetsovii* DSM 6115] |  |
| P28_hydA278 | D4ZHLFP1:60:D2HDNACXX:2:2110:3525:23785:GGATGT | *Firmicutes*; *Clostridia*; *Clostridiales* | 85.9 | (YP_004517644) hydrogenase, Fe-only [*Desulfotomaculum* *kuznetsovii* DSM 6115] |  |
| P28_hydA281 | D4ZHLFP1:60:D2HDNACXX:2:2107:7774:19512:GGATGT | *Firmicutes*; *Clostridia*; *Clostridiales* | 82.4 | (YP_004518295) hydrogenase, Fe-only [*Desulfotomaculum* *kuznetsovii* DSM 6115] |  |
| P28_hydA283 | D4ZHLFP1:60:D2HDNACXX:2:1303:11048:87799:GGATGT | *Firmicutes*; *Clostridia*; *Clostridiales* | 74.7 | (YP_004969732) hydrogenase, Fe-only [*Desulfosporosinus orientis* DSM 765] |  |
| P28_hydA287 | D4ZHLFP1:60:D2HDNACXX:2:1302:16282:18416:GGATGT | *Firmicutes*; *Clostridia*; *Clostridiales* | 57.0 | (YP_001113004) hydrogenase [*Desulfotomaculum* *reducens* MI-1] |  |
| P28_hydA294 | D4ZHLFP1:60:D2HDNACXX:2:2316:11710:32579:GGATGT | *Firmicutes*; *Clostridia*; *Clostridiales* | 79.3 | (WP_008410064) Iron hydrogenase 1 [*Desulfotomaculum* *hydrothermale*] |  |
| P28_hydA297 | D4ZHLFP1:60:D2HDNACXX:2:1106:18479:88233:GGATGT | *Firmicutes*; *Clostridia*; *Clostridiales* | 82.0 | (YP_003640841) hydrogenase, Fe-only [*Thermincola potens* JR] |  |
| P28_hydA298 | D4ZHLFP1:60:D2HDNACXX:2:2310:11693:54229:GGATGT | *Firmicutes*; *Clostridia*; *Clostridiales* | 82.0 | (YP_077035) Fe hydrogenase [*Symbiobacterium thermophilum* IAM 14863] |  |
| P28_hydA299 | D4ZHLFP1:60:D2HDNACXX:2:2113:15551:3692:GGATGT | *Firmicutes*; *Clostridia*; *Clostridiales* | 63.2 | (WP_018660435) Periplasmic [Fe] hydrogenase large subunit [*Thermobrachium* *celere*] |  |
| P28_hydA30 | D4ZHLFP1:60:D2HDNACXX:2:1303:3971:69538:GGATGT | *Firmicutes*; *Clostridia*; *Clostridiales* | 73.6 | (WP_021908252) hydrogenase Fe-only [*Eubacterium* sp. CAG:146] |  |
| P28_hydA301 | D4ZHLFP1:60:D2HDNACXX:2:2309:14190:70653:GGATGT | *Firmicutes*; *Clostridia*; *Clostridiales* | 80.1 | (YP_001113007) hydrogenase [*Desulfotomaculum* *reducens* MI-1] |  |
| P28_hydA303 | D4ZHLFP1:60:D2HDNACXX:2:2106:7935:41866:GGATGT | *Firmicutes*; *Clostridia*; *Clostridiales* | 87.0 | (YP_001212560) hydrogenase subunit [*Pelotomaculum* *thermopropionicum* SI] |  |
| P28_hydA304 | D4ZHLFP1:60:D2HDNACXX:2:2315:4334:79513:GGATGT | *Firmicutes*; *Clostridia*; *Clostridiales* | 84.7 | (YP_004517644) hydrogenase, Fe-only [*Desulfotomaculum* *kuznetsovii* DSM 6115] |  |
| P28_hydA305 | D4ZHLFP1:60:D2HDNACXX:2:2309:12156:27025:GGATGT | *Firmicutes*; *Clostridia*; *Clostridiales* | 90.9 | (YP_004517644) hydrogenase, Fe-only [*Desulfotomaculum* *kuznetsovii* DSM 6115] |  |
| P28_hydA306 | D4ZHLFP1:60:D2HDNACXX:2:2313:10990:69037:GGATGT | *Firmicutes*; *Clostridia*; *Clostridiales* | 95.5 | (YP_004545921) hydrogenase Fe-only [*Desulfotomaculum* *ruminis* DSM 2154] |  |
| P28_hydA307 | D4ZHLFP1:60:D2HDNACXX:2:2308:16669:82442:GGATGT | *Firmicutes*; *Clostridia*; *Clostridiales* | 89.4 | (WP_008410059) Iron hydrogenase 1 [*Desulfotomaculum* *hydrothermale*] |  |
| P28_hydA309 | D4ZHLFP1:60:D2HDNACXX:2:1105:7395:56887:GGATGT | *Firmicutes*; *Clostridia*; *Clostridiales* | 72.4 | (GAE88236) hydrogenase [*Clostridium* *straminisolvens* JCM 21531] |  |
| P28_hydA310 | D4ZHLFP1:60:D2HDNACXX:2:2306:18542:41819:GGATGT | *Firmicutes*; *Clostridia*; *Clostridiales* | 51.2 | (YP_003935869) Periplasmic [Fe] hydrogenase large subunit [*Clostridium* *sticklandii*] |  |
| P28_hydA311 | D4ZHLFP1:60:D2HDNACXX:2:2306:18542:41819:GGATGT | *Firmicutes*; *Clostridia*; *Clostridiales* | 59.3 | (WP_022334732) hydrogenase Fe-only [*Clostridium* sp. CAG:452] |  |
| P28_hydA314 | D4ZHLFP1:60:D2HDNACXX:2:2115:15661:25130:GGATGT | *Firmicutes*; *Clostridia*; *Clostridiales* | 75.1 | (YP_001212560) hydrogenase subunit [*Pelotomaculum* *thermopropionicum* SI] |  |
| P28_hydA321 | D4ZHLFP1:60:D2HDNACXX:2:1314:6888:24210:GGATGT | *Firmicutes*; *Clostridia*; *Clostridiales* | 69.3 | (YP_004518295) hydrogenase, Fe-only [*Desulfotomaculum* *kuznetsovii* DSM 6115] |  |
| P28_hydA323 | D4ZHLFP1:60:D2HDNACXX:2:2311:18724:30480:GGATGT | *Firmicutes*; *Clostridia*; *Clostridiales* | 70.9 | (YP_004496996) hydrogenase, Fe-only [*Desulfotomaculum* *carboxydivorans* CO-1-SRB] |  |
| P28_hydA324 | D4ZHLFP1:60:D2HDNACXX:2:1108:14667:21673:GGATGT | *Firmicutes*; *Clostridia*; *Clostridiales* | 84.7 | (YP_003189750) hydrogenase, Fe-only [*Desulfotomaculum* *acetoxidans* DSM 771] |  |
| P28_hydA325 | D4ZHLFP1:60:D2HDNACXX:2:2115:7933:6640:GGATGT | *Firmicutes*; *Clostridia*; *Clostridiales* | 89.4 | (WP_021944433) hydrogenase Fe-only [*Clostridium* sp. CAG:264] |  |
| P28_hydA329 | D4ZHLFP1:60:D2HDNACXX:2:2306:5267:3583:GGATGT | *Firmicutes*; *Clostridia*; *Clostridiales* | 68.9 | (YP_004517644) hydrogenase, Fe-only [*Desulfotomaculum* *kuznetsovii* DSM 6115] |  |
| P28_hydA330 | D4ZHLFP1:60:D2HDNACXX:2:1108:10790:47267:GGATGT | *Firmicutes*; *Clostridia*; *Clostridiales* | 98.0 | (YP_004517644) hydrogenase, Fe-only [*Desulfotomaculum* *kuznetsovii* DSM 6115] |  |
| P28_hydA333 | D4ZHLFP1:60:D2HDNACXX:2:2304:19319:89346:GGATGT | *Firmicutes*; *Clostridia*; *Clostridiales* | 75.9 | (WP_023062885) hydrogenase, Fe-only [*Clostridium* *thermocellum*] |  |
| P28_hydA334 | D4ZHLFP1:60:D2HDNACXX:2:2309:8979:10824:GGATGT | *Firmicutes*; *Clostridia*; *Clostridiales* | 72.8 | (WP_002575948) [FeFe] hydrogenase, group A [Lachno*Clostridium*] |  |
| P28_hydA338 | D4ZHLFP1:60:D2HDNACXX:2:1111:11445:80970:GGATGT | *Firmicutes*; *Clostridia*; *Clostridiales* | 78.6 | (WP_023055471) [FeFe] hydrogenase, group A [*Peptoniphilus* sp. BV3C26] |  |
| P28_hydA339 | D4ZHLFP1:60:D2HDNACXX:2:1313:16269:20520:GGATGT | *Firmicutes*; *Clostridia*; *Clostridiales* | 66.2 | (WP_021631077) putative ferredoxin hydrogenase HydA1 [*Clostridium* sp. ATCC BAA-442] |  |
| P28_hydA340 | D4ZHLFP1:60:D2HDNACXX:2:1313:20645:57963:GGATGT | *Firmicutes*; *Clostridia*; *Clostridiales* | 84.3 | (WP_008410064) Iron hydrogenase 1 [*Desulfotomaculum* *hydrothermale*] |  |
| P28_hydA348 | D4ZHLFP1:60:D2HDNACXX:2:1308:18829:65204:GGATGT | *Firmicutes*; *Clostridia*; *Clostridiales* | 99.8 | (YP_004517644) hydrogenase, Fe-only [*Desulfotomaculum* *kuznetsovii* DSM 6115] |  |
| P28_hydA352 | D4ZHLFP1:60:D2HDNACXX:2:2312:20149:17973:GGATGT | *Firmicutes*; *Clostridia*; *Clostridiales* | 58.2 | (YP_001717478) hydrogenase, Fe-only [*Candidatus* *Desulforudis* *audaxviator* MP104C] |  |
| P28_hydA355 | D4ZHLFP1:60:D2HDNACXX:2:1108:1063:76207:GGATGT | *Firmicutes*; *Clostridia*; *Clostridiales* | 92.0 | (YP_004517644) hydrogenase, Fe-only [*Desulfotomaculum* *kuznetsovii* DSM 6115] |  |
| P28_hydA358 | D4ZHLFP1:60:D2HDNACXX:2:2313:12476:75649:GGATGT | *Firmicutes*; *Clostridia*; *Clostridiales* | 75.5 | (YP_004517644) hydrogenase, Fe-only [*Desulfotomaculum* *kuznetsovii* DSM 6115] |  |
| P28_hydA362 | D4ZHLFP1:60:D2HDNACXX:2:2104:11024:30939:GGATGT | *Firmicutes*; *Clostridia*; *Clostridiales* | 91.3 | (YP_004545925) hydrogenase Fe-only [*Desulfotomaculum* *ruminis* DSM 2154] |  |
| P28_hydA364 | D4ZHLFP1:60:D2HDNACXX:2:2312:11597:12789:GGATGT | *Firmicutes*; *Clostridia*; *Clostridiales* | 79.0 | (YP_004092838) hydrogenase, Fe-only [*Ethanoligenens* *harbinense* YUAN-3] |  |
| P28_hydA365 | D4ZHLFP1:60:D2HDNACXX:2:2308:6036:12495:GGATGT | *Firmicutes*; *Clostridia*; *Clostridiales* | 80.1 | (YP_001113007) hydrogenase [*Desulfotomaculum* *reducens* MI-1] |  |
| P28_hydA366 | D4ZHLFP1:60:D2HDNACXX:2:2311:7920:97575:GGATGT | *Firmicutes*; *Clostridia*; *Clostridiales* | 69.3 | (YP_004517644) hydrogenase, Fe-only [*Desulfotomaculum* *kuznetsovii* DSM 6115] |  |
| P28_hydA367 | D4ZHLFP1:60:D2HDNACXX:2:2314:15145:33230:GGATGT | *Firmicutes*; *Clostridia*; *Clostridiales* | 88.2 | (WP_008410059) Iron hydrogenase 1 [*Desulfotomaculum* *hydrothermale*] |  |
| P28_hydA368 | D4ZHLFP1:60:D2HDNACXX:2:2109:15313:70001:GGATGT | *Firmicutes*; *Clostridia*; *Clostridiales* | 88.6 | (WP_008410059) Iron hydrogenase 1 [*Desulfotomaculum* *hydrothermale*] |  |
| P28_hydA369 | D4ZHLFP1:60:D2HDNACXX:2:1106:17580:24317:GGATGT | *Firmicutes*; *Clostridia*; *Clostridiales* | 75.5 | (YP_001212560) hydrogenase subunit [*Pelotomaculum* *thermopropionicum* SI] |  |
| P28_hydA370 | D4ZHLFP1:60:D2HDNACXX:2:2116:20310:44187:GGATGT | *Firmicutes*; *Clostridia*; *Clostridiales* | 94.7 | (YP_001212560) hydrogenase subunit [*Pelotomaculum* *thermopropionicum* SI] |  |
| P28_hydA375 | D4ZHLFP1:60:D2HDNACXX:2:2107:16775:45547:GGATGT | *Firmicutes*; *Clostridia*; *Clostridiales* | 78.2 | (YP_004517644) hydrogenase, Fe-only [*Desulfotomaculum* *kuznetsovii* DSM 6115] |  |
| P28_hydA378 | D4ZHLFP1:60:D2HDNACXX:2:1313:18604:67031:GGATGT | *Firmicutes*; *Clostridia*; *Clostridiales* | 54.7 | (YP_001113004) hydrogenase [*Desulfotomaculum* *reducens* MI-1] |  |
| P28_hydA379 | D4ZHLFP1:60:D2HDNACXX:2:2310:9758:40457:GGATGT | *Firmicutes*; *Clostridia*; *Clostridiales* | 72.4 | (YP_004518295) hydrogenase, Fe-only [*Desulfotomaculum* *kuznetsovii* DSM 6115] |  |
| P28_hydA38 | D4ZHLFP1:60:D2HDNACXX:2:1310:3830:87646:GGATGT | *Firmicutes*; *Clostridia*; *Clostridiales* | 90.5 | (YP_004496996) hydrogenase, Fe-only [*Desulfotomaculum* *carboxydivorans* CO-1-SRB] |  |
| P28_hydA382 | D4ZHLFP1:60:D2HDNACXX:2:1110:6113:88488:GGATGT | *Firmicutes*; *Clostridia*; *Clostridiales* | 86.7 | (YP_007946226) hydrogenase, Fe-only [*Desulfotomaculum* *gibsoniae* DSM 7213] |  |
| P28_hydA390 | D4ZHLFP1:60:D2HDNACXX:2:1303:16851:39485:GGATGT | *Firmicutes*; *Clostridia*; *Clostridiales* | 84.0 | (YP_004517644) hydrogenase, Fe-only [*Desulfotomaculum* *kuznetsovii* DSM 6115] |  |
| P28_hydA392 | D4ZHLFP1:60:D2HDNACXX:2:2105:4564:41998:GGATGT | *Firmicutes*; *Clostridia*; *Clostridiales* | 91.0 | (YP_001717478) hydrogenase, Fe-only [*Candidatus* *Desulforudis* *audaxviator* MP104C] |  |
| P28_hydA394 | D4ZHLFP1:60:D2HDNACXX:2:1311:9862:93498:GGATGT | *Firmicutes*; *Clostridia*; *Clostridiales* | 94.4 | (YP_007946226) hydrogenase, Fe-only [*Desulfotomaculum* *gibsoniae* DSM 7213] |  |
| P28_hydA395 | D4ZHLFP1:60:D2HDNACXX:2:1315:19794:29264:GGATGT | *Firmicutes*; *Clostridia*; *Clostridiales* | 72.4 | (YP_004497002) hydrogenase, Fe-only [*Desulfotomaculum* *carboxydivorans* CO-1-SRB] |  |
| P28_hydA399 | D4ZHLFP1:60:D2HDNACXX:2:2314:6545:58167:GGATGT | *Firmicutes*; *Clostridia*; *Clostridiales* | 67.8 | (YP_001036861) hydrogenase, Fe-only [*Clostridium* *thermocellum* ATCC 27405] |  |
| P28_hydA4 | D4ZHLFP1:60:D2HDNACXX:2:1303:7759:53542:GGATGT | *Firmicutes*; *Clostridia*; *Clostridiales* | 91.3 | (YP_001111834) hydrogenase [*Desulfotomaculum* *reducens* MI-1] |  |
| P28_hydA40 | D4ZHLFP1:60:D2HDNACXX:2:1306:1645:61729:GGATGT | *Firmicutes*; *Clostridia*; *Clostridiales* | 87.8 | (WP_008410059) Iron hydrogenase 1 [*Desulfotomaculum* *hydrothermale*] |  |
| P28_hydA400 | D4ZHLFP1:60:D2HDNACXX:2:1309:6420:91463:GGATGT | *Firmicutes*; *Clostridia*; *Clostridiales* | 82.8 | (YP_001212560) hydrogenase subunit [Pelotomaculum thermopropionicum SI] |  |
| P28_hydA404 | D4ZHLFP1:60:D2HDNACXX:2:2102:9403:88261:GGATGT | *Firmicutes*; *Clostridia*; *Clostridiales* | 61.2 | (YP_004518238) hydrogenase, Fe-only [*Desulfotomaculum* *kuznetsovii* DSM 6115] |  |
| P28_hydA405 | D4ZHLFP1:60:D2HDNACXX:2:2112:4472:70529:GGATGT | *Firmicutes*; *Clostridia*; *Clostridiales* | 66.6 | (WP_023055471) [FeFe] hydrogenase, group A [*Peptoniphilus* sp. BV3C26] |  |
| P28_hydA406 | D4ZHLFP1:60:D2HDNACXX:2:2105:5939:33002:GGATGT | *Firmicutes*; *Clostridia*; *Clostridiales* | 92.4 | (YP_001113007) hydrogenase [*Desulfotomaculum* *reducens* MI-1] |  |
| P28_hydA408 | D4ZHLFP1:60:D2HDNACXX:2:2314:12785:56856:GGATGT | *Firmicutes*; *Clostridia*; *Clostridiales* | 73.9 | (YP_004496996) hydrogenase, Fe-only [*Desulfotomaculum* *carboxydivorans* CO-1-SRB] |  |
| P28_hydA41 | D4ZHLFP1:60:D2HDNACXX:2:1115:4212:75038:GGATGT | *Firmicutes*; *Clostridia*; *Clostridiales* | 80.5 | (YP_004517644) hydrogenase, Fe-only [*Desulfotomaculum* *kuznetsovii* DSM 6115] |  |
| P28_hydA413 | D4ZHLFP1:60:D2HDNACXX:2:1315:19383:15464:GGATGT | *Firmicutes*; *Clostridia*; *Clostridiales* | 86.7 | (YP_004518295) hydrogenase, Fe-only [*Desulfotomaculum* *kuznetsovii* DSM 6115] |  |
| P28_hydA42 | D4ZHLFP1:60:D2HDNACXX:2:1303:12690:67984:GGATGT | *Firmicutes*; *Clostridia*; *Clostridiales* | 74.7 | (YP_001113004) hydrogenase [*Desulfotomaculum* *reducens* MI-1] |  |
| P28_hydA423 | D4ZHLFP1:60:D2HDNACXX:2:2302:5669:10810:GGATGT | *Firmicutes*; *Clostridia*; *Clostridiales* | 83.2 | (WP_021908252) hydrogenase Fe-only [*Eubacterium* sp. CAG:146] |  |
| P28_hydA427 | D4ZHLFP1:60:D2HDNACXX:2:2304:15003:48833:GGATGT | *Firmicutes*; *Clostridia*; *Clostridiales* | 98.6 | (YP_001716352) hydrogenase, Fe-only [*Candidatus* *Desulforudis* *audaxviator* MP104C] |  |
| P28_hydA429 | D4ZHLFP1:60:D2HDNACXX:2:2110:8435:29603:GGATGT | *Firmicutes*; *Clostridia*; *Clostridiales* | 78.6 | (YP_004518295) hydrogenase, Fe-only [*Desulfotomaculum* *kuznetsovii* DSM 6115] |  |
| P28_hydA43 | D4ZHLFP1:60:D2HDNACXX:2:1106:16600:90499:GGATGT | *Firmicutes*; *Clostridia*; *Clostridiales* | 81.6 | (YP_007946226) hydrogenase, Fe-only [*Desulfotomaculum* *gibsoniae* DSM 7213] |  |
| P28_hydA432 | D4ZHLFP1:60:D2HDNACXX:2:1113:6864:54911:GGATGT | *Firmicutes*; *Clostridia*; *Clostridiales* | 79.3 | (YP_004517644) hydrogenase, Fe-only [*Desulfotomaculum* *kuznetsovii* DSM 6115] |  |
| P28_hydA435 | D4ZHLFP1:60:D2HDNACXX:2:2308:11447:88223:GGATGT | *Firmicutes*; *Clostridia*; *Clostridiales* | 52.4 | (YP_005045889) hydrogenase, Fe-only [*Clostridium* *clariflavum* DSM 19732] |  |
| P28_hydA437 | D4ZHLFP1:60:D2HDNACXX:2:2316:19962:48015:GGATGT | *Firmicutes*; *Clostridia*; *Clostridiales* | 69.3 | (YP_004545925) hydrogenase Fe-only [*Desulfotomaculum* *ruminis* DSM 2154] |  |
| P28_hydA442 | D4ZHLFP1:60:D2HDNACXX:2:1301:9253:62978:GGATGT | *Firmicutes*; *Clostridia*; *Clostridiales* | 77.4 | (YP_001113007) hydrogenase [*Desulfotomaculum* *reducens* MI-1] |  |
| P28_hydA449 | D4ZHLFP1:60:D2HDNACXX:2:1314:2209:70843:GGATGT | *Firmicutes*; *Clostridia*; *Clostridiales* | 75.5 | (YP_007946226) hydrogenase, Fe-only [*Desulfotomaculum* *gibsoniae* DSM 7213] |  |
| P28_hydA450 | D4ZHLFP1:60:D2HDNACXX:2:1309:2955:95343:GGATGT | *Firmicutes*; *Clostridia*; *Clostridiales* | 73.2 | (YP_002506620) hydrogenase, Fe-only [*Clostridium* cellulolyticum H10] |  |
| P28_hydA453 | D4ZHLFP1:60:D2HDNACXX:2:2301:13500:63367:GGATGT | *Firmicutes*; *Clostridia*; *Clostridiales* | 65.1 | (WP_008373638) hydrogenase [*Coprococcus* *comes*] |  |
| P28_hydA454 | D4ZHLFP1:60:D2HDNACXX:2:1310:12419:73074:GGATGT | *Firmicutes*; *Clostridia*; *Clostridiales* | 82.8 | (YP_007946226) hydrogenase, Fe-only [*Desulfotomaculum* *gibsoniae* DSM 7213] | |
| P28_hydA459 | D4ZHLFP1:60:D2HDNACXX:2:1314:17708:39938:GGATGT | *Firmicutes*; *Clostridia*; *Clostridiales* | 80.9 | (YP_004518295) hydrogenase, Fe-only [*Desulfotomaculum* *kuznetsovii* DSM 6115] |  |
| P28_hydA46 | D4ZHLFP1:60:D2HDNACXX:2:1101:16067:94997:GGATGT | *Firmicutes*; *Clostridia*; *Clostridiales* | 92.4 | (WP_008410064) Iron hydrogenase 1 [*Desulfotomaculum* *hydrothermale*] |  |
| P28_hydA461 | D4ZHLFP1:60:D2HDNACXX:2:2306:11974:33002:GGATGT | *Firmicutes*; *Clostridia*; *Clostridiales* | 87.8 | (YP_004545921) hydrogenase Fe-only [*Desulfotomaculum* *ruminis* DSM 2154] |  |
| P28_hydA462 | D4ZHLFP1:60:D2HDNACXX:2:1315:2857:77555:GGATGT | *Firmicutes*; *Clostridia*; *Clostridiales* | 72.0 | (YP_008699055) hydrogenase, Fe-only [*Clostridium* *autoethanogenum* DSM 10061] |  |
| P28_hydA463 | D4ZHLFP1:60:D2HDNACXX:2:2109:17068:70952:GGATGT | *Firmicutes*; *Clostridia*; *Clostridiales* | 53.9 | (WP_010244148) hydrogenase [*Acetivibrio* *cellulolyticus*] |  |
| P28_hydA464 | D4ZHLFP1:60:D2HDNACXX:2:2308:9561:5891:GGATGT | *Firmicutes*; *Clostridia*; *Clostridiales* | 74.3 | (YP_004518295) hydrogenase, Fe-only [*Desulfotomaculum* *kuznetsovii* DSM 6115] |  |
| P28_hydA465 | D4ZHLFP1:60:D2HDNACXX:2:2303:8752:77532:GGATGT | *Firmicutes*; *Clostridia*; *Clostridiales* | 93.2 | (YP_003189750) hydrogenase, Fe-only [*Desulfotomaculum* *acetoxidans* DSM 771] |  |
| P28_hydA467 | D4ZHLFP1:60:D2HDNACXX:2:2108:12294:80384:GGATGT | *Firmicutes*; *Clostridia*; *Clostridiales* | 75.5 | (YP_077035) iron hydrogenase [*Symbiobacterium thermophilum* IAM 14863] |  |
| P28_hydA468 | D4ZHLFP1:60:D2HDNACXX:2:1309:18520:11621:GGATGT | *Firmicutes*; *Clostridia*; *Clostridiales* | 98.2 | (YP_001212560) hydrogenase subunit [*Pelotomaculum* *thermopropionicum* SI] |  |
| P28_hydA471 | D4ZHLFP1:60:D2HDNACXX:2:1308:8193:42336:GGATGT | *Firmicutes*; *Clostridia*; *Clostridiales* | 99.0 | (YP_001716352) hydrogenase, Fe-only [*Candidatus* *Desulforudis* *audaxviator* MP104C] |  |
| P28_hydA5 | D4ZHLFP1:60:D2HDNACXX:2:2313:8363:60911:GGATGT | *Firmicutes*; *Clostridia*; *Clostridiales* | 83.2 | (YP_004518295) hydrogenase, Fe-only [*Desulfotomaculum* *kuznetsovii* DSM 6115] |  |
| P28_hydA50 | D4ZHLFP1:60:D2HDNACXX:2:1113:11592:79933:GGATGT | *Firmicutes*; *Clostridia*; *Clostridiales* | 82.0 | (YP_004517644) hydrogenase, Fe-only [*Desulfotomaculum* *kuznetsovii* DSM 6115] |  |
| P28_hydA51 | D4ZHLFP1:60:D2HDNACXX:2:2316:12094:16372:GGATGT | *Firmicutes*; *Clostridia*; *Clostridiales* | 74.7 | (YP_004518295) hydrogenase, Fe-only [*Desulfotomaculum* *kuznetsovii* DSM 6115] |  |
| P28_hydA56 | D4ZHLFP1:60:D2HDNACXX:2:1116:9777:2323:GGATGT | *Firmicutes*; *Clostridia*; *Clostridiales* | 75.5 | (WP_022071136) hydrogenase Fe-only [*Clostridium* *bartlettii* CAG:1329] |  |
| P28_hydA66 | D4ZHLFP1:60:D2HDNACXX:2:2106:4911:95193:GGATGT | *Firmicutes*; *Clostridia*; *Clostridiales* | 81.3 | (WP_008410059) Iron hydrogenase 1 [*Desulfotomaculum* *hydrothermale*] |  |
| P28_hydA69 | D4ZHLFP1:60:D2HDNACXX:2:2304:12327:89856:GGATGT | *Firmicutes*; *Clostridia*; *Clostridiales* | 82.8 | (YP_001113004) hydrogenase [*Desulfotomaculum* *reducens* MI-1] |  |
| P28_hydA73 | D4ZHLFP1:60:D2HDNACXX:2:2303:6298:51820:GGATGT | *Firmicutes*; *Clostridia*; *Clostridiales* | 61.2 | (YP_004517644) hydrogenase, Fe-only [*Desulfotomaculum* *kuznetsovii* DSM 6115] |  |
| P28_hydA75 | D4ZHLFP1:60:D2HDNACXX:2:1102:16085:5082:GGATGT | *Firmicutes*; *Clostridia*; *Clostridiales* | 70.5 | (YP_003640841) hydrogenase, Fe-only [*Thermincola potens* JR] |  |
| P28_hydA76 | D4ZHLFP1:60:D2HDNACXX:2:1113:5070:77194:GGATGT | *Firmicutes*; *Clostridia*; *Clostridiales* | 80.9 | (YP_004498193) hydrogenase, Fe-only [*Desulfotomaculum* *carboxydivorans* CO-1-SRB] |  |
| P28_hydA79 | D4ZHLFP1:60:D2HDNACXX:2:2110:14682:4653:GGATGT | *Firmicutes*; *Clostridia*; *Clostridiales* | 82.4 | (YP_004517644) hydrogenase, Fe-only [*Desulfotomaculum* *kuznetsovii* DSM 6115] |  |
| P28_hydA8 | D4ZHLFP1:60:D2HDNACXX:2:2105:9592:29920:GGATGT | *Firmicutes*; *Clostridia*; *Clostridiales* | 80.5 | (YP_001113004) hydrogenase [*Desulfotomaculum* *reducens* MI-1] |  |
| P28_hydA84 | D4ZHLFP1:60:D2HDNACXX:2:2114:2666:53657:GGATGT | *Firmicutes*; *Clostridia*; *Clostridiales* | 75.5 | (YP_008699055) hydrogenase, Fe-only [*Clostridium* *autoethanogenum* DSM 10061] |  |
| P28_hydA85 | D4ZHLFP1:60:D2HDNACXX:2:2109:19628:92874:GGATGT | *Firmicutes*; *Clostridia*; *Clostridiales* | 77.0 | (YP_001212560) hydrogenase subunit [*Pelotomaculum* *thermopropionicum* SI] |  |
| P28_hydA86 | D4ZHLFP1:60:D2HDNACXX:2:1308:8109:90227:GGATGT | *Firmicutes*; *Clostridia*; *Clostridiales* | 83.2 | (YP_004518295) hydrogenase, Fe-only [*Desulfotomaculum* *kuznetsovii* DSM 6115] |  |
| P28_hydA88 | D4ZHLFP1:60:D2HDNACXX:2:1303:11762:71086:GGATGT | *Firmicutes*; *Clostridia*; *Clostridiales* | 50.8 | (YP_003640841) hydrogenase, Fe-only [*Thermincola potens* JR] |  |
| P28_hydA9 | D4ZHLFP1:60:D2HDNACXX:2:1116:4572:23493:GGATGT | *Firmicutes*; *Clostridia*; *Clostridiales* | 63.5 | (YP_004496996) hydrogenase, Fe-only [*Desulfotomaculum* *carboxydivorans* CO-1-SRB] |  |
| P28_hydA91 | D4ZHLFP1:60:D2HDNACXX:2:1306:14960:38560:GGATGT | *Firmicutes*; *Clostridia*; *Clostridiales* | 54.7 | (YP_001113004) hydrogenase [*Desulfotomaculum* *reducens* MI-1] |  |
| P28_hydA95 | D4ZHLFP1:60:D2HDNACXX:2:1301:13034:98797:GGATGT | *Firmicutes*; *Clostridia*; *Clostridiales* | 97.0 | (YP_001717478) hydrogenase, Fe-only [*Candidatus* *Desulforudis* *audaxviator* MP104C] |  |
| P28_hydA11 | D4ZHLFP1:60:D2HDNACXX:2:1311:11947:31607:GGATGT | *Firmicutes*; *Clostridia*; *Clostridiales* | 70.5 | (YP_003640841) hydrogenase, Fe-only [*Thermincola potens* JR] |  |
| P28_hydA113 | D4ZHLFP1:60:D2HDNACXX:2:1315:19200:83174:GGATGT | *Firmicutes*; *Clostridia*; *Clostridiales* | 97.1 | (YP_003189750) hydrogenase, Fe-only [*Desulfotomaculum* *acetoxidans* DSM 771] |  |
| P28_hydA116 | D4ZHLFP1:60:D2HDNACXX:2:2306:11088:25324:GGATGT | *Firmicutes*; *Clostridia*; *Clostridiales* | 93.6 | (WP_008410059) Iron hydrogenase 1 [*Desulfotomaculum* *hydrothermale*] |  |
| P28_hydA117 | D4ZHLFP1:60:D2HDNACXX:2:2116:3269:29850:GGATGT | *Firmicutes*; *Clostridia*; *Clostridiales* | 89.7 | (YP_004545921) hydrogenase Fe-only [*Desulfotomaculum* *ruminis* DSM 2154] |  |
| P28_hydA121 | D4ZHLFP1:60:D2HDNACXX:2:2313:10137:45526:GGATGT | *Firmicutes*; *Clostridia*; *Clostridiales* | 90.8 | (YP_004517644) hydrogenase, Fe-only [*Desulfotomaculum* *kuznetsovii* DSM 6115] |  |
| P28_hydA123 | D4ZHLFP1:60:D2HDNACXX:2:2107:16945:52378:GGATGT | *Firmicutes*; *Clostridia*; *Clostridiales* | 91.3 | (YP_004518295) hydrogenase, Fe-only [*Desulfotomaculum* *kuznetsovii* DSM 6115] |  |
| P28_hydA124 | D4ZHLFP1:60:D2HDNACXX:2:1112:15984:85021:GGATGT | *Firmicutes*; *Clostridia*; *Clostridiales* | 89.4 | (YP_003189750) hydrogenase, Fe-only [*Desulfotomaculum* *acetoxidans* DSM 771] |  |
| P28_hydA126 | D4ZHLFP1:60:D2HDNACXX:2:1101:12401:26344:GGATGT | *Firmicutes*; *Clostridia*; *Clostridiales* | 93.6 | (YP_004518295) hydrogenase, Fe-only [*Desulfotomaculum* *kuznetsovii* DSM 6115] |  |
| P28_hydA127 | D4ZHLFP1:60:D2HDNACXX:2:2114:18376:91305:GGATGT | *Firmicutes*; *Clostridia*; *Clostridiales* | 85.1 | (YP_004497002) hydrogenase, Fe-only [*Desulfotomaculum* *carboxydivorans* CO-1-SRB] |  |
| P28_hydA130 | D4ZHLFP1:60:D2HDNACXX:2:2111:20908:32283:GGATGT | *Firmicutes*; *Clostridia*; *Clostridiales* | 89.0 | (WP_008410059) Iron hydrogenase 1 [*Desulfotomaculum* *hydrothermale*] |  |
| P28_hydA134 | D4ZHLFP1:60:D2HDNACXX:2:1114:16202:5057:GGATGT | *Firmicutes*; *Clostridia*; *Clostridiales* | 69.7 | (YP_004517644) hydrogenase, Fe-only [*Desulfotomaculum* *kuznetsovii* DSM 6115] |  |
| P28_hydA137 | D4ZHLFP1:60:D2HDNACXX:2:2102:3729:17264:GGATGT | *Firmicutes*; *Clostridia*; *Clostridiales* | 79.3 | (YP_001212560) hydrogenase subunit [*Pelotomaculum* *thermopropionicum* SI] |  |
| P28_hydA139 | D4ZHLFP1:60:D2HDNACXX:2:1108:11575:34060:GGATGT | *Firmicutes*; *Clostridia*; *Clostridiales* | 66.2 | (WP_023062885) hydrogenase, Fe-only [*Clostridium* *thermocellum*] |  |
| P28_hydA14 | D4ZHLFP1:60:D2HDNACXX:2:1304:3003:11855:GGATGT | *Firmicutes*; *Clostridia*; *Clostridiales* | 77.0 | (YP_004969732) hydrogenase, Fe-only [*Desulfosporosinus orientis* DSM 765] |  |
| P28_hydA144 | D4ZHLFP1:60:D2HDNACXX:2:2316:10303:37616:GGATGT | *Firmicutes*; *Clostridia*; *Clostridiales* | 90.1 | (YP_001113007) hydrogenase [*Desulfotomaculum* *reducens* MI-1] |  |
| P28_hydA145 | D4ZHLFP1:60:D2HDNACXX:2:2310:1848:85387:GGATGT | *Firmicutes*; *Clostridia*; *Clostridiales* | 78.6 | (YP_001212560) hydrogenase subunit [*Pelotomaculum* *thermopropionicum* SI] |  |
| P28_hydA146 | D4ZHLFP1:60:D2HDNACXX:2:2304:4583:85850:GGATGT | *Firmicutes*; *Clostridia*; *Clostridiales* | 87.4 | (YP_004545921) hydrogenase Fe-only [*Desulfotomaculum* *ruminis* DSM 2154] |  |
| P28_hydA147 | D4ZHLFP1:60:D2HDNACXX:2:2108:19118:60589:GGATGT | *Firmicutes*; *Clostridia*; *Clostridiales* | 84.3 | (WP_022038410) hydrogenase Fe-only [*Ruminococcus* *gnavus* CAG:126] |  |
| P28_hydA150 | D4ZHLFP1:60:D2HDNACXX:2:2303:14872:83867:GGATGT | *Firmicutes*; *Clostridia*; *Clostridiales* | 82.4 | (YP_001716352) hydrogenase, Fe-only [*Candidatus* *Desulforudis* *audaxviator* MP104C] | |
| P28_hydA152 | D4ZHLFP1:60:D2HDNACXX:2:1316:19452:70846:GGATGT | *Firmicutes*; *Clostridia*; *Clostridiales* | 74.7 | (YP_004518295) hydrogenase, Fe-only [*Desulfotomaculum* *kuznetsovii* DSM 6115] |  |
| P28_hydA160 | D4ZHLFP1:60:D2HDNACXX:2:2111:19543:12745:GGATGT | *Firmicutes*; *Clostridia*; *Clostridiales* | 75.9 | (WP_023055471) [FeFe] hydrogenase, group A [*Peptoniphilus* sp. BV3C26] |  |
| P28_hydA162 | D4ZHLFP1:60:D2HDNACXX:2:1311:8325:90007:GGATGT | *Firmicutes*; *Clostridia*; *Clostridiales* | 85.9 | (WP_008410059) Iron hydrogenase 1 [*Desulfotomaculum* *hydrothermale*] |  |
| P28_hydA165 | D4ZHLFP1:60:D2HDNACXX:2:1315:17668:96907:GGATGT | *Firmicutes*; *Clostridia*; *Clostridiales* | 88.6 | (YP_003828850) NAD(P)-dependent iron-only hydrogenase [*Acetohalobium arabaticum* DSM 5501] |  |
| P28_hydA166 | D4ZHLFP1:60:D2HDNACXX:2:1304:14131:84579:GGATGT | *Firmicutes*; *Clostridia*; *Clostridiales* | 76.6 | (YP_007946226) hydrogenase, Fe-only [*Desulfotomaculum* *gibsoniae* DSM 7213] |  |
| P28_hydA167 | D4ZHLFP1:60:D2HDNACXX:2:1306:17917:68044:GGATGT | *Firmicutes*; *Clostridia*; *Clostridiales* | 57.4 | (WP_023062885) hydrogenase, Fe-only [*Clostridium* *thermocellum*] |  |
| P28_hydA168 | D4ZHLFP1:60:D2HDNACXX:2:1312:6724:38486:GGATGT | *Firmicutes*; *Clostridia*; *Clostridiales* | 92.0 | (YP_004518238) hydrogenase, Fe-only [*Desulfotomaculum* *kuznetsovii* DSM 6115] |  |
| P28_hydA17 | D4ZHLFP1:60:D2HDNACXX:2:2114:16346:87258:GGATGT | *Firmicutes*; *Clostridia*; *Clostridiales* | 77.4 | (YP_004545921) hydrogenase Fe-only [*Desulfotomaculum* *ruminis* DSM 2154] |  |
| P28_hydA172 | D4ZHLFP1:60:D2HDNACXX:2:1104:17994:78933:GGATGT | *Firmicutes*; *Clostridia*; *Clostridiales* | 82.8 | (WP_022288920) putative uptake hydrogenase subunit HupA [*Oscillibacter* sp. CAG:155] |  |
| P28_hydA173 | D4ZHLFP1:60:D2HDNACXX:2:2316:17949:94326:GGATGT | *Firmicutes*; *Clostridia*; *Clostridiales* | 81.6 | (WP_007715799) hydrogenase [*Clostridium* *asparagiforme*] |  |
| P28_hydA175 | D4ZHLFP1:60:D2HDNACXX:2:2316:18562:77270:GGATGT | *Firmicutes*; *Clostridia*; *Clostridiales* | 88.2 | (YP_004092838) hydrogenase, Fe-only [Ethanoligenens harbinense YUAN-3] |  |
| P28_hydA178 | D4ZHLFP1:60:D2HDNACXX:2:1108:17079:23070:GGATGT | *Firmicutes*; *Clostridia*; *Clostridiales* | 94.7 | (YP_003189750) hydrogenase, Fe-only [*Desulfotomaculum* *acetoxidans* DSM 771] |  |
| P28_hydA18 | D4ZHLFP1:60:D2HDNACXX:2:1116:9957:88271:GGATGT | *Firmicutes*; *Clostridia*; *Clostridiales* | 68.6 | (YP_001318525) hydrogenase, Fe-only [*Alkaliphilus* *metalliredigens* QYMF] |  |
| P28_hydA188 | D4ZHLFP1:60:D2HDNACXX:2:1110:10828:80546:GGATGT | *Firmicutes*; *Clostridia*; *Clostridiales* | 74.7 | (WP_016146995) hypothetical protein [*Butyricicoccus* *pullicaecorum*] |  |
| P28_hydA191 | D4ZHLFP1:60:D2HDNACXX:2:1315:12108:32506:GGATGT | *Firmicutes*; *Clostridia*; *Clostridiales* | 69.3 | (WP_021945567) Fe-only hydrogenase catalytic subunit alpha [*Clostridium* sp. CAG:967] |  |
| P28_hydA193 | D4ZHLFP1:60:D2HDNACXX:2:1316:2809:12394:GGATGT | *Firmicutes*; *Clostridia*; *Clostridiales* | 74.7 | (YP_004545925) hydrogenase Fe-only [*Desulfotomaculum* *ruminis* DSM 2154] |  |
| P28_hydA194 | D4ZHLFP1:60:D2HDNACXX:2:1115:12420:59393:GGATGT | *Firmicutes*; *Clostridia*; *Clostridiales* | 90.1 | (WP_008410059) Iron hydrogenase 1 [*Desulfotomaculum* *hydrothermale*] |  |
| P28_hydA196 | D4ZHLFP1:60:D2HDNACXX:2:2308:4906:84336:GGATGT | *Firmicutes*; *Clostridia*; *Clostridiales* | 86.3 | (YP_004517644) hydrogenase, Fe-only [*Desulfotomaculum* *kuznetsovii* DSM 6115] |  |
| P28_hydA2 | D4ZHLFP1:60:D2HDNACXX:2:2115:20070:69253:GGATGT | *Firmicutes*; *Clostridia*; *Clostridiales* | 82.4 | (YP_001212560) hydrogenase subunit [*Pelotomaculum* *thermopropionicum* SI] |  |
| P28_hydA200 | D4ZHLFP1:60:D2HDNACXX:2:2109:13328:77971:GGATGT | *Firmicutes*; *Clostridia*; *Clostridiales* | 50.1 | (YP_001212560) hydrogenase subunit [*Pelotomaculum* *thermopropionicum* SI] |  |
| P28_hydA202 | D4ZHLFP1:60:D2HDNACXX:2:1108:7452:41914:GGATGT | *Firmicutes*; *Clostridia*; *Clostridiales* | 69.7 | (WP_023062885) hydrogenase, Fe-only [*Clostridium* *thermocellum*] |  |
| P28_hydA203 | D4ZHLFP1:60:D2HDNACXX:2:2314:8087:63697:GGATGT | *Firmicutes*; *Clostridia*; *Clostridiales* | 86.7 | (YP_007946226) hydrogenase, Fe-only [*Desulfotomaculum* *gibsoniae* DSM 7213] |  |
| P28_hydA205 | D4ZHLFP1:60:D2HDNACXX:2:1112:11042:33174:GGATGT | *Firmicutes*; *Clostridia*; *Clostridiales* | 79.3 | (YP_004545921) hydrogenase Fe-only [*Desulfotomaculum* *ruminis* DSM 2154] |  |
| P28_hydA209 | D4ZHLFP1:60:D2HDNACXX:2:1107:15147:89949:GGATGT | *Firmicutes*; *Clostridia*; *Clostridiales* | 62.8 | (YP_754500) NADH dehydrogenase I subunit G [*Syntrophomonas* *wolfei* subsp. wolfei str. Goettingen G311] |  |
| P28_hydA211 | D4ZHLFP1:60:D2HDNACXX:2:2115:4317:46474:GGATGT | *Firmicutes*; *Clostridia*; *Clostridiales* | 98.6 | (YP_004517644) hydrogenase, Fe-only [*Desulfotomaculum* *kuznetsovii* DSM 6115] |  |
| P28_hydA214 | D4ZHLFP1:60:D2HDNACXX:2:1112:2518:55363:GGATGT | *Firmicutes*; *Clostridia*; *Clostridiales* | 91.7 | (GAE88236) hydrogenase [*Clostridium* *straminisolvens* JCM 21531] |  |
| P28_hydA221 | D4ZHLFP1:60:D2HDNACXX:2:2115:6996:51529:GGATGT | *Firmicutes*; *Clostridia*; *Clostridiales* | 82.8 | (YP_004496996) hydrogenase, Fe-only [*Desulfotomaculum* *carboxydivorans* CO-1-SRB] |  |
| P28_hydA223 | D4ZHLFP1:60:D2HDNACXX:2:1311:20330:21663:GGATGT | *Firmicutes*; *Clostridia*; *Clostridiales* | 77.8 | (YP_004497002) hydrogenase, Fe-only [*Desulfotomaculum* *carboxydivorans* CO-1-SRB] |  |
| P28_hydA224 | D4ZHLFP1:60:D2HDNACXX:2:2111:19537:17191:GGATGT | *Firmicutes*; *Clostridia*; *Clostridiales* | 73.9 | (WP_004630049) hydrogenase, Fe-only [*Clostridium* *termitidis*] |  |
| P28_hydA225 | D4ZHLFP1:60:D2HDNACXX:2:1308:15996:39808:GGATGT | *Firmicutes*; *Clostridia*; *Clostridiales* | 79.7 | (YP_003189750) hydrogenase, Fe-only [*Desulfotomaculum* *acetoxidans* DSM 771] |  |
| P28_hydA226 | D4ZHLFP1:60:D2HDNACXX:2:1113:13256:62433:GGATGT | *Firmicutes*; *Clostridia*; *Clostridiales* | 95.5 | (WP_006444067) ferredoxin [*Clostridium* *hylemonae*] |  |
| P28_hydA230 | D4ZHLFP1:60:D2HDNACXX:2:2310:11723:51125:GGATGT | *Firmicutes*; *Clostridia*; *Clostridiales* | 68.6 | (YP_003779039) oxidoreductase [*Clostridium* *ljungdahlii* DSM 13528] |  |
| P28_hydA231 | D4ZHLFP1:60:D2HDNACXX:2:2311:17268:5171:GGATGT | *Firmicutes*; *Clostridia*; *Clostridiales* | 58.9 | (YP_004496996) hydrogenase, Fe-only [*Desulfotomaculum* *carboxydivorans* CO-1-SRB] |  |
| P28_hydA238 | D4ZHLFP1:60:D2HDNACXX:2:1112:6714:70609:GGATGT | *Firmicutes*; *Clostridia*; *Clostridiales* | 97.0 | (YP_004517644) hydrogenase, Fe-only [*Desulfotomaculum* *kuznetsovii* DSM 6115] |  |
| P28_hydA24 | D4ZHLFP1:60:D2HDNACXX:2:1304:6732:92947:GGATGT | *Firmicutes*; *Clostridia*; *Clostridiales* | 84.0 | (YP_004497002) hydrogenase, Fe-only [*Desulfotomaculum* *carboxydivorans* CO-1-SRB] |  |
| P28_hydA241 | D4ZHLFP1:60:D2HDNACXX:2:2101:6362:31381:GGATGT | *Firmicutes*; *Clostridia*; *Clostridiales* | 80.1 | (YP_004517644) hydrogenase, Fe-only [*Desulfotomaculum* *kuznetsovii* DSM 6115] |  |
| P28_hydA242 | D4ZHLFP1:60:D2HDNACXX:2:2113:6122:96554:GGATGT | *Firmicutes*; *Clostridia*; *Clostridiales* | 75.5 | (YP_004518295) hydrogenase, Fe-only [*Desulfotomaculum* *kuznetsovii* DSM 6115] |  |
| P28_hydA246 | D4ZHLFP1:60:D2HDNACXX:2:1315:18621:94191:GGATGT | *Firmicutes*; *Clostridia*; *Clostridiales* | 84.3 | (YP_007946226) hydrogenase, Fe-only [*Desulfotomaculum* *gibsoniae* DSM 7213] |  |
| P28_hydA247 | D4ZHLFP1:60:D2HDNACXX:2:1108:2356:26602:GGATGT | *Firmicutes*; *Clostridia*; *Clostridiales* | 82.4 | (WP_021908252) hydrogenase Fe-only [*Eubacterium* sp. CAG:146] |  |
| P28_hydA248 | D4ZHLFP1:60:D2HDNACXX:2:1105:4287:47028:GGATGT | *Firmicutes*; *Clostridia*; *Clostridiales* | 72.4 | (YP_001212560) hydrogenase subunit [*Pelotomaculum* *thermopropionicum* SI] |  |
| P28_hydA249 | D4ZHLFP1:60:D2HDNACXX:2:2109:14906:83643:GGATGT | *Firmicutes*; *Clostridia*; *Clostridiales* | 59.7 | (WP_021675481) putative ferredoxin hydrogenase [*Peptostreptococcaceae* *bacterium* oral taxon 113] |  |
| P28_hydA250 | D4ZHLFP1:60:D2HDNACXX:2:1302:11377:92286:GGATGT | *Firmicutes*; *Clostridia*; *Clostridiales* | 93.0 | (YP_004545921) hydrogenase Fe-only [*Desulfotomaculum* *ruminis* DSM 2154] |  |
| P28_hydA253 | D4ZHLFP1:60:D2HDNACXX:2:1102:2651:71684:GGATGT | *Firmicutes*; *Clostridia*; *Clostridiales* | 81.6 | (YP_007947570) hydrogenase, Fe-only [*Desulfotomaculum* *gibsoniae* DSM 7213] |  |
| P28_hydA289 | D4ZHLFP1:60:D2HDNACXX:2:1116:10190:42803:GGATGT | *Firmicutes*; *Clostridia*; *Clostridiales* | 47.0 | (YP_001113004) hydrogenase [*Desulfotomaculum* *reducens* MI-1] |  |
| P28_hydA102 | D4ZHLFP1:60:D2HDNACXX:2:2115:5878:100510:GGATGT | *Firmicutes*; *Clostridia*; *Clostridiales* | 83.2 | (YP_004498193) hydrogenase, Fe-only [*Desulfotomaculum* *carboxydivorans* CO-1-SRB] |  |
| P28_hydA110 | D4ZHLFP1:60:D2HDNACXX:2:2109:4033:21864:GGATGT | *Firmicutes*; *Clostridia*; *Clostridiales* | 72.8 | (WP_008410059) Iron hydrogenase 1 [*Desulfotomaculum* *hydrothermale*] |  |
| P28_hydA13 | D4ZHLFP1:60:D2HDNACXX:2:1301:7743:36721:GGATGT | *Firmicutes*; *Clostridia*; *Clostridiales* | 67.0 | (GAE88236) hydrogenase [*Clostridium* *straminisolvens* JCM 21531] |  |
| P28_hydA240 | D4ZHLFP1:60:D2HDNACXX:2:1102:10549:18234:GGATGT | *Firmicutes*; *Clostridia*; *Halanaerobiales* | 84.7 | (YP_003828850) NAD(P)-dependent iron-only hydrogenase [*Acetohalobium arabaticum* DSM 5501] |  |
| P28_hydA262 | D4ZHLFP1:60:D2HDNACXX:2:1311:7479:6204:GGATGT | *Firmicutes*; *Clostridia*; *Halanaerobiales* | 56.6 | (YP_003826883) NAD(P)-dependent iron-only hydrogenase [*Acetohalobium arabaticum* DSM 5501] |  |
| P28_hydA280 | D4ZHLFP1:60:D2HDNACXX:2:2315:18278:80300:GGATGT | *Firmicutes*; *Clostridia*; *Halanaerobiales* | 91.7 | (YP_003828451) NAD(P)-dependent iron-only hydrogenase catalytic subunit [*Acetohalobium arabaticum* DSM 5501] |  |
| P28_hydA31 | D4ZHLFP1:60:D2HDNACXX:2:2305:8863:73684:GGATGT | *Firmicutes*; *Clostridia*; *Halanaerobiales* | 75.1 | (YP_007316252) hydrogenase, Fe-only [*Halobacteroides* *halobius* DSM 5150] |  |
| P28_hydA313 | D4ZHLFP1:60:D2HDNACXX:2:1107:5724:58972:GGATGT | *Firmicutes*; *Clostridia*; *Halanaerobiales* | 87.8 | (YP_005836915) NAD(P)-dependent iron-only hydrogenase iron-sulfur protein [*Halanaerobium* *praevalens* DSM 2228] |  |
| P28_hydA328 | D4ZHLFP1:60:D2HDNACXX:2:1103:3193:8355:GGATGT | *Firmicutes*; *Clostridia*; *Halanaerobiales* | 86.3 | (YP_003828850) NAD(P)-dependent iron-only hydrogenase [*Acetohalobium arabaticum* DSM 5501] |  |
| P28_hydA336 | D4ZHLFP1:60:D2HDNACXX:2:1110:16335:17264:GGATGT | *Firmicutes*; *Clostridia*; *Halanaerobiales* | 54.7 | (YP_005836913) NAD(P)-dependent iron-only hydrogenase catalytic subunit [*Halanaerobium* *praevalens* DSM 2228] |  |
| P28_hydA353 | D4ZHLFP1:60:D2HDNACXX:2:1112:11959:67712:GGATGT | *Firmicutes*; *Clostridia*; *Halanaerobiales* | 53.5 | (YP_007316252) hydrogenase, Fe-only [*Halobacteroides* *halobius* DSM 5150] |  |
| P28_hydA372 | D4ZHLFP1:60:D2HDNACXX:2:2107:9997:60658:GGATGT | *Firmicutes*; *Clostridia*; *Halanaerobiales* | 77.4 | (YP_003828850) NAD(P)-dependent iron-only hydrogenase [*Acetohalobium arabaticum* DSM 5501] |  |
| P28_hydA419 | D4ZHLFP1:60:D2HDNACXX:2:1313:15899:71497:GGATGT | *Firmicutes*; *Clostridia*; *Halanaerobiales* | 68.9 | (YP_005836915) NAD(P)-dependent iron-only hydrogenase iron-sulfur protein [*Halanaerobium* *praevalens* DSM 2228] |  |
| P28_hydA425 | D4ZHLFP1:60:D2HDNACXX:2:2103:7854:41623:GGATGT | *Firmicutes*; *Clostridia*; *Halanaerobiales* | 61.2 | (YP_005836915) NAD(P)-dependent iron-only hydrogenase iron-sulfur protein [*Halanaerobium* *praevalens* DSM 2228] |  |
| P28_hydA55 | D4ZHLFP1:60:D2HDNACXX:2:2307:15234:42821:GGATGT | *Firmicutes*; *Clostridia*; *Halanaerobiales* | 69.7 | (YP_003826885) NAD(P)-dependent iron-only hydrogenase catalytic subunit [*Acetohalobium arabaticum* DSM 5501] |  |
| P28_hydA80 | D4ZHLFP1:60:D2HDNACXX:2:2305:7709:26197:GGATGT | *Firmicutes*; *Clostridia*; *Halanaerobiales* | 78.6 | (YP_003828850) NAD(P)-dependent iron-only hydrogenase [*Acetohalobium arabaticum* DSM 5501] |  |
| P28_hydA143 | D4ZHLFP1:60:D2HDNACXX:2:1312:17755:31435:GGATGT | *Firmicutes*; *Clostridia*; *Halanaerobiales* | 73.9 | (YP_003826883) NAD(P)-dependent iron-only hydrogenase [*Acetohalobium arabaticum* DSM 5501] |  |
| P28_hydA151 | D4ZHLFP1:60:D2HDNACXX:2:1306:8606:79764:GGATGT | *Firmicutes*; *Clostridia*; *Halanaerobiales* | 65.5 | (YP_005836913) NAD(P)-dependent iron-only hydrogenase catalytic subunit [*Halanaerobium* *praevalens* DSM 2228] |  |
| P28_hydA115 | D4ZHLFP1:60:D2HDNACXX:2:2306:2069:49841:GGATGT | *Firmicutes*; *Clostridia*; *Halanaerobiales* | 63.5 | (WP_018249208) NADH dehydrogenase [*Orenia* *marismortui*] |  |
| P28_hydA120 | D4ZHLFP1:60:D2HDNACXX:2:2111:6873:11974:GGATGT | *Firmicutes*; *Clostridia*; *Halanaerobiales* | 57.0 | (YP_003826883) NAD(P)-dependent iron-only hydrogenase [*Acetohalobium arabaticum* DSM 5501] |  |
| P28_hydA158 | D4ZHLFP1:60:D2HDNACXX:2:1107:14111:95140:GGATGT | *Firmicutes*; *Clostridia*; *Halanaerobiales* | 53.9 | (YP_003826885) NAD(P)-dependent iron-only hydrogenase catalytic subunit [*Acetohalobium arabaticum* DSM 5501] |  |
| P28_hydA177 | D4ZHLFP1:60:D2HDNACXX:2:1114:17220:95674:GGATGT | *Firmicutes*; *Clostridia*; *Halanaerobiales* | 80.1 | (YP_003828850) NAD(P)-dependent iron-only hydrogenase [*Acetohalobium arabaticum* DSM 5501] |  |
| P28_hydA218 | D4ZHLFP1:60:D2HDNACXX:2:2116:15353:54799:GGATGT | *Firmicutes*; *Clostridia*; *Thermoanaerobacterales* | 81.6 | (YP_430562) Iron hydrogenase, small subunit [*Moorella thermoacetica* ATCC 39073] |  |
| P28_hydA259 | D4ZHLFP1:60:D2HDNACXX:2:1304:15254:15255:GGATGT | *Firmicutes*; *Clostridia*; *Thermoanaerobacterales* | 81.3 | (WP_004401666) hydrogenase, Fe-only [*Thermoanaerobacter* *thermohydrosulfuricus*] |  |
| P28_hydA284 | D4ZHLFP1:60:D2HDNACXX:2:1302:4272:3726:GGATGT | *Firmicutes*; *Clostridia*; *Thermoanaerobacterales* | 84.0 | (YP_004461911) hydrogenase, Fe-only [*Tepidanaerobacter* *acetatoxydans* Re1] |  |
| P28_hydA285 | D4ZHLFP1:60:D2HDNACXX:2:1116:10437:74421:GGATGT | *Firmicutes*; *Clostridia*; *Thermoanaerobacterales* | 73.6 | (GAF24846) Fe-only hydrogenase large subunit, C-terminal domain [*Moorella thermoacetica* Y72] |  |
| P28_hydA286 | D4ZHLFP1:60:D2HDNACXX:2:2301:14265:87761:GGATGT | *Firmicutes*; *Clostridia*; *Thermoanaerobacterales* | 67.4 | (YP_004461324) hydrogenase, Fe-only [*Tepidanaerobacter* *acetatoxydans* Re1] |  |
| P28_hydA288 | D4ZHLFP1:60:D2HDNACXX:2:1108:9442:99025:GGATGT | *Firmicutes*; *Clostridia*; *Thermoanaerobacterales* | 59.3 | (YP_003239720) hydrogenase, Fe-only [*Ammonifex degensii* KC4] |  |
| P28_hydA300 | D4ZHLFP1:60:D2HDNACXX:2:2307:5041:20659:GGATGT | *Firmicutes*; *Clostridia*; *Thermoanaerobacterales* | 68.9 | (GAF24846) Fe-only hydrogenase large subunit, C-terminal domain [*Moorella thermoacetica* Y72] |  |
| P28_hydA308 | D4ZHLFP1:60:D2HDNACXX:2:2304:10101:67081:GGATGT | *Firmicutes*; *Clostridia*; *Thermoanaerobacterales* | 79.0 | (YP_002246551) hydrogenase [*Coprothermobacter* *proteolyticus* DSM 5265] |  |
| P28_hydA32 | D4ZHLFP1:60:D2HDNACXX:2:1316:17659:45702:GGATGT | *Firmicutes*; *Clostridia*; *Thermoanaerobacterales* | 82.0 | (GAF24846) Fe-only hydrogenase large subunit, C-terminal domain [*Moorella thermoacetica* Y72] |  |
| P28_hydA327 | D4ZHLFP1:60:D2HDNACXX:2:1308:16700:99537:GGATGT | *Firmicutes*; *Clostridia*; *Thermoanaerobacterales* | 73.2 | (YP_003825085) NAD(P)-dependent iron-only hydrogenase [*Thermosediminibacter* *oceani* DSM 16646] |  |
| P28_hydA383 | D4ZHLFP1:60:D2HDNACXX:2:2315:12231:55852:GGATGT | *Firmicutes*; *Clostridia*; *Thermoanaerobacterales* | 73.9 | (YP_001180640) hydrogenase, Fe-only [*Caldicellulosiruptor* *saccharolyticus* DSM 8903] |  |
| P28_hydA39 | D4ZHLFP1:60:D2HDNACXX:2:2103:7070:24853:GGATGT | *Firmicutes*; *Clostridia*; *Thermoanaerobacterales* | 69.3 | (YP_001180640) hydrogenase, Fe-only [*Caldicellulosiruptor* *saccharolyticus* DSM 8903] |  |
| P28_hydA393 | D4ZHLFP1:60:D2HDNACXX:2:1305:9485:70857:GGATGT | *Firmicutes*; *Clostridia*; *Thermoanaerobacterales* | 70.1 | (YP_004461326) NAD(P)-dependent iron-only hydrogenase iron-sulfur protein [*Tepidanaerobacter* acetatoxydans Re1] |  |
| P28_hydA411 | D4ZHLFP1:60:D2HDNACXX:2:2111:8329:58755:GGATGT | *Firmicutes*; *Clostridia*; *Thermoanaerobacterales* | 72.4 | (YP_002246551) hydrogenase [*Coprothermobacter* *proteolyticus* DSM 5265] |  |
| P28_hydA434 | D4ZHLFP1:60:D2HDNACXX:2:2303:15736:81598:GGATGT | *Firmicutes*; *Clostridia*; *Thermoanaerobacterales* | 72.4 | (YP_002246551) hydrogenase [*Coprothermobacter* *proteolyticus* DSM 5265] |  |
| P28_hydA451 | D4ZHLFP1:60:D2HDNACXX:2:2314:3668:64579:GGATGT | *Firmicutes*; *Clostridia*; *Thermoanaerobacterales* | 88.2 | (YP_007298421) hydrogenase, Fe-only [*Thermoanaerobacterium thermosaccharolyticum* M0795] |  |
| P28_hydA466 | D4ZHLFP1:60:D2HDNACXX:2:1308:11913:50654:GGATGT | *Firmicutes*; *Clostridia*; *Thermoanaerobacterales* | 85.5 | (YP_004463270) NAD(P)-dependent iron-only hydrogenase catalytic subunit [*Mahella* *australiensis* 50-1 BON] |  |
| P28_hydA469 | D4ZHLFP1:60:D2HDNACXX:2:1303:16259:55923:GGATGT | *Firmicutes*; *Clostridia*; *Thermoanaerobacterales* | 69.7 | (GAF24846) Fe-only hydrogenase large subunit [*Moorella thermoacetica* Y72] |  |
| P28_hydA470 | D4ZHLFP1:60:D2HDNACXX:2:2106:4343:70694:GGATGT | *Firmicutes*; *Clostridia*; *Thermoanaerobacterales* | 75.9 | (GAF24846) Fe-only hydrogenase large subunit [*Moorella thermoacetica* Y72] |  |
| P28_hydA60 | D4ZHLFP1:60:D2HDNACXX:2:2115:7729:59639:GGATGT | *Firmicutes*; *Clostridia*; *Thermoanaerobacterales* | 94.0 | (YP_004438253) hydrogenase, Fe-only [*Thermodesulfobium* *narugense* DSM 14796] |  |
| P28_hydA98 | D4ZHLFP1:60:D2HDNACXX:2:2305:19517:28054:GGATGT | *Firmicutes*; *Clostridia*; *Thermoanaerobacterales* | 59.3 | (YP_001180640) hydrogenase, Fe-only [*Caldicellulosiruptor* *saccharolyticus* DSM 8903] |  |
| P28_hydA184 | D4ZHLFP1:60:D2HDNACXX:2:1306:7062:27269:GGATGT | *Firmicutes*; *Clostridia*; *Thermoanaerobacterales* | 77.8 | (YP_003239720) hydrogenase, Fe-only [*Ammonifex degensii* KC4] |  |
| P28_hydA164 | D4ZHLFP1:60:D2HDNACXX:2:2301:9841:57513:GGATGT | *Firmicutes*; *Clostridia*; *Thermoanaerobacterales* | 79.3 | (YP_002246551) hydrogenase [*Coprothermobacter* *proteolyticus* DSM 5265] |  |
| P28_hydA21 | D4ZHLFP1:60:D2HDNACXX:2:2108:11337:43438:GGATGT | *Firmicutes*; *Clostridia*; *Thermoanaerobacterales* | 95.5 | (YP_007298421) hydrogenase, Fe-only [*Thermoanaerobacterium thermosaccharolyticum* M0795] |  |
| P28_hydA212 | D4ZHLFP1:60:D2HDNACXX:2:2109:7268:98405:GGATGT | *Firmicutes*; *Negativicutes*; *Selenomonadales* | 81.3 | (WP_021167212) NADP-reducing hydrogenase subunit HndC [*Sporomusa ovata*] |  |
| P28_hydA291 | D4ZHLFP1:60:D2HDNACXX:2:1314:7803:60983:GGATGT | *Firmicutes*; *Negativicutes*; *Selenomonadales* | 50.1 | (WP_021167102) NADP-reducing hydrogenase subunit HndC [*Sporomusa ovata*] |  |
| P28_hydA0 | D4ZHLFP1:60:D2HDNACXX:2:1107:8897:81612:GGATGT | *Firmicutes*; *Negativicutes*; *Selenomonadales* | 40.0 | (ETS92418) [FeFe] hydrogenase, group A [*Veillonella* sp. AS16] |  |
| P28_hydA103 | D4ZHLFP1:60:D2HDNACXX:2:1108:18262:37422:GGATGT | *Firmicutes*; *Negativicutes*; *Selenomonadales* | 56.6 | (WP_021167212) NADP-reducing hydrogenase subunit HndC [*Sporomusa ovata*] | |
| P28_hydA106 | D4ZHLFP1:60:D2HDNACXX:2:2111:6537:97386:GGATGT | *Firmicutes*; *Negativicutes*; *Selenomonadales* | 42.0 | (ETS92418) [FeFe] hydrogenase, group A [*Veillonella* sp. AS16] | |
| P28_hydA109 | D4ZHLFP1:60:D2HDNACXX:2:1101:12380:24778:GGATGT | *Firmicutes*; *Negativicutes*; *Selenomonadales* | 70.5 | (WP_021167102) NADP-reducing hydrogenase subunit HndC [*Sporomusa ovata*] |  |
| P28_hydA403 | D4ZHLFP1:60:D2HDNACXX:2:2313:4078:52817:GGATGT | *Fusobacteria*; *Fusobacteriales* | 84.0 | (YP_003966824) NAD(P)-dependent iron-only hydrogenase catalytic subunit [*Ilyobacter polytropus* DSM 2926] | |
| P28_hydA180 | D4ZHLFP1:60:D2HDNACXX:2:2309:6802:75822:GGATGT | *Ignavibacteria*; *Ignavibacteriales* | 87.4 | (YP_005845237) Iron-only hydrogenase large subunit [*Ignavibacterium album* JCM 16511] |  |
| P28_hydA243 | D4ZHLFP1:60:D2HDNACXX:2:2314:9574:61300:GGATGT | *Ignavibacteria*; *Ignavibacteriales* | 87.0 | (YP_006526890) Fe-only hydrogenase, catalytic subunit alpha [*Melioribacter* *roseus* P3M-2] | |
| P28_hydA33 | D4ZHLFP1:60:D2HDNACXX:2:1310:8731:83856:GGATGT | *Ignavibacteria*; *Ignavibacteriales* | 75.9 | (YP_005845237) Fe-only hydrogenase large subunit [*Ignavibacterium* *album* JCM 16511] |  |
| P28_hydA118 | D4ZHLFP1:60:D2HDNACXX:2:2111:21061:77159:GGATGT | *Deltaproteobacteria*; *Desulfobacterales* | 88.6 | (YP_004195044) NAD(P)-dependent iron-only hydrogenase catalytic subunit [*Desulfobulbus propionicus* DSM 2032] |  |
| P28_hydA138 | D4ZHLFP1:60:D2HDNACXX:2:1311:13431:91130:GGATGT | *Deltaproteobacteria*; *Desulfobacterales* | 82.8 | (YP_002952160) Fe hydrogenase [*Desulfovibrio magneticus* RS-1] |  |
| P28_hydA142 | D4ZHLFP1:60:D2HDNACXX:2:2107:9900:26341:GGATGT | *Deltaproteobacteria*; *Desulfobacterales* | 77.8 | (WP_023407578) hypothetical protein [uncultured *Desulfofustis* sp. PB-SRB1] |  |
| P28_hydA206 | D4ZHLFP1:60:D2HDNACXX:2:1310:16127:97033:GGATGT | *Deltaproteobacteria*; *Desulfobacterales* | 63.9 | (YP_004195044) NAD(P)-dependent iron-only hydrogenase catalytic subunit [*Desulfobulbus propionicus* DSM 2032] |  |
| P28_hydA23 | D4ZHLFP1:60:D2HDNACXX:2:2311:4184:36432:GGATGT | *Deltaproteobacteria*; *Desulfobacterales* | 86.3 | (YP_004195044) NAD(P)-dependent iron-only hydrogenase catalytic subunit [*Desulfobulbus propionicus* DSM 2032] |  |
| P28_hydA25 | D4ZHLFP1:60:D2HDNACXX:2:2102:9683:12556:GGATGT | *Deltaproteobacteria*; *Desulfobacterales* | 79.7 | (YP_004195044) NAD(P)-dependent iron-only hydrogenase catalytic subunit [*Desulfobulbus propionicus* DSM 2032] |  |
| P28_hydA258 | D4ZHLFP1:60:D2HDNACXX:2:2108:11702:74298:GGATGT | *Deltaproteobacteria*; *Desulfobacterales* | 63.9 | (YP_004195044) NAD(P)-dependent iron-only hydrogenase catalytic subunit [*Desulfobulbus propionicus* DSM 2032] |  |
| P28_hydA421 | D4ZHLFP1:60:D2HDNACXX:2:2316:12545:22203:GGATGT | *Deltaproteobacteria*; *Desulfobacterales* | 69.7 | (YP_004195044) NAD(P)-dependent iron-only hydrogenase catalytic subunit [*Desulfobulbus propionicus* DSM 2032] |  |
| P28_hydA436 | D4ZHLFP1:60:D2HDNACXX:2:1105:10075:54351:GGATGT | *Deltaproteobacteria*; *Desulfobacterales* | 61.6 | (YP_004195044) NAD(P)-dependent iron-only hydrogenase catalytic subunit [*Desulfobulbus propionicus* DSM 2032] |  |
| P28_hydA440 | D4ZHLFP1:60:D2HDNACXX:2:1308:9791:16364:GGATGT | *Deltaproteobacteria*; *Desulfobacterales* | 87.4 | (YP_004195044) NAD(P)-dependent iron-only hydrogenase catalytic subunit [*Desulfobulbus propionicus* DSM 2032] |  |
| P28_hydA448 | D4ZHLFP1:60:D2HDNACXX:2:2308:11112:4719:GGATGT | *Deltaproteobacteria*; *Desulfobacterales* | 52.0 | (YP_004195044) NAD(P)-dependent iron-only hydrogenase catalytic subunit [*Desulfobulbus propionicus* DSM 2032] |  |
| P28_hydA58 | D4ZHLFP1:60:D2HDNACXX:2:2308:11112:4719:GGATGT | *Deltaproteobacteria*; *Desulfobacterales* | 66.2 | (YP_004195044) NAD(P)-dependent iron-only hydrogenase catalytic subunit [*Desulfobulbus propionicus* DSM 2032] |  |
| P28_hydA354 | D4ZHLFP1:60:D2HDNACXX:2:1308:15477:10829:GGATGT | *Deltaproteobacteria*; *Desulfovibrionales* | 90.1 | (WP_006921111) hydrogenase, Fe-only, partial [*Desulfovibrio magneticus*] |  |
| P28_hydA389 | D4ZHLFP1:60:D2HDNACXX:2:2315:17485:12079:GGATGT | *Deltaproteobacteria*; *Desulfovibrionales* | 85.9 | (YP_002952160) Fe hydrogenase [*Desulfovibrio magneticus* RS-1] |  |
| P28_hydA391 | D4ZHLFP1:60:D2HDNACXX:2:2115:13942:46150:GGATGT | *Deltaproteobacteria*; *Desulfovibrionales* | 83.6 | (YP_004195044) NAD(P)-dependent iron-only hydrogenase catalytic subunit [*Desulfobulbus propionicus* DSM 2032] |  |
| P28_hydA44 | D4ZHLFP1:60:D2HDNACXX:2:1105:16776:32194:GGATGT | *Deltaproteobacteria*; *Desulfovibrionales* | 77.4 | (WP_006921111) hydrogenase, Fe-only, partial [*Desulfovibrio magneticus*] |  |
| P28_hydA446 | D4ZHLFP1:60:D2HDNACXX:2:1316:8749:40017:GGATGT | *Deltaproteobacteria*; *Desulfovibrionales* | 88.2 | (WP_006921111) hydrogenase, Fe-only, partial [*Desulfovibrio magneticus*] |  |
| P28_hydA64 | D4ZHLFP1:60:D2HDNACXX:2:2302:1649:3807:GGATGT | *Deltaproteobacteria*; *Desulfovibrionales* | 95.5 | (WP_006921111) hydrogenase, Fe-only, partial [*Desulfovibrio magneticus*] |  |
| P28_hydA83 | D4ZHLFP1:60:D2HDNACXX:2:1312:9567:93217:GGATGT | *Deltaproteobacteria*; *Desulfovibrionales* | 55.1 | (WP_006921111) hydrogenase, Fe-only, partial [*Desulfovibrio magneticus*] |  |
| P28_hydA295 | D4ZHLFP1:60:D2HDNACXX:2:1312:7905:70603:GGATGT | *Deltaproteobacteria*; *Desulfuromonadales* | 62.0 | (YP_901164) hydrogenase, Fe-only [*Pelobacter* *propionicus* DSM 2379] |  |
| P28_hydA256 | D4ZHLFP1:60:D2HDNACXX:2:1101:7156:4545:GGATGT | *Deltaproteobacteria*; *Syntrophobacterales* | 93.2 | (YP_844976) hydrogenase, Fe-only [*Syntrophobacter fumaroxidans* MPOB] |  |
| P28_hydA401 | D4ZHLFP1:60:D2HDNACXX:2:1111:5768:93829:GGATGT | *Deltaproteobacteria*; *Syntrophobacterales* | 95.0 | (YP_844976) hydrogenase, Fe-only [*Syntrophobacter fumaroxidans* MPOB] |  |
| P28_hydA441 | D4ZHLFP1:60:D2HDNACXX:2:1102:8008:40859:GGATGT | *Deltaproteobacteria*; *Syntrophobacterales* | 84.3 | (YP_844976) hydrogenase, Fe-only [*Syntrophobacter fumaroxidans* MPOB] |  |
| P28_hydA416 | D4ZHLFP1:60:D2HDNACXX:2:2111:21254:19461:GGATGT | *Deltaproteobacteria*; *Desulfobacterales* | 50.1 | (YP_004195044) NAD(P)-dependent iron-only hydrogenase catalytic subunit [*Desulfobulbus propionicus* DSM 2032] |  |
| P28_hydA455 | D4ZHLFP1:60:D2HDNACXX:2:2310:9367:11409:GGATGT | *Deltaproteobacteria*; *Syntrophobacterales* | 62.4 | (YP_462897) 2Fe-2S and 4Fe-4S iron-sulfur protein [*Syntrophus* *aciditrophicus* SB] |  |
| P28_hydA458 | D4ZHLFP1:60:D2HDNACXX:2:1107:6062:45666:GGATGT | *Deltaproteobacteria*; *Syntrophobacterales* | 71.6 | (YP_462897) 2Fe-2S and 4Fe-4S iron-sulfur protein [*Syntrophus* *aciditrophicus* SB] |  |
| P28_hydA57 | D4ZHLFP1:60:D2HDNACXX:2:1307:18676:50166:GGATGT | *Deltaproteobacteria*; *Syntrophobacterales* | 78.6 | (YP_844976) hydrogenase, Fe-only [*Syntrophobacter fumaroxidans* MPOB] |  |
| P28_hydA67 | D4ZHLFP1:60:D2HDNACXX:2:1108:2150:17747:GGATGT | *Deltaproteobacteria*; *Syntrophobacterales* | 87.8 | (YP_844976) hydrogenase, Fe-only [*Syntrophobacter fumaroxidans* MPOB] |  |
| P28_hydA101 | D4ZHLFP1:60:D2HDNACXX:2:1108:15132:54838:GGATGT | *Deltaproteobacteria*; *Desulfovibrionales* | 86.3 | (WP_006921111) hydrogenase, Fe-only, partial [*Desulfovibrio magneticus*] |  |
| P28_hydA140 | D4ZHLFP1:60:D2HDNACXX:2:2311:12290:15501:GGATGT | *Deltaproteobacteria*; *Desulfobacterales* | 50.1 | (YP_004195044) NAD(P)-dependent iron-only hydrogenase catalytic subunit [*Desulfobulbus propionicus* DSM 2032] |  |
| P28_hydA149 | D4ZHLFP1:60:D2HDNACXX:2:2109:3943:11784:GGATGT | *Deltaproteobacteria*; *Desulfobacterales* | 88.6 | (YP_004195044) NAD(P)-dependent iron-only hydrogenase catalytic subunit [*Desulfobulbus propionicus* DSM 2032] |  |
| P28_hydA15 | D4ZHLFP1:60:D2HDNACXX:2:2114:13414:98639:GGATGT | *Deltaproteobacteria*; *Desulfuromonadales* | 83.6 | (YP_901164) hydrogenase, Fe-only [*Pelobacter* *propionicus* DSM 2379] |  |
| P28_hydA16 | D4ZHLFP1:60:D2HDNACXX:2:2116:6328:14789:GGATGT | *Deltaproteobacteria*; *Desulfobacterales* | 63.9 | (YP_004195044) NAD(P)-dependent iron-only hydrogenase catalytic subunit [*Desulfobulbus propionicus* DSM 2032] |  |
| P28_hydA197 | D4ZHLFP1:60:D2HDNACXX:2:2101:14114:76366:GGATGT | *Spirochaetes*; *Spirochaetales* | 93.6 | (YP_003802215) NAD(P)-dependent iron-only hydrogenase catalytic subunit [*Spirochaeta smaragdinae* DSM 11293] |  |
| P28_hydA210 | D4ZHLFP1:60:D2HDNACXX:2:2302:1998:88206:GGATGT | *Spirochaetes*; *Spirochaetales* | 79.7 | (YP_003802105) hydrogenase, Fe-only [*Spirochaeta smaragdinae* DSM 11293] |  |
| P28_hydA217 | D4ZHLFP1:60:D2HDNACXX:2:2303:12031:86571:GGATGT | *Spirochaetes*; *Spirochaetales* | 88.2 | (YP_003802105) hydrogenase, Fe-only [*Spirochaeta smaragdinae* DSM 11293] |  |
| P28_hydA227 | D4ZHLFP1:60:D2HDNACXX:2:1311:8720:79761:GGATGT | *Spirochaetes*; *Spirochaetales* | 72.0 | (YP_005475514) hydrogenase, Fe-only [*Spirochaeta africana* DSM 8902] |  |
| P28_hydA229 | D4ZHLFP1:60:D2HDNACXX:2:1110:6655:64887:GGATGT | *Spirochaetes*; *Spirochaetales* | 80.9 | (YP_005475514) hydrogenase, Fe-only [*Spirochaeta africana* DSM 8902] |  |
| P28_hydA237 | D4ZHLFP1:60:D2HDNACXX:2:2107:8025:67535:GGATGT | *Spirochaetes*; *Spirochaetales* | 97.4 | (YP_003802215) NAD(P)-dependent iron-only hydrogenase catalytic subunit [*Spirochaeta smaragdinae* DSM 11293] |  |
| P28_hydA251 | D4ZHLFP1:60:D2HDNACXX:2:2115:16128:90091:GGATGT | *Spirochaetes*; *Spirochaetales* | 83.2 | (YP_003802215) NAD(P)-dependent iron-only hydrogenase catalytic subunit [*Spirochaeta smaragdinae* DSM 11293] |  |
| P28_hydA252 | D4ZHLFP1:60:D2HDNACXX:2:2309:8988:94473:GGATGT | *Spirochaetes*; *Spirochaetales* | 76.3 | (YP_005475514) hydrogenase, Fe-only [*Spirochaeta africana* DSM 8902] |  |
| P28_hydA254 | D4ZHLFP1:60:D2HDNACXX:2:1115:16928:10462:GGATGT | *Spirochaetes*; *Spirochaetales* | 70.5 | (YP_005475514) hydrogenase, Fe-only [*Spirochaeta africana* DSM 8902] |  |
| P28_hydA279 | D4ZHLFP1:60:D2HDNACXX:2:2109:15795:73802:GGATGT | *Spirochaetes*; *Spirochaetales* | 79.3 | (YP_003802215) NAD(P)-dependent iron-only hydrogenase catalytic subunit [*Spirochaeta smaragdinae* DSM 11293] |  |
| P28_hydA290 | D4ZHLFP1:60:D2HDNACXX:2:2307:14352:43488:GGATGT | *Spirochaetes*; *Spirochaetales* | 75.9 | (YP_003802215) NAD(P)-dependent iron-only hydrogenase catalytic subunit [*Spirochaeta smaragdinae* DSM 11293] |  |
| P28_hydA317 | D4ZHLFP1:60:D2HDNACXX:2:2101:9681:95732:GGATGT | *Spirochaetes*; *Spirochaetales* | 86.7 | (YP_003802105) hydrogenase, Fe-only [*Spirochaeta smaragdinae* DSM 11293] |  |
| P28_hydA351 | D4ZHLFP1:60:D2HDNACXX:2:1310:12256:38899:GGATGT | *Spirochaetes*; *Spirochaetales* | 80.1 | (YP_005475514) hydrogenase, Fe-only [*Spirochaeta africana* DSM 8902] |  |
| P28_hydA386 | D4ZHLFP1:60:D2HDNACXX:2:1108:9206:11716:GGATGT | *Spirochaetes*; *Spirochaetales* | 64.7 | (YP_005061073) hydrogenase, Fe-only [*Sphaerochaeta pleomorpha* str. Grapes] |  |
| P28_hydA387 | D4ZHLFP1:60:D2HDNACXX:2:2103:8711:61592:GGATGT | *Spirochaetes*; *Spirochaetales* | 77.8 | (YP_003802215) NAD(P)-dependent iron-only hydrogenase catalytic subunit [*Spirochaeta smaragdinae* DSM 11293] |  |
| P28_hydA398 | D4ZHLFP1:60:D2HDNACXX:2:1105:3172:49214:GGATGT | *Spirochaetes*; *Spirochaetales* | 87.8 | (YP_003802105) hydrogenase, Fe-only [*Spirochaeta smaragdinae* DSM 11293] |  |
| P28_hydA418 | D4ZHLFP1:60:D2HDNACXX:2:1109:3864:42746:GGATGT | *Spirochaetes*; *Spirochaetales* | 85.1 | (YP_003802215) NAD(P)-dependent iron-only hydrogenase catalytic subunit [*Spirochaeta smaragdinae* DSM 11293] |  |
| P28_hydA422 | D4ZHLFP1:60:D2HDNACXX:2:2114:20411:45230:GGATGT | *Spirochaetes*; *Spirochaetales* | 90.1 | (YP_003802215) NAD(P)-dependent iron-only hydrogenase catalytic subunit [*Spirochaeta smaragdinae* DSM 11293] |  |
| P28_hydA444 | D4ZHLFP1:60:D2HDNACXX:2:1108:15662:57230:GGATGT | *Spirochaetes*; *Spirochaetales* | 71.2 | (YP_005475514) hydrogenase, Fe-only [*Spirochaeta africana* DSM 8902] |  |
| P28_hydA447 | D4ZHLFP1:60:D2HDNACXX:2:1314:12915:31002:GGATGT | *Spirochaetes*; *Spirochaetales* | 91.0 | (YP_005475514) hydrogenase, Fe-only [*Spirochaeta africana* DSM 8902] |  |
| P28_hydA456 | D4ZHLFP1:60:D2HDNACXX:2:1107:9964:40383:GGATGT | *Spirochaetes*; *Spirochaetales* | 60.8 | (YP_006044637) hydrogenase, Fe-only [*Spirochaeta* *thermophila* DSM 6578] |  |
| P28_hydA460 | D4ZHLFP1:60:D2HDNACXX:2:1302:5716:41810:GGATGT | *Spirochaetes*; *Spirochaetales* | 81.3 | (YP_003802215) NAD(P)-dependent iron-only hydrogenase catalytic subunit [*Spirochaeta smaragdinae* DSM 11293] |  |
| P28_hydA62 | D4ZHLFP1:60:D2HDNACXX:2:2310:16315:59036:GGATGT | *Spirochaetes*; *Spirochaetales* | 82.8 | (YP_003802215) NAD(P)-dependent iron-only hydrogenase catalytic subunit [*Spirochaeta smaragdinae* DSM 11293] |  |
| P28_hydA266 | D4ZHLFP1:60:D2HDNACXX:2:1108:15952:64033:GGATGT | *Spirochaetes*; *Spirochaetales* | 95.1 | (YP_003802215) NAD(P)-dependent iron-only hydrogenase catalytic subunit [*Spirochaeta smaragdinae* DSM 11293] |  |
| P28_hydA10 | D4ZHLFP1:60:D2HDNACXX:2:1308:5318:45616:GGATGT | *Spirochaetes*; *Spirochaetales* | 71.2 | (YP_003802105) hydrogenase, Fe-only [*Spirochaeta smaragdinae* DSM 11293] |  |
| P28_hydA108 | D4ZHLFP1:60:D2HDNACXX:2:1303:8337:4917:GGATGT | *Spirochaetes*; *Spirochaetales* | 66.2 | (YP_003802215) NAD(P)-dependent iron-only hydrogenase catalytic subunit [*Spirochaeta smaragdinae* DSM 11293] |  |
| P28_hydA12 | D4ZHLFP1:60:D2HDNACXX:2:2304:20198:65356:GGATGT | *Spirochaetes*; *Spirochaetales* | 73.2 | (YP_003802215) NAD(P)-dependent iron-only hydrogenase catalytic subunit [*Spirochaeta smaragdinae* DSM 11293] |  |
| P28_hydA125 | D4ZHLFP1:60:D2HDNACXX:2:1302:18912:63674:GGATGT | *Spirochaetes*; *Spirochaetales* | 93.6 | (YP_003802215) NAD(P)-dependent iron-only hydrogenase catalytic subunit [*Spirochaeta smaragdinae* DSM 11293] |  |
| P28_hydA136 | D4ZHLFP1:60:D2HDNACXX:2:1316:16229:71422:GGATGT | *Spirochaetes*; *Spirochaetales* | 62.4 | (YP_003802215) NAD(P)-dependent iron-only hydrogenase catalytic subunit [*Spirochaeta smaragdinae* DSM 11293] |  |
| P28_hydA148 | D4ZHLFP1:60:D2HDNACXX:2:2313:15953:28598:GGATGT | *Spirochaetes*; *Spirochaetales* | 73.9 | (YP_005475514) hydrogenase, Fe-only [*Spirochaeta africana* DSM 8902] |  |
| P28_hydA161 | D4ZHLFP1:60:D2HDNACXX:2:1102:12280:94355:GGATGT | *Spirochaetes*; *Spirochaetales* | 66.6 | (YP_006044637) hydrogenase, Fe-only [*Spirochaeta* *thermophila* DSM 6578] |  |
| P28_hydA185 | D4ZHLFP1:60:D2HDNACXX:2:2311:19480:99240:GGATGT | *Spirochaetes*; *Spirochaetales* | 92.8 | (YP_003802215) NAD(P)-dependent iron-only hydrogenase catalytic subunit [*Spirochaeta smaragdinae* DSM 11293] |  |
| P28_hydA189 | D4ZHLFP1:60:D2HDNACXX:2:1309:11913:24310:GGATGT | *Spirochaetes*; *Spirochaetales* | 60.8 | (YP_006044637) hydrogenase, Fe-only [*Spirochaeta* *thermophila* DSM 6578] |  |
| P28_hydA19 | D4ZHLFP1:60:D2HDNACXX:2:1309:2702:32676:GGATGT | *Spirochaetes*; *Spirochaetales* | 73.2 | (YP_005475514) hydrogenase, Fe-only [*Spirochaeta africana* DSM 8902] |  |
| P28_hydA163 | D4ZHLFP1:60:D2HDNACXX:2:1114:3565:23768:GGATGT | *Thermotogae*; *Thermotogales* | 88.6 | (YP_002939902) hydrogenase, Fe-only [*Kosmotoga olearia* TBF 19.5.1] |  |
| P28_hydA255 | D4ZHLFP1:60:D2HDNACXX:2:2311:13944:72440:GGATGT | *Thermotogae*; *Thermotogales* | 70.9 | (YP_002939902) hydrogenase, Fe-only [*Kosmotoga olearia* TBF 19.5.1] |  |
| P28_hydA257 | D4ZHLFP1:60:D2HDNACXX:2:1309:3954:78696:GGATGT | *Thermotogae*; *Thermotogales* | 79.7 | (YP_002939902) hydrogenase, Fe-only [*Kosmotoga olearia* TBF 19.5.1] |  |
| P28_hydA265 | D4ZHLFP1:60:D2HDNACXX:2:2110:8245:52454:GGATGT | *Thermotogae*; *Thermotogales* | 82.4 | (YP_005470551) hydrogenase, Fe-only [*Fervidobacterium pennivorans* DSM 9078] |  |
| P28_hydA343 | D4ZHLFP1:60:D2HDNACXX:2:1316:10526:19921:GGATGT | *Thermotogae*; *Thermotogales* | 73.6 | (YP_005096723) hydrogenase, Fe-only [*Marinitoga piezophila* KA3] |  |
| P28_hydA373 | D4ZHLFP1:60:D2HDNACXX:2:2315:7515:50488:GGATGT | *Thermotogae*; *Thermotogales* | 82.4 | (YP_002939902) hydrogenase, Fe-only [*Kosmotoga olearia* TBF 19.5.1] |  |
| P28_hydA385 | D4ZHLFP1:60:D2HDNACXX:2:1309:15282:15883:GGATGT | *Thermotogae*; *Thermotogales* | 79.7 | (YP_005096723) hydrogenase, Fe-only [*Marinitoga piezophila* KA3] |  |
| P28_hydA424 | D4ZHLFP1:60:D2HDNACXX:2:2312:16959:78448:GGATGT | *Thermotogae*; *Thermotogales* | 72.8 | (YP_002939902) hydrogenase, Fe-only [*Kosmotoga olearia* TBF 19.5.1] |  |
| P28_hydA70 | D4ZHLFP1:60:D2HDNACXX:2:2106:4783:93650:GGATGT | *Thermotogae*; *Thermotogales* | 64.3 | (YP_005470551) hydrogenase, Fe-only [*Fervidobacterium pennivorans* DSM 9078] |  |
| P28_hydA72 | D4ZHLFP1:60:D2HDNACXX:2:2303:6153:42186:GGATGT | *Thermotogae*; *Thermotogales* | 87.4 | (YP_002939902) hydrogenase, Fe-only [*Kosmotoga olearia* TBF 19.5.1] |  |
| P28_hydA22 | D4ZHLFP1:60:D2HDNACXX:2:2313:16296:62472:GGATGT | *Thermotogae*; *Thermotogales* | 56.2 | (YP_001409760) hydrogenase, Fe-only [*Fervidobacterium nodosum* Rt17-B1] |  |
| P28_hydA438 | D4ZHLFP1:60:D2HDNACXX:2:1108:4211:76573:GGATGT | *Verrucomicrobia*; *Opitutales* | 61.6 | (YP_001818428) hydrogenase, Fe-only [*Opitutus* *terrae* PB90-1] |  |
| P28_hydA245 | D4ZHLFP1:60:D2HDNACXX:2:2311:16033:61947:GGATGT | Paddy field soil, Japan | 72.8 | (BAM65975) [FeFe]-hydrogenase, partial [uncultured bacterium] |  |
| P28_hydA263 | D4ZHLFP1:60:D2HDNACXX:2:1316:4581:3244:GGATGT | Paddy field soil, Japan | 83.6 | (BAM65873) [FeFe]-hydrogenase, partial [uncultured bacterium] |  |
| P28_hydA264 | D4ZHLFP1:60:D2HDNACXX:2:2316:10927:9465:GGATGT | Paddy field soil, Japan | 82.4 | (BAM66122) [FeFe]-hydrogenase, partial [uncultured bacterium] |  |
| P28_hydA270 | D4ZHLFP1:60:D2HDNACXX:2:1113:12148:29276:GGATGT | Paddy field soil, Japan | 82.8 | (BAM65975) [FeFe]-hydrogenase, partial [uncultured bacterium] |  |
| P28_hydA219 | D4ZHLFP1:60:D2HDNACXX:2:1108:5767:25663:GGATGT | Paddy field soil, Japan | 61.2 | (BAM65896) [FeFe]-hydrogenase, partial [uncultured bacterium] |  |
| P28_hydA220 | D4ZHLFP1:60:D2HDNACXX:2:1105:12304:22767:GGATGT | Paddy field soil, Japan | 74.7 | (BAM65896) [FeFe]-hydrogenase, partial [uncultured bacterium] |  |
| P28_hydA235 | D4ZHLFP1:60:D2HDNACXX:2:2306:12066:75508:GGATGT | Paddy field soil, Japan | 75.1 | (BAM65873) [FeFe]-hydrogenase, partial [uncultured bacterium] |  |
| P28_hydA169 | D4ZHLFP1:60:D2HDNACXX:2:2305:3576:73977:GGATGT | Paddy field soil, Japan | 77.8 | (BAM66149) [FeFe]-hydrogenase, partial [uncultured bacterium] |  |
| P28_hydA131 | D4ZHLFP1:60:D2HDNACXX:2:1303:20757:91605:GGATGT | Paddy field soil, Japan | 85.5 | (BAM66039) [FeFe]-hydrogenase, partial [uncultured bacterium] |  |
| P28_hydA452 | D4ZHLFP1:60:D2HDNACXX:2:2302:6322:95743:GGATGT | Paddy field soil, Japan | 75.9 | (BAM66084) [FeFe]-hydrogenase, partial [uncultured bacterium] |  |
| P28_hydA128 | D4ZHLFP1:60:D2HDNACXX:2:1106:16034:41315:GGATGT | Paddy field soil, Japan | 68.6 | (BAM66175) [FeFe]-hydrogenase, partial [uncultured bacterium] |  |
| P28_hydA174 | D4ZHLFP1:60:D2HDNACXX:2:1112:5107:21749:GGATGT | Paddy field soil, Japan | 77.4 | (BAM65896) [FeFe]-hydrogenase, partial [uncultured bacterium] |  |
| P28_hydA201 | D4ZHLFP1:60:D2HDNACXX:2:2301:20855:80465:GGATGT | Paddy field soil, Japan | 85.9 | (BAM65965) [FeFe]-hydrogenase, partial [uncultured bacterium] |  |
| P28_hydA104 | D4ZHLFP1:60:D2HDNACXX:2:1109:9142:36449:GGATGT | Paddy field soil, Japan | 91.3 | (BAM66117) [FeFe]-hydrogenase, partial [uncultured bacterium] |  |
| P28_hydA107 | D4ZHLFP1:60:D2HDNACXX:2:2304:3378:12314:GGATGT | Paddy field soil, Japan | 79.7 | (BAM66175) [FeFe]-hydrogenase, partial [uncultured bacterium] |  |
| P28_hydA3 | D4ZHLFP1:60:D2HDNACXX:2:1104:13776:58918:GGATGT | Paddy field soil, Japan | 80.1 | (BAM66149) [FeFe]-hydrogenase, partial [uncultured bacterium] |  |
| P28_hydA34 | D4ZHLFP1:60:D2HDNACXX:2:1113:8868:88278:GGATGT | Paddy field soil, Japan | 84.0 | (BAM66175) [FeFe]-hydrogenase, partial [uncultured bacterium] |  |
| P28_hydA346 | D4ZHLFP1:60:D2HDNACXX:2:2311:17889:19912:GGATGT | Paddy field soil, Japan | 68.2 | (BAM65975) [FeFe]-hydrogenase, partial [uncultured bacterium] |  |
| P28_hydA37 | D4ZHLFP1:60:D2HDNACXX:2:1301:11581:79121:GGATGT | Paddy field soil, Japan | 66.2 | (BAM65845) [FeFe]-hydrogenase, partial [uncultured bacterium] |  |
| P28_hydA377 | D4ZHLFP1:60:D2HDNACXX:2:1313:9467:66814:GGATGT | Paddy field soil, Japan | 78.2 | (BAM66088) [FeFe]-hydrogenase, partial [uncultured bacterium] |  |
| P28_hydA380 | D4ZHLFP1:60:D2HDNACXX:2:2103:7160:7503:GGATGT | Paddy field soil, Japan | 72.4 | (BAM65896) [FeFe]-hydrogenase, partial [uncultured bacterium] |  |
| P28_hydA384 | D4ZHLFP1:60:D2HDNACXX:2:2111:5306:87159:GGATGT | Paddy field soil, Japan | 81.3 | (BAM65965) [FeFe]-hydrogenase, partial [uncultured bacterium] |  |
| P28_hydA52 | D4ZHLFP1:60:D2HDNACXX:2:1310:6679:37596:GGATGT | Paddy field soil, Japan | 94.7 | (BAM66117) [FeFe]-hydrogenase, partial [uncultured bacterium] |  |
| P28_hydA53 | D4ZHLFP1:60:D2HDNACXX:2:1105:12680:81935:GGATGT | Paddy field soil, Japan | 93.2 | (BAM66088) [FeFe]-hydrogenase, partial [uncultured bacterium] |  |
| P28_hydA54 | D4ZHLFP1:60:D2HDNACXX:2:1315:12857:40287:GGATGT | Paddy field soil, Japan | 86.7 | (BAM66117) [FeFe]-hydrogenase, partial [uncultured bacterium] |  |
| P28_hydA78 | D4ZHLFP1:60:D2HDNACXX:2:2309:1879:88698:GGATGT | Paddy field soil, Japan | 85.1 | (BAM65896) [FeFe]-hydrogenase, partial [uncultured bacterium] |  |
| P28_hydA82 | D4ZHLFP1:60:D2HDNACXX:2:1111:13288:34560:GGATGT | Paddy field soil, Japan | 79.3 | (BAM65896) [FeFe]-hydrogenase, partial [uncultured bacterium] |  |
| P28_hydA89 | D4ZHLFP1:60:D2HDNACXX:2:1306:8669:10318:GGATGT | Paddy field soil, Japan | 49.7 | (BAM66154) [FeFe]-hydrogenase, partial [uncultured bacterium] |  |
| P28_hydA112 | D4ZHLFP1:60:D2HDNACXX:2:1104:16777:32264:GGATGT | Paddy field soil, Japan | 86.3 | (BAM65896) [FeFe]-hydrogenase, partial [uncultured bacterium] |  |
| P28_hydA155 | D4ZHLFP1:60:D2HDNACXX:2:1101:19725:90289:GGATGT | Paddy field soil, Japan | 79.3 | (BAM65896) [FeFe]-hydrogenase, partial [uncultured bacterium] |  |
| P28_hydA244 | D4ZHLFP1:60:D2HDNACXX:2:2109:20719:77364:GGATGT | Paddy field soil, Japan | 94.7 | (BAM66232) [FeFe]-hydrogenase, partial [uncultured bacterium] |  |
| P28_hydA28 | D4ZHLFP1:60:D2HDNACXX:2:1107:21082:64239:GGATGT | Paddy field soil, Japan | 79.0 | (BAM65975) [FeFe]-hydrogenase, partial [uncultured bacterium] |  |
| P28_hydA99 | D4ZHLFP1:60:D2HDNACXX:2:1110:1283:40053:GGATGT | Paddy field soil, Japan | 90.5 | (BAM66117) [FeFe]-hydrogenase, partial [uncultured bacterium] |  |
| P28_hydA337 | D4ZHLFP1:60:D2HDNACXX:2:1313:15865:35313:GGATGT | Paddy field soil, Japan | 81.6 | (BAM66175) [FeFe]-hydrogenase, partial [uncultured bacterium] |  |
| P28_hydA439 | D4ZHLFP1:60:D2HDNACXX:2:2315:5629:86143:GGATGT | Paddy field soil, Japan | 80.1 | (BAM65973) [FeFe]-hydrogenase, partial [uncultured bacterium] |  |
| P28_hydA49 | D4ZHLFP1:60:D2HDNACXX:2:2306:14493:52422:GGATGT | Paddy field soil, Japan | 86.7 | (BAM66042) [FeFe]-hydrogenase, partial [uncultured bacterium] |  |
| P28_hydA94 | D4ZHLFP1:60:D2HDNACXX:2:1316:14939:47564:GGATGT | Paddy field soil, Japan | 78.6 | (BAM66088) [FeFe]-hydrogenase, partial [uncultured bacterium] |  |
| P28_hydA216 | D4ZHLFP1:60:D2HDNACXX:2:2310:20634:25267:GGATGT | Paddy field soil, Japan | 99.4 | (BAM66088) [FeFe]-hydrogenase, partial [uncultured bacterium] |  |
| P28_hydA415 | D4ZHLFP1:60:D2HDNACXX:2:2110:9798:11682:GGATGT | Paddy field soil, Japan | 90.1 | (BAM66173) [FeFe]-hydrogenase, partial [uncultured bacterium] |  |
| P28_hydA360 | D4ZHLFP1:60:D2HDNACXX:2:1311:19109:53485:GGATGT | Paddy field soil, Japan | 77.0 | (BAM65965) [FeFe]-hydrogenase, partial [uncultured bacterium] |  |
| P28_hydA77 | D4ZHLFP1:60:D2HDNACXX:2:2310:3887:27231:GGATGT | Paddy field soil, Japan | 83.2 | (BAM66084) [FeFe]-hydrogenase, partial [uncultured bacterium] |  |
| P28_hydA207 | D4ZHLFP1:60:D2HDNACXX:2:2111:5017:69311:GGATGT | Paddy field soil, Japan | 68.9 | (BAM65975) [FeFe]-hydrogenase, partial [uncultured bacterium] |  |
| P28_hydA430 | D4ZHLFP1:60:D2HDNACXX:2:2312:11983:94515:GGATGT | Paddy field soil, Japan | 98.6 | (BAM66117) [FeFe]-hydrogenase, partial [uncultured bacterium] |  |
| P28_hydA239 | D4ZHLFP1:60:D2HDNACXX:2:2114:3737:84393:GGATGT | Oil fields subdJected to CO_2_ and water-flooding | 91.0 | (AGU38562) FeFe-hydrogenase, partial [uncultured prokaryote] |  |
| P28_hydA275 | D4ZHLFP1:60:D2HDNACXX:2:1311:18238:99456:GGATGT | Oil fields subdJected to CO_2_ and water-flooding | 67.4 | (AGU38562) FeFe-hydrogenase, partial [uncultured prokaryote] |  |
| P28_hydA347 | D4ZHLFP1:60:D2HDNACXX:2:2115:11101:29212:GGATGT | Oil fields subdJected to CO_2_ and water-flooding | 98.0 | (AGU38562) FeFe-hydrogenase, partial [uncultured prokaryote] |  |
| P28_hydA397 | D4ZHLFP1:60:D2HDNACXX:2:1305:14634:49470:GGATGT | Oil fields subdJected to CO_2_ and water-flooding | 92.8 | (AGU38562) FeFe-hydrogenase, partial [uncultured prokaryote] |  |
| P28_hydA417 | D4ZHLFP1:60:D2HDNACXX:2:1112:12296:56649:GGATGT | Oil fields subdJected to CO_2_ and water-flooding | 62.0 | (AGU38565) FeFe-hydrogenase, partial [uncultured prokaryote] |  |
| P28_hydA181 | D4ZHLFP1:60:D2HDNACXX:2:2315:7477:90219:GGATGT | Oil fields subdJected to CO_2_ and water-flooding | 99.0 | (AGU38562) FeFe-hydrogenase, partial [uncultured prokaryote] |  |
| P28_hydA133 | D4ZHLFP1:60:D2HDNACXX:2:1315:18802:34017:GGATGT | Oil fields subdJected to CO_2_ and water-flooding | 92.0 | (AGU38562) FeFe-hydrogenase, partial [uncultured prokaryote] |  |
| P28_hydA319 | D4ZHLFP1:60:D2HDNACXX:2:2305:7572:15250:GGATGT | Oil fields subdJected to CO_2_ and water-flooding | 92.0 | (AGU38562) FeFe-hydrogenase, partial [uncultured prokaryote] |  |
| P28_hydA350 | D4ZHLFP1:60:D2HDNACXX:2:2314:8112:100475:GGATGT | Oil fields subdJected to CO_2_ and water-flooding | 72.4 | (AGU38572) FeFe-hydrogenase, partial [uncultured prokaryote] |  |
| P28_hydA356 | D4ZHLFP1:60:D2HDNACXX:2:2309:11330:76283:GGATGT | Oil fields subdJected to CO_2_ and water-flooding | 78.2 | (AGU38562) FeFe-hydrogenase, partial [uncultured prokaryote] |  |
| P28_hydA59 | D4ZHLFP1:60:D2HDNACXX:2:2104:21112:2451:GGATGT | Oil fields subdJected to CO_2_ and water-flooding | 75.9 | (AGU38562) FeFe-hydrogenase, partial [uncultured prokaryote] |  |
| P28_hydA318 | D4ZHLFP1:60:D2HDNACXX:2:1303:1708:62889:GGATGT | Oil fields subdJected to CO_2_ and water-flooding | 96.7 | (AGU38562) FeFe-hydrogenase, partial [uncultured prokaryote] |  |
| P28_hydA409 | D4ZHLFP1:60:D2HDNACXX:2:2305:14962:93264:GGATGT | Oil fields subdJected to CO_2_ and water-flooding | 93.6 | (AGU38562) FeFe-hydrogenase, partial [uncultured prokaryote] |  |
| P28_hydA61 | D4ZHLFP1:60:D2HDNACXX:2:2312:9596:27580:GGATGT | Oil fields subdJected to CO_2_ and water-flooding | 92.0 | (AGU38562) FeFe-hydrogenase, partial [uncultured prokaryote] |  |
| P28_hydA111 | D4ZHLFP1:60:D2HDNACXX:2:2315:8361:45741:GGATGT | Oil fields subdJected to CO_2_ and water-flooding | 96.7 | (AGU38562) FeFe-hydrogenase, partial [uncultured prokaryote] |  |
| P28_hydA170 | D4ZHLFP1:60:D2HDNACXX:2:2113:3747:95005:GGATGT | Saline microbial mat community, Mexico | 95.9 | (ACM67558) iron-dependent hydrogenase [uncultured organism] |  |
| P28_hydA122 | D4ZHLFP1:60:D2HDNACXX:2:2114:4910:91263:GGATGT | Saline microbial mat community, Mexico | 97.4 | (ACM67558) iron-dependent hydrogenase [uncultured organism] |  |
| P28_hydA426 | D4ZHLFP1:60:D2HDNACXX:2:2315:9602:86691:GGATGT | Saline microbial mat community, Mexico | 93.2 | (ACM67558) iron-dependent hydrogenase [uncultured organism] |  |
| P28_hydA204 | D4ZHLFP1:60:D2HDNACXX:2:2316:11718:88709:GGATGT | Saline microbial mat community, Mexico | 91.3 | (ACM67558) iron-dependent hydrogenase [uncultured organism] |  |
| P28_hydA443 | D4ZHLFP1:60:D2HDNACXX:2:2306:6557:86865:GGATGT | Acidic fen | 86.7 | (CAY56136) [Fe-Fe] hydrogenase large subunit [uncultured bacterium] |  |
| P28_hydA213 | D4ZHLFP1:60:D2HDNACXX:2:1311:19242:44860:GGATGT | Acidic fen | 90.1 | (CAY56138) [Fe-Fe] hydrogenase large subunit [uncultured bacterium] |  |
| P28_hydA457 | D4ZHLFP1:60:D2HDNACXX:2:1310:6776:26173:GGATGT | Water column of the Great Salt Lake, USA | 95.5 | (ADC53611) [Fe-Fe] hydrogenase [uncultured bacterium] |  |
| P28_hydA90 | D4ZHLFP1:60:D2HDNACXX:2:1314:14350:90820:GGATGT | Water column of the Great Salt Lake, USA | 92.4 | (ADC53611) [Fe-Fe] hydrogenase [uncultured bacterium] |  |
| P28_hydA114 | D4ZHLFP1:60:D2HDNACXX:2:2305:14681:91239:GGATGT | Earthworm gut contents | 67.0 | (CBX44190) [FeFe]-hydrogenase large subunit, partial [uncultured bacterium] |  |
| P28_hydA182 | D4ZHLFP1:60:D2HDNACXX:2:2302:19922:61154:GGATGT | Phreatic limestone sinkho*les* in Mexico | 57.4 | (ACQ94905) [Fe-Fe] hydrogenase [uncultured bacterium] |  |
| P28_hydA359 | D4ZHLFP1:60:D2HDNACXX:2:2107:7975:61393:GGATGT | Biogas digester system | 62.4 | (AEK22103) FeFe-hydrogenase [uncultured organism] |  |
| P28_hydA410 | D4ZHLFP1:60:D2HDNACXX:2:2308:6226:88233:GGATGT | Anaerobic domestic sewage sludge | 82.8 | (AGF90866) FeFe-hydrogenase large subunit, partial [uncultured bacterium] |  |
| P28_hydA7 | D4ZHLFP1:60:D2HDNACXX:2:2108:1486:27443:GGATGT | Biogas digester system | 85.9 | (AET11788) Fe-hydrogenase [uncultured organism] |  |
| P28_hydA179 | D4ZHLFP1:60:D2HDNACXX:2:1306:2483:74069:GGATGT | Biogas digester system | 70.1 | (AET11789) hydrogenase [uncultured organism] |  |
| P28_hydA431 | D4ZHLFP1:60:D2HDNACXX:2:2307:14244:49092:GGATGT | Water column of the Great Salt Lake, USA | 88.2 | (ADQ86057) [FeFe]-hydrogenase subunit A large subunit [uncultured bacterium] |  |
| P28_hydA335 | D4ZHLFP1:60:D2HDNACXX:2:1303:8667:76848:GGATGT | Termite gut microbiota | 65.5 | (AFH54065) [Fe-Fe] hydrogenase large subunit, partial [uncultured bacterium] |  |
