# Supplementary material for: Metagenomic and PCR-Based Diversity Surveys of [FeFe]-Hydrogenases Combined with Isolation of Alkaliphilic Hydrogen-Producing Bacteria from the Serpentinite-Hosted Prony Hydrothermal Field, New Caledonia
Source: Front Microbiol. 2016 Aug 30;7:1301. doi: 10.3389/fmicb.2016.01301 (PMC5003875; doi:10.3389/fmicb.2016.01301)
Supplement: Supplementary file 3 [file Table3.DOC]

**Table S3. Richness and diversity of 16S rRNA bacterial genes and [Fe-Fe]-hydrogenase *hydA* genes in Prony hydrothermal springs,** namely 'Bain des Japonais' (BdJ), 'Rivière des Kaoris' (RK) and 'Aiguille de Prony' (ST07)**.** Suffixes ‘C’and ‘F’ in site names stand respectively for chimney and fluid.

| Samples | Genes | Sequences | OTUs | Coverage | Chao1 | Shannon | Simpson |
| --- | --- | --- | --- | --- | --- | --- | --- |
| BDJC | 16S rRNA | 4360 | 323 | 0.926 | 326.3 | 4.0 | 0.640 |
| BDJF | 16S rRNA | 4360 | 125 | 0.971 | 163.2 | 3.3 | 0.826 |
| RKC | 16S rRNA | 4360 | 161 | 0.963 | 190.1 | 3.9 | 0.843 |
| RKF | 16S rRNA | 3490 | 143 | 0.959 | 145.2 | 5.1 | 0.938 |
| ST07 | 16S rRNA | 3925 | 217 | 0.945 | 227.5 | 5.6 | 0.950 |
| BDJC | *hydA* | 52 | 10 | 0.808 | 15 | 1.45 | 0.369 |
| BDJF | *hydA* | 61 | 10 | 0.836 | 16 | 1.70 | 0.248 |
| RKC | *hydA* | 28 | 5 | 0.821 | 5 | 1.24 | 0.357 |
| RKF | *hydA* | 26 | 2 | 0.923 | 2 | 0.17 | 0.92 |
| ST07 | *hydA* | 14 | 5 | 0.643 | 5 | 1.25 | 0.330 |
